# Development of *N*-F fluorinating agents and their fluorinations: Historical perspective

**DOI:** 10.3762/bjoc.17.123

**Published:** 2021-07-27

**Authors:** Teruo Umemoto, Yuhao Yang, Gerald B Hammond

**Affiliations:** 1 Department Chemistry, University of Louisville, Lousiville, Kentucky 40292, USA

**Keywords:** N-F fluorinating agent, fluorination, fluorodiazoniabicyclo[2.2.2]octane salt, fluoropyridinium salt, fluorosulfonimide

## Abstract

This review deals with the historical development of all *N*-F fluorinating agents developed so far. The unique properties of fluorine make fluorinated organic compounds attractive in many research areas and therefore fluorinating agents are important. *N*-F agents have proven useful by virtue of their easy handling. This reagent class includes many types of *N*-F compounds: perfluoro-*N*-fluoropiperidine, *N*-fluoro-2-pyridone, *N*-fluoro-*N*-alkylarenesulfonamides, *N*-fluoropyridinium salts and derivatives, *N*-fluoroquinuclidium salts, *N*-fluoro-trifluoromethanesulfonimide, *N*-fluoro-sultams, *N*-fluoro-benzothiazole dioxides, *N*-fluoro-lactams, *N*-fluoro-*o*-benzenedisulfonimide, *N*-fluoro-benzenesulfonimide, 1-alkyl-4-fluoro-1,4-diazoniabicyclo[2.2.2]octane salts, *N*-fluoropyridinium-2-sulfonate derivatives, 1-fluoro-4-hydroxy-1,4-diazoniabicyclo[2.2.2]octane salts, *N*-fluorodinitroimidazole, *N*-fluoro-trichloro-1,3,5-triazinium salt, *N*-F ethano-Tröger’s base derivatives, *N*-fluoro-methanesulfonimide, *N*-fluoro-*N*-arylarenesulfonamides, bis*N*-F salts such as *N*,*N’*-difluorobipyridinium salts and *N*,*N’*-difluoro-1,4-diazoniabicyclo[2.2.2]octane salts, and their many derivatives and analogs, including chiral *N*-F reagents such as optically active *N*-fluoro-sultam derivatives, *N*-fluoro-alkaloid derivatives, DABCO-based *N*-F derivatives, and *N*-F binaphthyldisulfonimides. The synthesis and reactions of these reagents are described chronologically and the review also discusses the relative fluorination power of each reagent and their mechanisms chronicling developments from a historical perspective.

## Introduction

Fluorinated organic compounds occupy an important position in pharmaceuticals [1], agrochemicals [2], and materials [3]. Especially, in the first two areas, the presence of fluorine has attracted attention during the last decades. Nowadays, a considerable number of medicines [4–5] and agrochemicals [6] contain at least one fluorine atom in their structures. The fluorine atom has unique properties such as the highest electronegativity, extremely low polarization, strong C–F bonds, and the smallest size after a hydrogen atom [7]. Thus, introduction of fluorine into selective positions of a bioactive compound can produce remarkable changes in efficacy. Fluorine-scan/fluorine editing of a lead molecule is now a routine step in drug discovery [8]. Organofluorine compounds are very rare in nature [9] and therefore without natural compounds, chemical processes are required to generate building blocks. Molecular fluorine (F_2_) is a useful fluorinating reagent, however, unlike Cl_2_ and Br_2_, F_2_ is extremely reactive, toxic, and corrosive and its handling requires specialist skills and equipment. Therefore, easy-to-handle and selective fluorinating agents are essential for the wide-spread advancement of organofluorine chemistry to non-specialist chemists. Alternatives to F_2_, such as perchloryl fluoride (FClO_3_) [10] and the *O*-F reagents such as CF_3_OF [11], CF_2_(OF)_2_ [11], CsOSO_2_OF [12], CF_3_COOF [13], and CH_3_COOF [14] have been used as fluorinating agents for many years. However, these reagents have significant risks for safe handling. Although XeF_2_ [15] was considered as a safer alternative, it is expensive because of the scarcity of Xe in nature. The appearance of the safe and easy-to-handle *N*-F fluorinating agents described in this review have brought about a breakthrough in synthetic fluorine chemistry enabling an increasing number of researchers to engage in organofluorine chemistry. Their development has significantly contributed towards the current ‘golden age’ of fluorine chemistry. The *N*-F fluorinating agents now stand out as particularly useful electrophilic or radical fluorinating agents by virtue of their easy handling, efficiency, and selectivity. These non-hygroscopic nature and stability make them easier to handle than nucleophilic fluoride reagents. Potassium fluoride (KF) and naked fluoride anion salts are extremely sensitive to moisture, while HF seriously attacks human skin. The *N*-F fluorinating agents can be classified into two categories: these are neutral and cationic. This review covers the chronological advancement of these reagents regardless of their classification, as they advanced side by side.

## Review

### 1. Historical progress of *N*-F fluorinating agents

#### 1-1. Perfluoro-*N*-fluoropiperidine

**Scheme 1:**
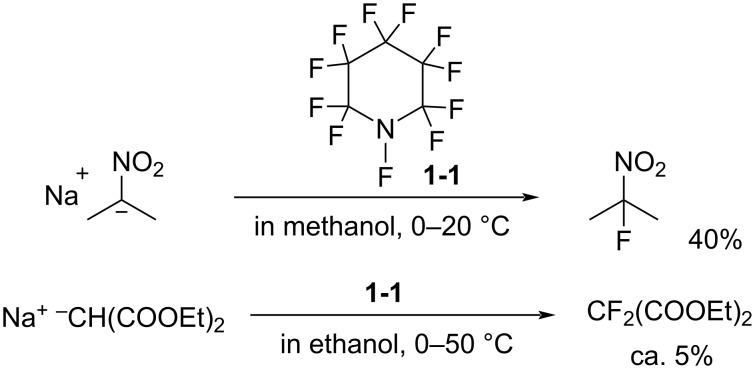
Fluorination with *N*-F amine **1-1**.

The history of the *N*-F compounds acting as fluorine atom-transfer reagents can be traced back to 1964 when Banks and co-worker [16] first reported that perfluoro-*N*-fluoropiperidine (**1-1**) could fluorinate the sodium salt of 2-nitropropane to form 2-fluoro-2-nitropropane in a 40% yield (Scheme 1). The reaction with sodium diethyl malonate was also reported to produce the difluoromalonate, but in a very low yield of ca. 5%.

**Scheme 2:**
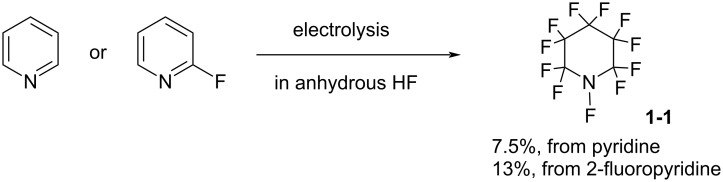
Preparation of *N*-F amine **1-1**.

*N*-F amine **1-1** is very volatile (bp 49.5 °C), and could only be prepared in 7.5% or 13% yield by electrochemical fluorination of pyridine or 2-fluoropyridine in anhydrous hydrogen fluoride [17–18] (Scheme 2). Not surprisingly, **1-1** did not become a popular reagent.

**Scheme 3:**
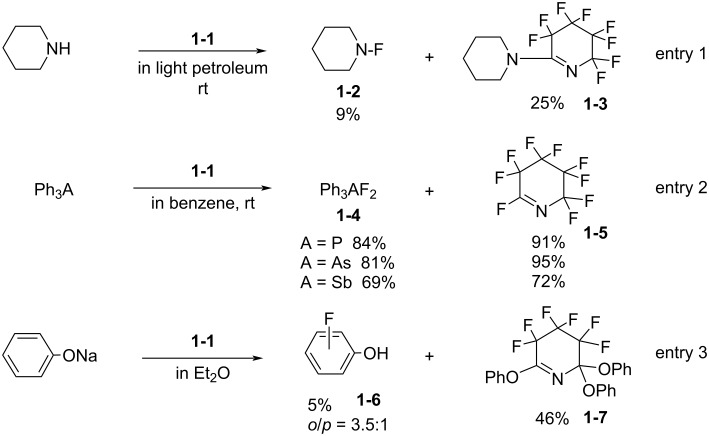
Reactions of *N*-F amine **1-1**.

In 1967, Banks et al. reported reactions of **1-1** with piperidine and triphenylphosphine, -arsine, and -stibine (Scheme 3, entries 1 and 2) [19]. The former reaction gave adduct **1-3** as a major product and the latter showed good fluorine-transfer to the hetero atoms. In 1972, German et al. reported the reaction with sodium phenoxide, which gave a small amount (5%) of fluorophenols **1-6** and a larger amount of adduct **1-7** (Scheme 3, entry 3) [20]. These data showed that the fluorinations with **1-1** were suppressed by the formation of perfluoro-3,4,5,6-tetrahydropyridine (**1-5**), which was reactive to the nucleophilic substrates existing in the reaction mixture.

In 1986, Banks and co-worker reported the preparation of polymeric analogues of perfluoro-*N*-fluoropiperidine (**1-1**) [21] and then in 1991, Banks et al. reported the improved yields of the reactions of **1-1** with sodium salts of 2-nitropropane, malonate esters, and a keto ester, and phenylmagnesium bromide [22]. However, the fluorinated products were still accompanied by considerable amounts of byproducts resulting from the reaction of the substrates with **1-5**.

#### 1-2. *N*-Fluoroperfluorosuccinimide and *N*-fluoroperfluoroglutarimide

**Scheme 4:**
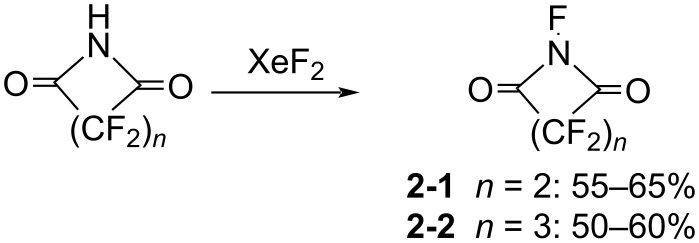
Synthesis of *N*-F perfluoroimides **2-1** and **2**-**2**.

In 1981, Yagupols’kii and co-worker reported the synthesis of *N*-fluoroperfluorosuccinimide (**2-1**) and *N*-fluoroperfluoroglutarimide (**2-2**) by reaction of precursor imides with XeF_2_ (Scheme 4) [23]. However, there were no reports on the fluorination capability of these *N*-F compounds. The purpose of this research was to establish if the presence of perfluoroacyl groups was sufficient to stabilize the Xe–N bond. Their experiment revealed that the intermediate F–Xe-N compounds were not detected, but *N*-fluoroimides **2-1** and **2-2** were formed. The stability of these *N*-F compounds was low, since they decomposed to H_2_NCO(CF_2_)*_n_*COOH upon standing with air.

#### 1-3. 1-Fluoro-2-pyridone

**Scheme 5:**
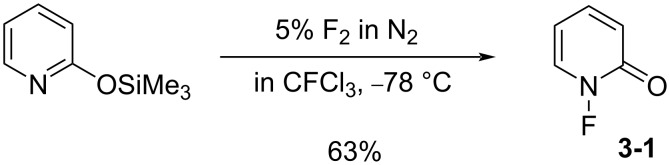
Synthesis of 1-fluoro-2-pyridone (**3-1**).

In 1983, Purrington and co-worker synthesized 1-fluoro-2-pyridone (**3-1**) as a fluorination agent [24]. When 2-(trimethylsiloxy)pyridine was allowed to react with 5% F_2_ diluted with N_2_ in a freon solvent at −78 °C, 1-fluoro-2-pyridone was obtained in 63% yield (Scheme 5).

**Scheme 6:**
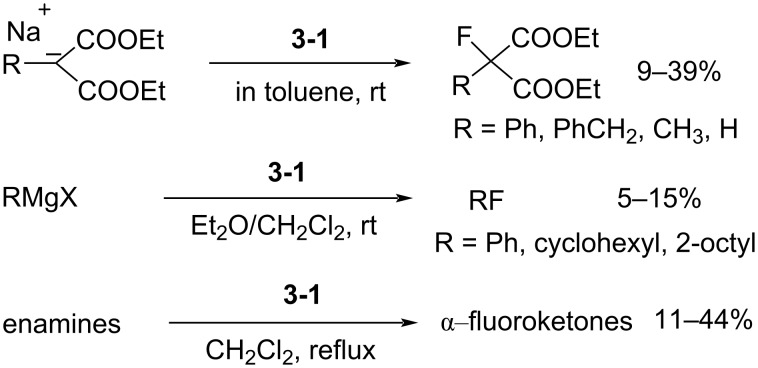
Fluorination with 1-fluoro-2-pyridone (**3-1**).

The fluorination efficiency of **3-1** was higher than perfluoro-*N*-fluoropiperidine (**1-1**) and the yields of reaction with sodium diethyl malonates improved to between 9–39%; a difluorinated byproduct was obtained when the substituent R was H (Scheme 6). Further applications were investigated by the same authors in 1984 [25]. Grignard reagents and enamines could be fluorinated, but in very low yields and unfortunately, this reagent was not so stable.

Notably, attempts to prepare *N*-fluorosuccinimide from succinimide, or one of its salts by reaction with fluorine (F_2_), trifluoromethyl hypofluorite, or perchloryl fluoride in a variety of solvents, and at temperatures ranging from −78 °C to room temperature, all but failed, as reported in the margin of the paper cited in [24].

#### 1-4. *N*-Fluoro-*N*-alkylarenesulfonamides

**Figure 1:**
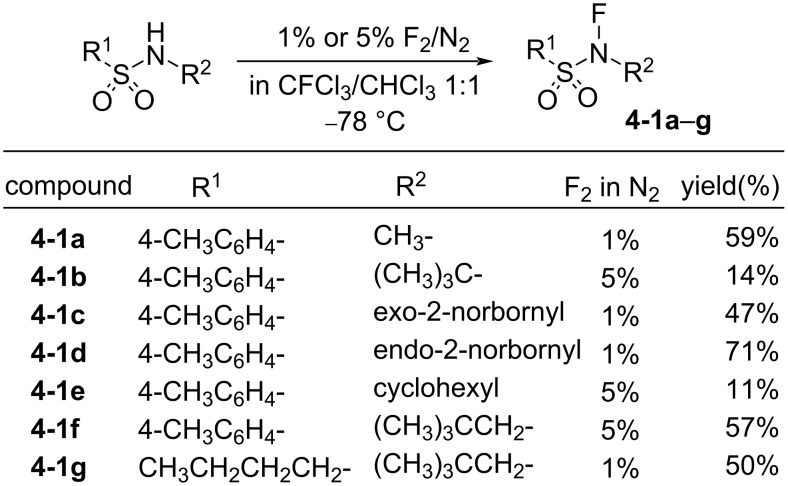
Synthesis of *N*-F sulfonamides **4-1a**–**g**.

In 1984, a series of stable *N*-fluoro-*N*-alkylsulfonamides **4-1a–g** was reported by Barnette [26]. The treatment of *N*-alkylsulfonamides with very dilute F_2_ (1% or 5%) in N_2_ at −78 °C afforded the fluorinated products **4-1a–g**. As detailed in Figure 1, various kinds of *N*-fluoro-*N*-alkylsulfonamides were synthesized by this method. However, the yields were low except for the case of compound **4-1d**. In the cases of secondary and tertiary alkyl groups on the amine side, low yields were obtained and these were attributed to concomitant N–S-bond cleavage reactions with F_2_. This method, using the dilute F_2_, was inefficient for their production due to long reaction times.

**Scheme 7:**
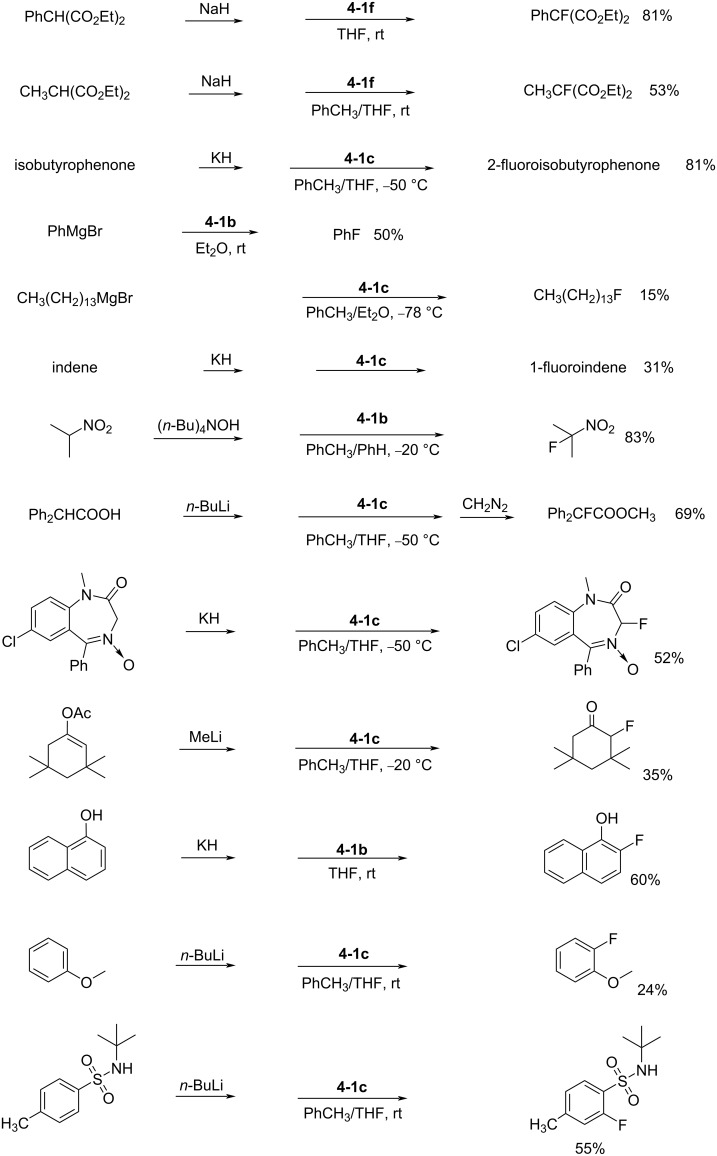
Fluorination with *N*-F reagent **4-1b**,**c**,**f**.

*N*-Fluoro-*N*-alkyl-*p*-toluenesulfonamides **4-1b**,**c**,**f** proved to be efficient fluorinating agents in the fluorination of carbanions (Scheme 7). The yields of reactions with sodium malonates and Grignard reagents were largely improved to up to 81% and 50%, respectively. The carbanions of aromatics, ketones, nitroalkanes, amides, etc. could also be reasonably well fluorinated and this study showed great progress. However, although these fluorinating agents were stable and easy-to-handle, their fluorinating power was low. They could fluorinate only reactive carbanions, but not aromatics, olefins, vinyl acetates, trimethylsilyl or alkyl enol ethers, and so on.

**Scheme 8:**
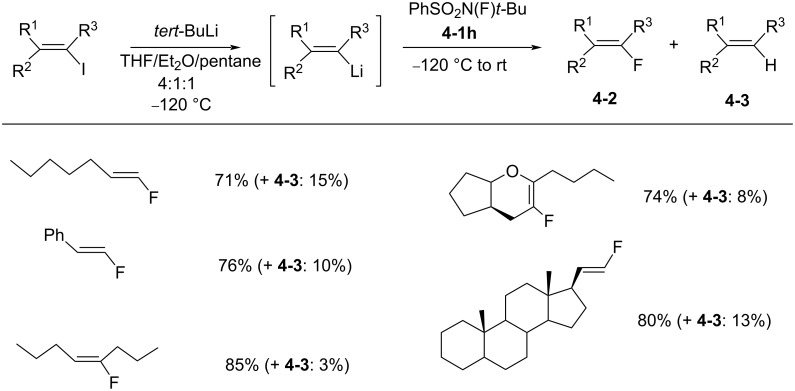
Fluorination of alkenyllithiums with *N*-F **4-1h**.

Soon after **(**1986), Schwartz and co-worker reported the stereospecific synthesis of alkenyl fluorides with *N*-fluoro-*N*-*tert*-butylbenzenesulfonamide (**4-1h**), a compound which is soluble at low temperature [27]. Alkenyllithium reagents, generated in situ, reacted at −120 °C with **4-1h** in THF/Et_2_O/pentane to give the desired alkenyl fluorides **4-2** in good yields (Scheme 8). However, reactions that were run above −120 °C, or in pure ether or THF gave higher yields of the protonated products **4-3**.

#### 1-5. *N*-Fluoropyridinium salts and their derivatives

In 1986, Umemoto et al. reported *N*-fluoropyridinium triflate and its derivatives **5-4** as new stable cationic fluorinating agents. These possessed either electron-donating or -withdrawing substituents on the pyridinium nuclei, and were the first reactive, easy-to-handle fluorinating agents with wide application [28–29]. The work continued with additional disclosures until 1991 [30–34]. Before that, reactive fluorinating reagents were difficult to handle because of toxicity, a tendency to explode, instability, and/or hygroscopicity, while easy-to-handle reagents had limited application because of their low reactivity.

**Scheme 9:**
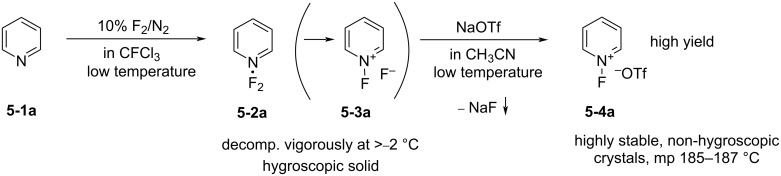
Synthesis of *N*-fluoropyridinium triflate (**5-4a**).

Umemoto et al. found that the hygroscopic pyridine·F_2_ complex **5-2a** decomposing vigorously at temperatures above −2 °C [35], which was formed by the fluorination of pyridine (10% F_2_/N_2_) at low temperature in a freon solvent, could undergo straightforward counteranion replacement with a non-nucleophilic anion. Therefore, exchange with salts such as sodium triflate in acetonitrile generated non-hygroscopic *N*-fluoropyridinium triflate salts as highly thermally stable reagents as illustrated in Scheme 9 [28]. Moreover, they found that the fluorination power (reactivity) of these *N*-fluoropyridinium salts could be tuned by the substituents on the pyridinium nuclei.

**Scheme 10:**
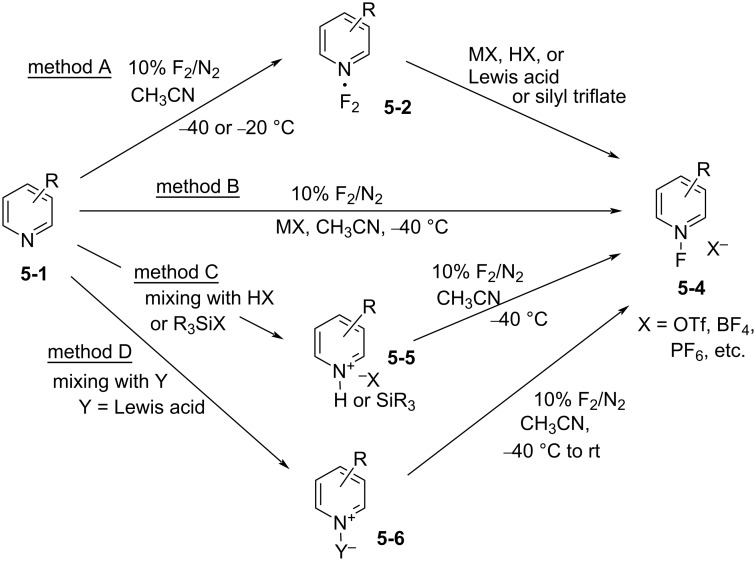
Synthetic methods for *N*-F-pyridinium salts.

**Figure 2:**
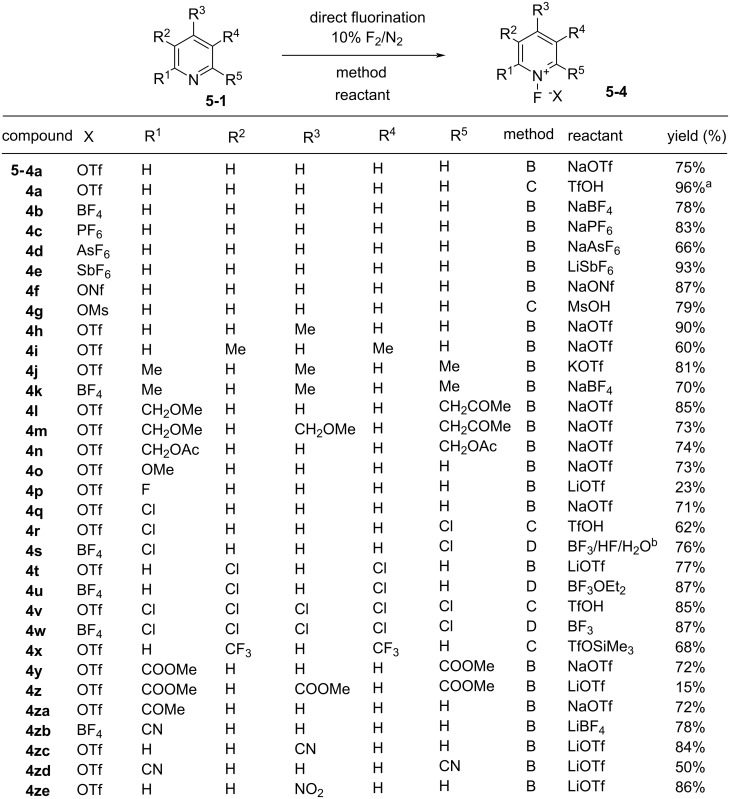
Synthesis of various *N*-fluoropyridinium salts. Note: ^a^this yield was the one by the improved method reported in [37]; ^b^this improved method was reported in [38].

The transformation of the unstable pyridine·F_2_ complex to stable *N*-fluoropyridinium salts could be conducted by direct fluorination (10% F_2_/N_2_) of the pyridine in acetonitrile and in the presence of a suitable salt at low temperature (method B in Scheme 10). This one-step process was successfully applied to many other pyridine derivatives (method B in Figure 2).

It was finally shown that the stable *N*-fluoropyridinium salts **5-4** could be synthesized in four different ways using 10% F_2_/N_2_ [28,31,33] (Scheme 10): method A (stepwise method) involved the fluorination of a pyridine derivative with F_2_/N_2_, to form a pyridine·F_2_ complex **5-2**, followed by treatment with a non-nucleophilic anion salt, acid, or silyl derivative. Method B (one-step method) involved the fluorination of a pyridine derivative with F_2_/N_2_ in the presence of a non-nucleophilic anion salt. Method C involved mixing a pyridine derivative with an acid or its silyl derivative, forming a salt **5-5**, before fluorination. Method D involved mixing a pyridine derivative with a Lewis acid, forming complex **5-6**, and then the fluorination with F_2_/N_2_ was carried out.

In total, sixty-two stable *N*-fluoropyridinium salts possessing different non-nucleophilic counteranions and electron-withdrawing or -donating groups were efficiently synthesized [33]. Figure 2 shows 31 examples and their methods of preparation. Umemoto and co-worker also reported the synthesis of a polymer version, poly(vinyl-*N*-fluoropyridinium salts) of these reagents [36].

**Scheme 11:**
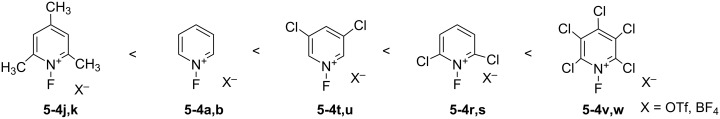
Fluorination power order of *N*-fluoropyridinium salts.

The reactivities of many *N*-fluoropyridinium salts were examined [32] and mainly five kinds of *N*-fluoropyridinium salts, shown in Scheme 11, emerged as useful fluorinating agents due to their availability. The fluorination power greatly changed depending on the electron density at the nitrogen, an aspect controlled by the electronic nature of the substituents. The fluorinating power increased in the order of 2,4,6-triMe **5-4j** < unsubstituted **4a** < 3,5-diCl **4t** < 2,6-diCl **4r** < pentachloro **4v**, in good agreement with the decreasing order of the p*K*_a_ values of the pyridines. For example, in order to fluorinate phenol, triMe **5-4j** needed heating at 100 °C in a haloalkane solvent for 24 h, whereas pentachloro **5-4v** required only room temperature within 0.1 h for a successful reaction. The salt **5-4v** was so powerful that it fluorinated an equimolar amount of benzene in dichloromethane in 2 h at 40 °C. In general, triflate salts were more effective than BF_4_ salts because of the higher solubility of the triflate salts in a haloalkane solvent.

**Scheme 12:**
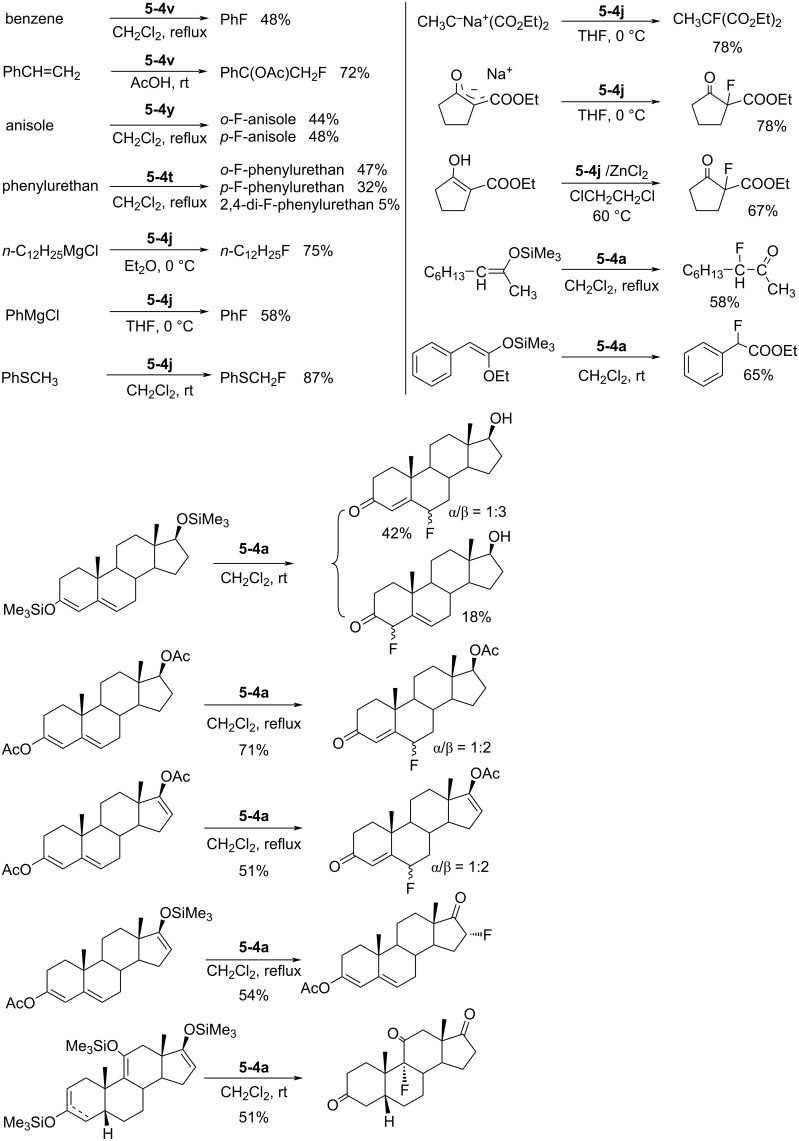
Fluorinations with *N*-F salts **5-4**.

As outlined in Scheme 12, fluorinations of many kinds of substrates with these *N*-fluoropyridinium salts were performed. It was shown that less reactive substrates can be fluorinated well with the more powerful reagents, and reactive substrates can be fluorinated with less powerful reagents, a match process which minimizes side reactions. Thus, these *N*-fluoropyridinium salts made possible the fluorination of a diversity of nucleophilic organic compounds with different reactivities ranging across aromatics, carbanions (Grignard reagents, enolate anions), active methylene compounds, olefins, silyl enol ethers, vinyl acetates, sulfides and so on, under mild conditions with high selectivity and yields [29–32]. All these reactions could be carried out routinely using standard glassware in normal laboratory environments and without any specialist training.

**Scheme 13:**
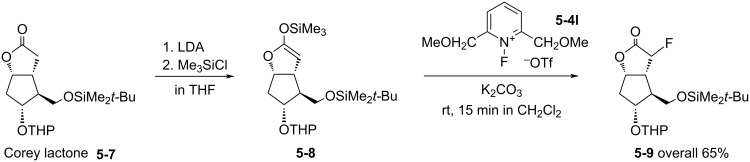
Fluorination of Corey lactone **5-7** with *N*-F-bis(methoxymethyl) salt **5-4l**.

Some interesting observations are noted: *N*-Fluoro-2,6-bis(methoxymethyl)pyridinium triflate (**5-4l)** fluorinated the trimethylsilyl ether of γ-butyrolactone and 1-cyclohexenyl acetate in much higher yields than the *N*-fluoro-2,4,6-trimethyl salt **5-4j** [32]. Thus, as seen in Scheme 13, **5-4l** converted the Corey lactone **5-7** via its silyl ether **5-8** to the fluorinated lactone **5-9** in a very satisfactory overall yield [32]. *N*-Fluoro-2,6-bis(CH_2_OAc)pyridinium triflate **5-4n** [32], *N*-fluoro-2,6-bis(COOMe)pyridinium triflate **5-4y** [30,32], and *N*-fluoro-2-cyano- and -2,6-dicyanopyridinium tetrafluoroborates [39] were useful reagents, too. These non-chlorinated reagents avoid chlorinated byproducts, an occurrence that was observed when other powerful fluorinating agents such as *N*-fluoro-2,3,4,5,6-pentachloropyridinium salts were used [40].

**Scheme 14:**
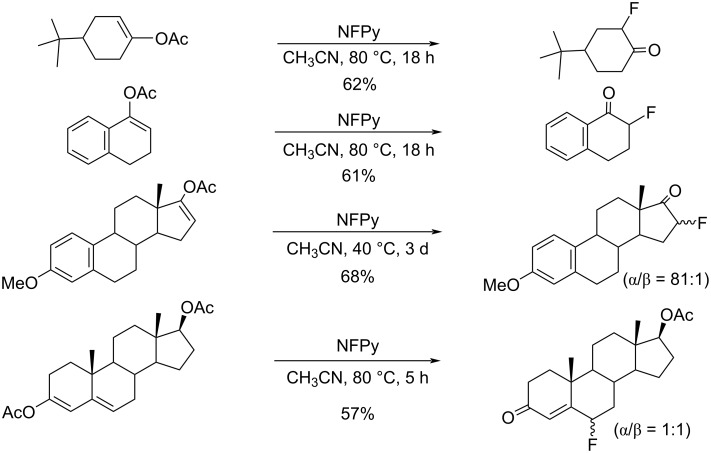
Fluorination with NFPy.

In 1991, *N*-fluoropyridinium pyridine heptafluorodiborate (NFPy), C_5_H_5_NF(C_5_H_5_N)B_2_F_7_, was introduced as a fluorinating agent [41]. NFPy was prepared by the reaction of fluorine with pyridine·BF_3_ complex and its fluorination ability is shown in Scheme 14. However, it was subsequently reported that the correct structure was *N*-fluoropyridinium pyridinium tetrafluoroborate trifluorohydroxyborate, C_5_H_5_NF(C_5_H_5_NH)BF_4_(BF_3_OH), which was a 1:1 mixture of the *N*-fluoropyridinium salt and *N*-hydropyridinium salt (anion parts; BF_4_ and BF_3_OH), based on an X-ray diffraction study of a commercial sample [42].

#### 1-6. *N*-Fluoroquinuclidinium fluoride

**Scheme 15:**
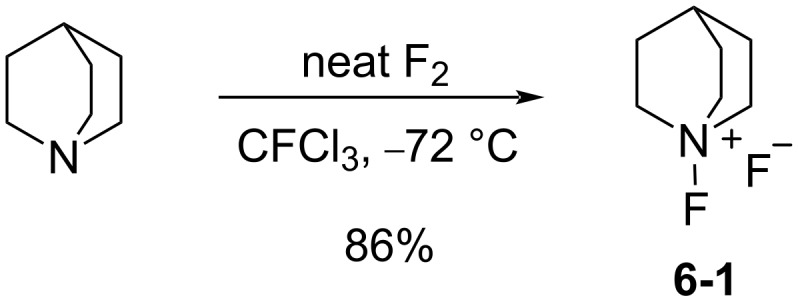
Synthesis of the *N*-F reagent, *N*-fluoroquinuclidinium fluoride (**6-1**).

**Scheme 16:**
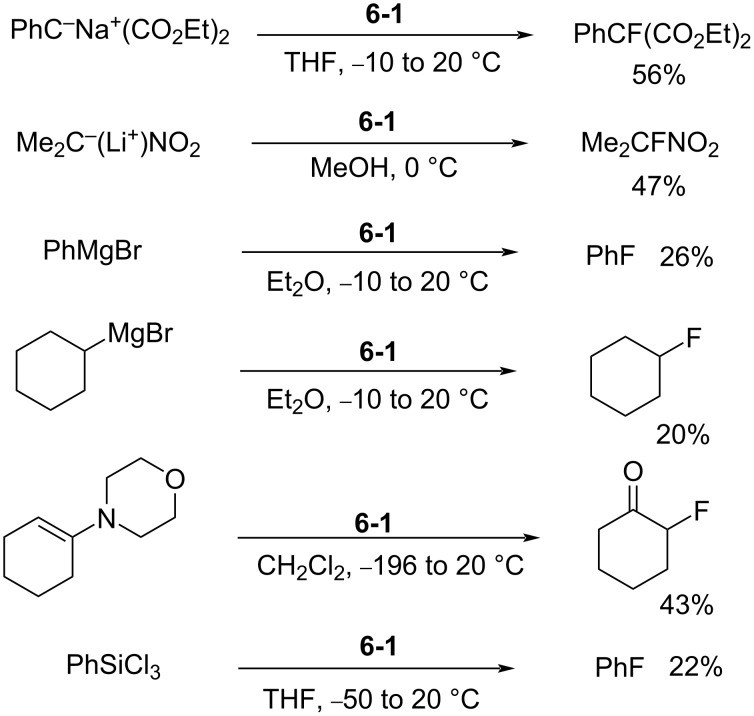
Fluorinations achieved with *N*-F fluoride **6**-**1**.

In 1986 as the *N*-F pyridinium reagents were emerging, Banks et al. disclosed the quaternary ammonium *N*-F reagent, *N*-fluoroquinuclidinium fluoride (**6-1**) [43]. They subsequently followed with more detailed results in 1988 [44]. Quinuclidine was fluorinated by neat fluorine in trichlorofluoromethane at −72 °C, affording the product **6-1** in 86% yield (Scheme 15). Fluorination examples with **6-1** are shown in Scheme 16.

This reagent proved to be very hygroscopic and deteriorated in air. In terms of fluorination yields, *N*-fluoroquinuclidinium fluoride (**6-1**) was however superior to Purrington’s 1-fluoro-2-pyridone (**3-1**) [24–25], but inferior to Barnette’s *N*-fluoro-*N*-alkylarenesulfonamides **4-1** [26] and Umemoto’s *N*-fluoropyridinium salts **5-4** [29,32].

#### 1-7. *N*-Fluoroperfluoroalkanesulfonimides

**Scheme 17:**
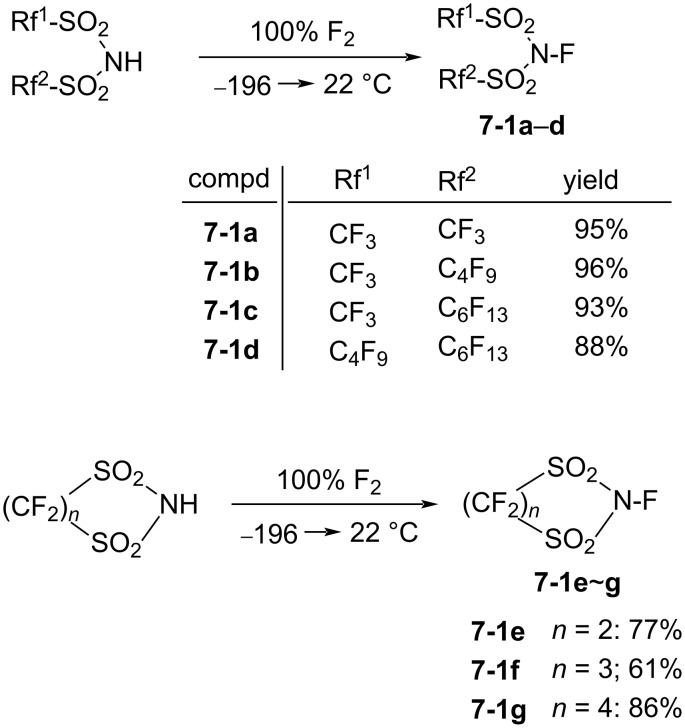
Synthesis of *N*-F imides **7-1a**–**g**.

In 1987, DesMarteau et al. reported the synthesis of a series of *N*-fluoroperfluoroalkanesulfonimides **7-1**. These were prepared by reacting *N,N*-bis(perfluoroalkanesulfonyl)amides with 100% F_2_ at −196 °C to 22 °C [45] (Scheme 17). All of these *N*-fluoroperfluoroalkanesulfonimides were stable over time at 22 °C, if stored in a fluoropolymer container. However, **7-1d** decomposed at the melting point of 60 °C.

**Scheme 18:**
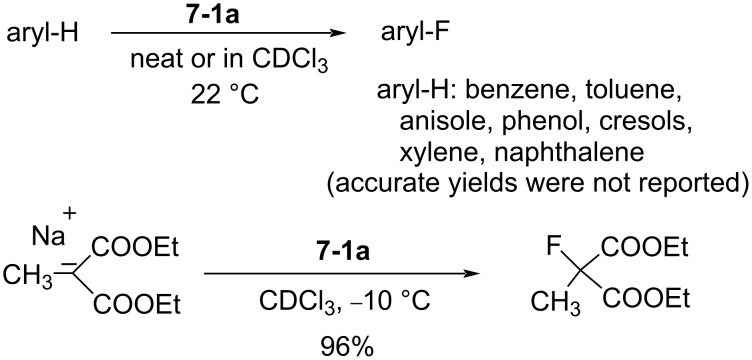
Fluorination with (CF_3_SO_2_)_2_NF, **7-1a**.

It was shown that *N*-fluorotrifluoromethanesulfonimide **7-1a** (mp −69.8 °C) had a much higher reactivity than the sulfonamide reagents such as Barnette’s *N*-fluoro-*N*-alkylarenesulfonamides, since the electronic density on the nitrogen was greatly decreased by two strong electron-withdrawing CF_3_SO_2_ groups. Reagent **7-1a** reacted slowly with benzene and toluene under neat conditions, whereas activated aromatics such as phenol, cresol, and naphthalene were fluorinated in chloroform at 22 °C (Scheme 18). The *N*-F imide reagent **7-1a** fluorinated the sodium salt of diethyl 1-methylmalonate at −10 °C to give the corresponding fluoro product in high yield (96%).

**Scheme 19:**
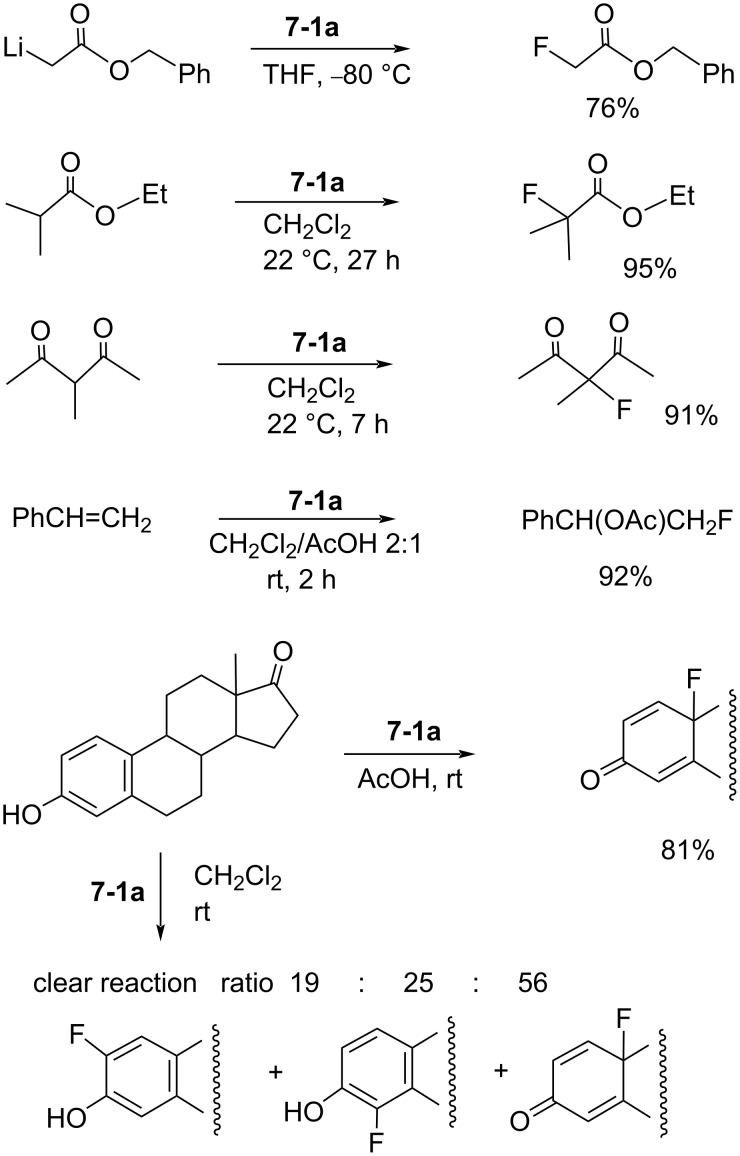
Fluorination reactions of various substrates with **7-1a**.

Later (1991 and 1992), the same laboratory reported fluorination reactions of functionalized carbonyl compounds, 1,3-dicarbonyl derivatives, olefins, and steroids with **7-1a** [46–49] (Scheme 19).

#### 1-8. *N*-Fluoroquinuclidinium triflate

**Scheme 20:**
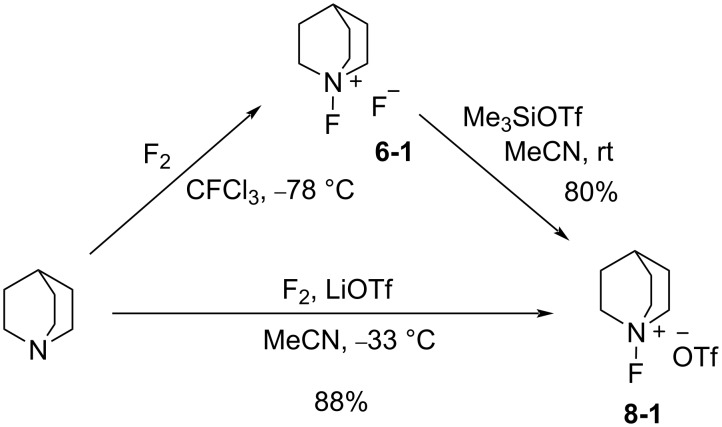
Synthesis of *N*-F triflate **8-1**.

In 1988, Banks and co-worker developed the stable and nonhygroscopic *N*-fluoroquinuclidinium triflate (**8-1**) [50], which was an alternative to *N*-fluoroquinuclidinium fluoride (**6-1**) (Scheme 20). The triflate **8-1** was prepared in high yield by the counteranion replacement reaction developed by Umemoto and co-worker [28]. The fluorinating power of triflate **8-1** was the same as that of the fluoride **6-1**, but its nonhygroscopic nature made it a useful fluorinating agent in terms of handling and storage. Details on the synthesis and reactivities of triflate **8-1** and other salts having CF_3_COO^−^, C_3_F_7_COO^−^, and BF_4_^−^ counterions were reported in 1991 [51].

#### 1-9. Optically active *N*-fluorosultams

**Scheme 21:**
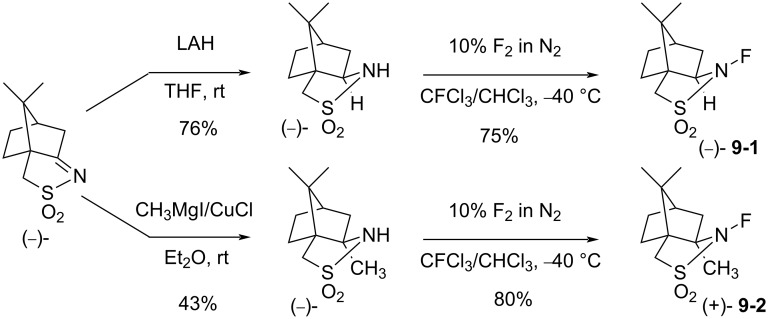
Synthesis of chiral *N*-fluoro sultams **9-1** and **9-2**.

In 1988, the first optically active *N*-F fluorinating agents, chiral *N*-fluorosultams, were synthesized by Lang and co-worker [52]. A camphor-derived imine was reduced or methylated, followed by direct fluorination (10% F_2_/N_2_) to give optically active *N*-F reagents **9-1** and **9-2** in 75% and 80% yield, respectively (Scheme 21). These *N*-fluorosultams were stable below 100 °C.

**Scheme 22:**
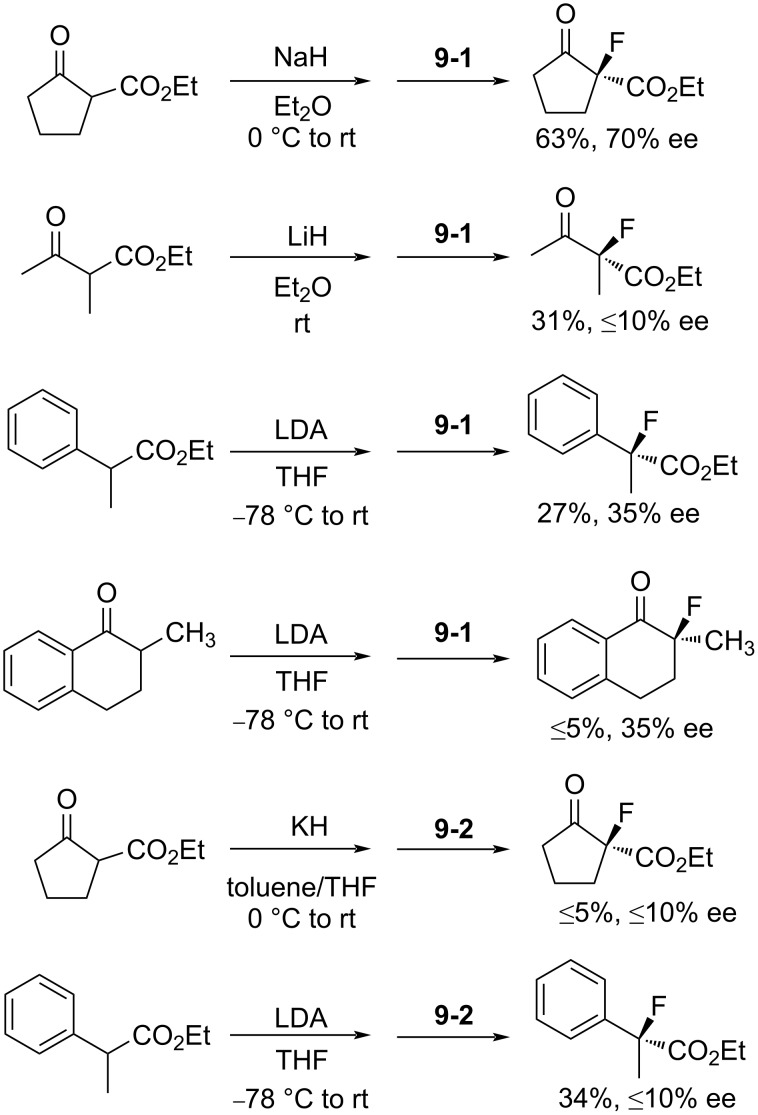
Fluorination with chiral *N*-fluoro sultams **9-1** and **9-2**.

The enantioselectivities of products were examined after the fluorination of different metal enolates. In the best case a 63% yield and 70% enantiomeric excess (ee) was obtained. Even though other products gave less satisfactory outcomes, the potential of the *N*-F fluorinating agents for an enantioselective fluorination was demonstrated (Scheme 22).

#### 1-10. *N*-Fluoro-3,3-dimethylbenzothiazole dioxide

**Scheme 23:**
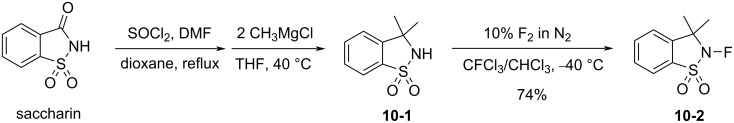
Synthesis of saccharin-derived *N*-fluorosultam **10-2**.

In 1989, Lang and co-worker developed the saccharin-derived *N*-fluorosultam *N*-fluoro-3,3-dimethyl-2,3-dihydro-1,2-benzothiazole-1,1-dioxide (**10-2**) from the known precursor **10-1** (Scheme 23) [53]. A direct fluorination using 10% F_2_/N_2_ at −40 °C gave *N*-F reagent **10-2** in 74% yield. Another method, via the *N*-trimethylsilylation, was also reported but in low efficiency. This *N*-F reagent is a colorless, thermally stable (<200 °C) solid with a mp of 114–116 °C.

**Scheme 24:**
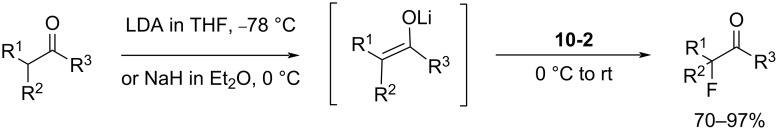
Fluorination with *N*-fluorosultam **10-2**.

The reagent **10-2** having no α-proton to the *N*-F site proved to be a good choice for fluorinating enolate anions (Scheme 24). The side reaction, involving HF elimination, and which was a problem in reactions with the Barnette’s reagents **4-1** having the α-proton(s) except for **4-1b** [26], was avoided here. The HF elimination is a decomposition process that is observed with *N*-F reagents that have an α-proton and occurs under strong base conditions.

#### 1-11. Perfluoro[*N*-fluoro-*N*-(4-pyridyl)methanesulfonamide]

**Scheme 25:**
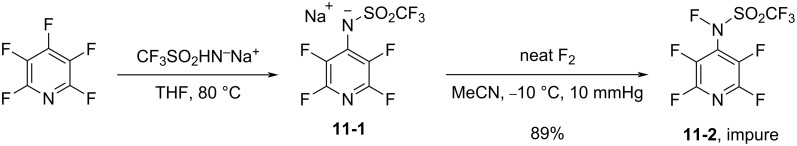
Synthesis of *N*-F reagent **11-2**.

In 1990, Banks and co-worker reported perfluoro[*N*-fluoro-*N*-(4-pyridyl)methanesulfonamide] (**11-2**) [54]. Starting from pentafluoropyridine, the precursor **11-1** was prepared as illustrated in Scheme 25. Treatment of **11-1** with neat F_2_ in acetonitrile at −10 °C under reduced pressure gave *N*-fluoro-sulfonamide **11-2** in 89% yield. This product was however a 9:1 mixture of the *N*-F reagent **11-2** and the protonated compound of **11-1**.

**Scheme 26:**
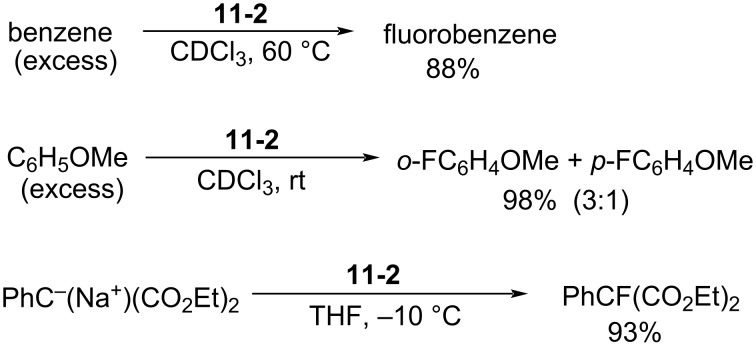
Fluorination with *N*-F reagent **11-2**.

The fluorination of benzene and anisole under excess substrate conditions gave fluorobenzene and fluoroanisoles in 88% and 98% yield, respectively. The reaction with sodium diethyl phenylmalonate gave the fluorinated product in 93% yield (Scheme 26).

#### 1-12. *N*-Fluorolactams

**Scheme 27:**
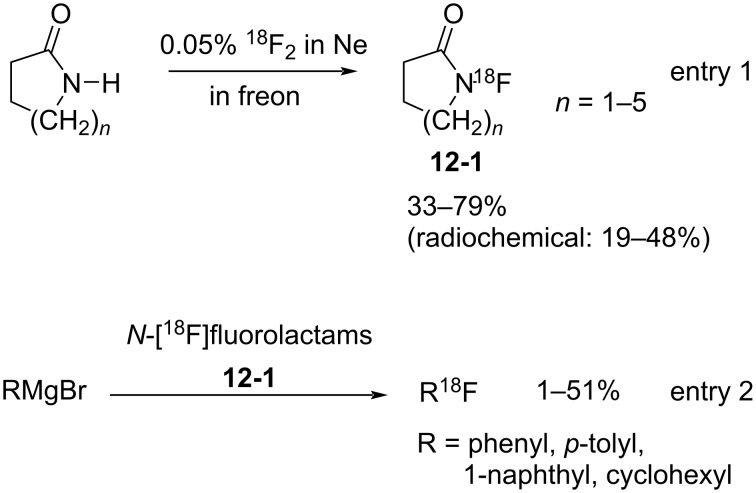
Synthesis and reaction of *N*-fluorolactams **12-1**.

A new class of *N*-F fluorinating agents, *N*-fluorolactams **12-1**, was synthesized by Sathyamurthy et al. in 1990 [55]. These compounds had already been prepared by Grakauskas and co-worker in 1970 [56], but in low yields and the *N*-fluorolactams were not recognized as fluorinating agents at that time. For positron emission tomography (PET), Sathyamurthy et al. allowed these lactams to react with 0.05% ^18^F_2_/Ne in a freon and obtained the *N*-[^18^F]fluorolactams **12-1** in good yields (Scheme 27, entry 1). The ^18^F-transfer ability was demonstrated by fluorination reactions with various Grignard reagents in up to 51% yield (Scheme 27, entry 2). In the event the β-elimination of HF proved to be an obstacle for the fluorination of strong bases such as phenyllithium.

#### 1-13. *N*-Fluoro-*o*-benzenedisulfonimide

**Scheme 28:**
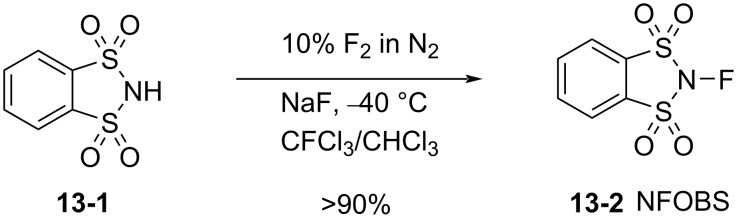
Synthesis of NFOBS **13-2**.

In 1991, Davis and co-worker reported *N*-fluoro-*o*-benzenedisulfonimide (NFOBS, **13-2**) as a fluorination reagent. NFOBS was prepared by the direct fluorination of its corresponding sulfonimide **13-1** with 10% F_2_/N_2_ in the presence of NaF and in high yield (Scheme 28) [57].

**Scheme 29:**
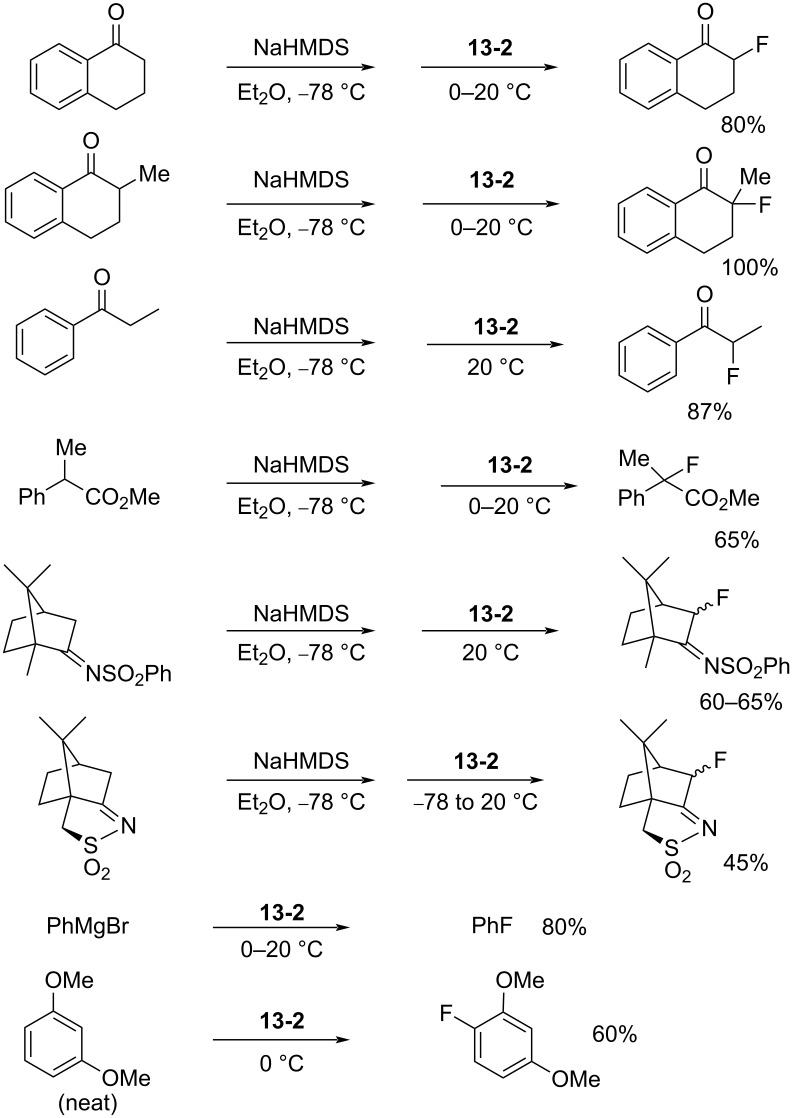
Fluorination with NFOBS **13-2**.

NFOBS is a stable and crystalline solid of mp 139–140 °C (dec) with good fluorinating power. Its usefulness was demonstrated through fluorination reactions with different types of carbanions (Scheme 29). For example, reactions with enolates gave α-fluorinated products in 65–100% yields. Azaenolates could be fluorinated in moderate yields. A fluorination reaction with phenylmagnesium bromide provided fluorobenzene in 80% yield, an outcome which was better than that of *N*-fluoropyridinium triflate **5-4j** (Umemoto’s reagent, 58%) and *N*-fluorosulfonamide **4-1b** (Barnette’s reagent, 50%). NFOBS was able to fluorinate 1,3-dimethoxybenzene under neat conditions, while attempts to fluorinate toluene and acetophenone failed. The details of these reactions and applications were described in a full paper later published in 1995 [58].

#### 1-14. *N*-Fluorobenzenesulfonimide (NFSI)

**Scheme 30:**
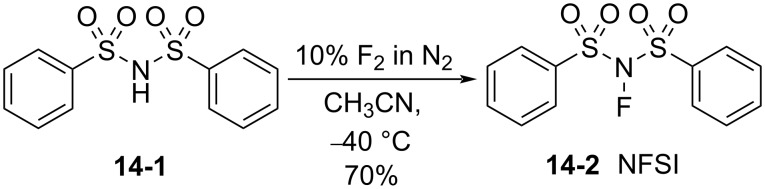
Synthesis of NFSI (**14-2**).

In 1991, *N*-fluorobenzenesulfonimide (NFSI, **14-2**) was synthesized by Differding and co-worker [59]. It was prepared from benzenesulfonimide **14-1** in good yield by its reaction with 10% F_2_/N_2_ in acetonitrile at −40 °C (Scheme 30). NFSI is a stable and non-hygroscopic crystalline solid with a mp of 114–116 °C.

**Scheme 31:**
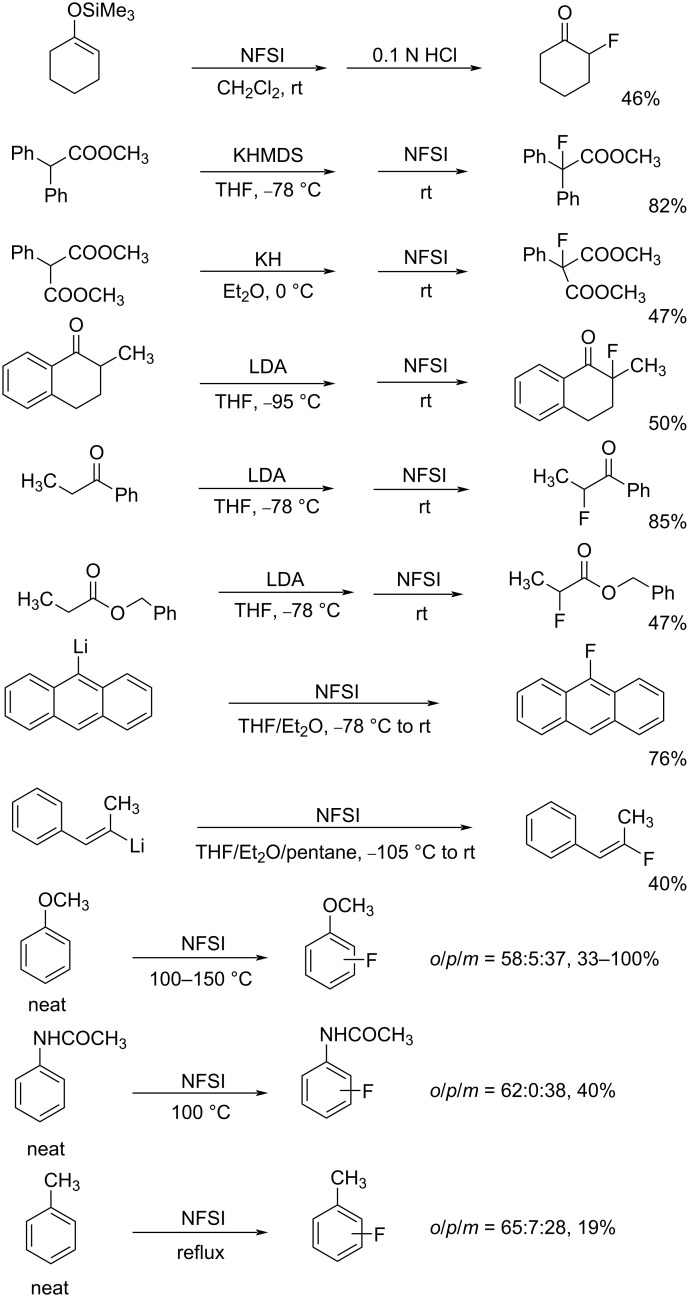
Fluorination with NFSI **14-2**.

NFSI was shown to fluorinate a variety of nucleophiles. As seen in Scheme 31, trimethylsilyl enol ethers, enolate anions of ketones and esters, and aryl- and vinyllithiums were fluorinated with NFSI in moderate to high yields. Although aromatics such as anisole, toluene, and acetanilide could also be fluorinated by NFSI, these reactions required neat conditions and high temperatures, indicating that NFSI was not so powerful.

#### 1-15. *N*-Fluorosaccharin and *N*-fluorophthalimide

**Scheme 32:**
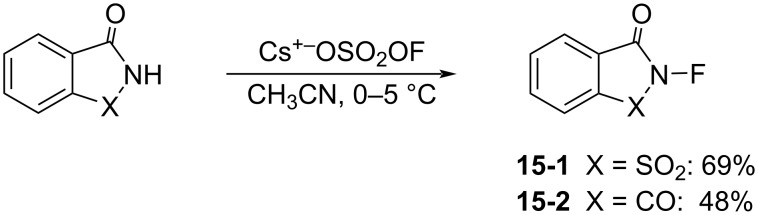
Synthesis of *N*-fluorosaccharin (**15-1**) and *N*-fluorophthalimide (**15-2**).

In 1991, Gakh et al. reported the synthesis of *N*-fluorosaccharin (**15-1**) and *N*-fluorophthalimide (**15**-**2**) in their studies on the reactivity of cesium fluoroxysulfate (Cs^+−^OSO_2_OF) [60]. Sodium salts of saccharin and phthalimide reacted with cesium fluoroxysulfate in acetonitrile at 0–5 °C to give **15-1** and **15-2** in 69% and 48% yields, respectively (Scheme 32). However, they did not report any fluorination reactions with **15-1** and **15-2**. A transfer reaction failed to generate *N*-fluorosuccinimide from the reaction of the sodium salt of succinimide with cesium fluoroxysulfate.

#### 1-16. 1-Alkyl-4-fluoro-1,4-diazoniabicyclo[2.2.2]octane salts

**Scheme 33:**
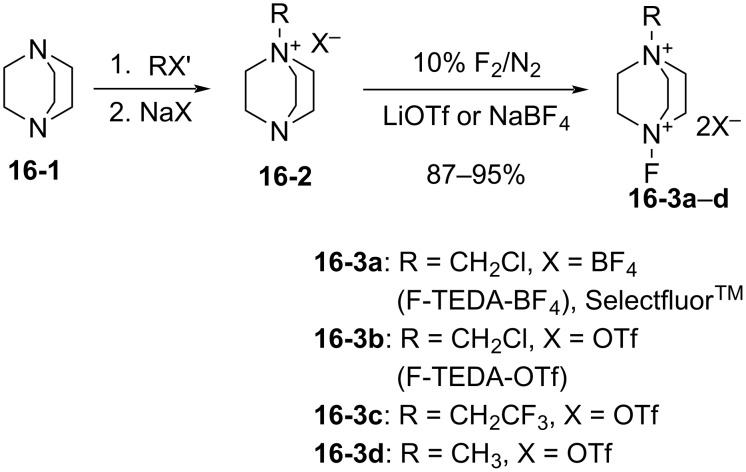
Synthesis of *N*-F salts **16-3**.

In 1992, Banks et al. reported a new series of *N*-fluoro diquaternary ammonium salts, 1-alkyl-4-fluoro-1,4-diazoniabicylco[2.2.2]octane salts **16-3a–d** (Scheme 33) [42]. The salts **16-3** were synthesized in high yields after alkylation of 1,4-diazabicyclo[2.2.2]octane (**16-1**), followed by the Umemoto’s fluorination/counteranion replacement reaction of the resulting monoquaternary ammonium salts **16-2** with 10% F_2_/N_2_ in acetonitrile at −40 to −20 °C. The resultant salts **16-3** proved to be stable, non-hygroscopic, crystalline solids with high fluorinating power. The salt **16-3a** was chosen as the commercial reagent, Selectfluor^TM^, from a cost-effectiveness viewpoint.

**Scheme 34:**
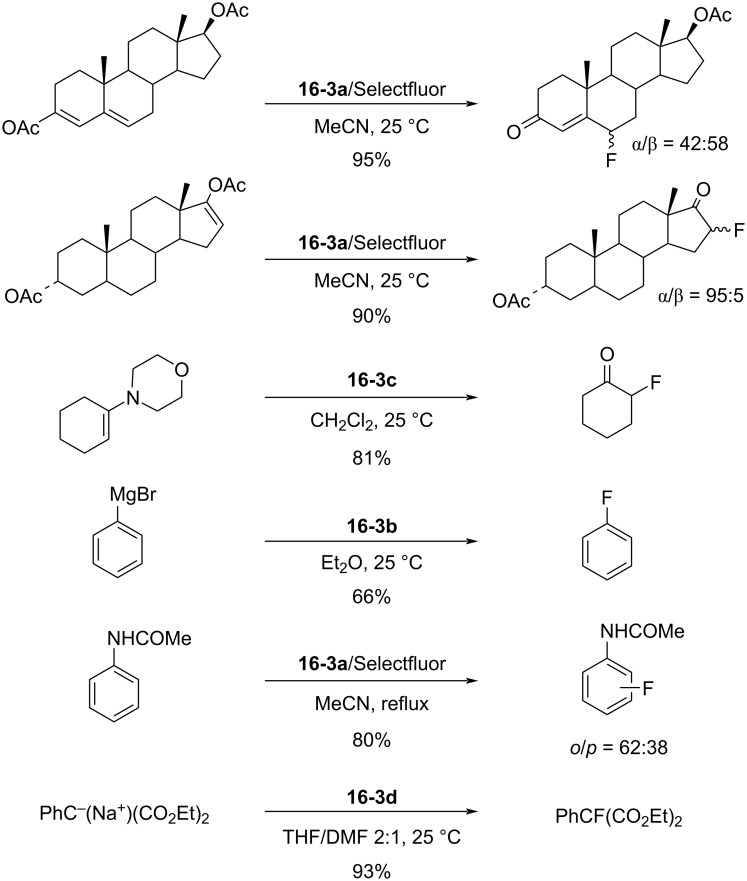
Fluorination with *N*-F salts **16-3**.

The fluorinating power of reagents **16-3** strengthened as the electronegativity of the R group increased in the order of CH_3_ < CH_2_Cl < CH_2_CF_3_. The reagents **16-3** were able to fluorinate enol and conjugated enol acetates of steroids, sodium malonates, enamines, Grignard reagents, and aromatic compounds under mild conditions. In each case Selectfluor gave the corresponding fluorinated products in good to high yields (Scheme 34).

**Figure 3:**
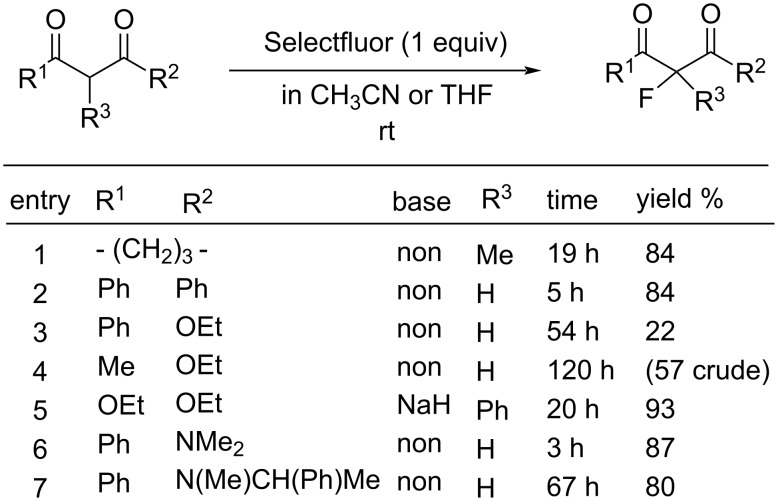
Monofluorination with Selectfluor (**16-3a**).

**Figure 4:**
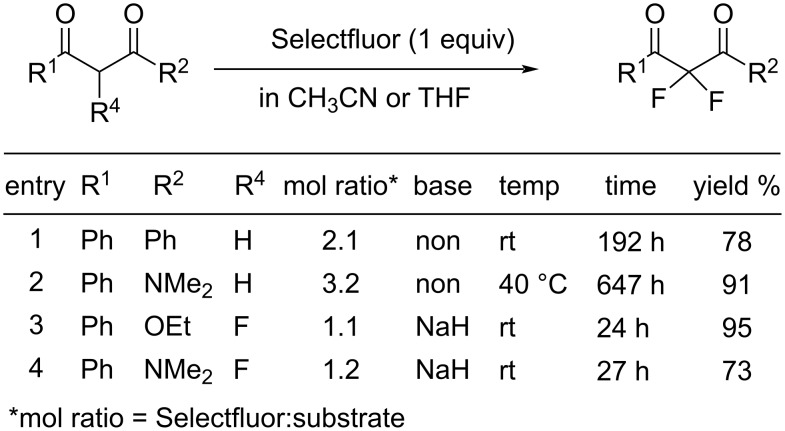
Difluorination with Selectfluor (**16-3a**).

As can be seen in Figure 3 and Figure 4, two years later (1994), Banks et al. reported mono- and difluorinations of various 1,3-dicarbonyl compounds using Selectfluor **(16-3a**) [61].

**Scheme 35:**
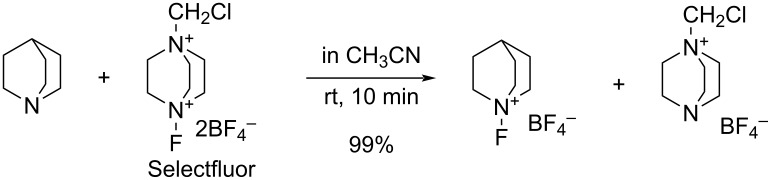
Transfer fluorination of Selectfluor (**16-3a**).

In 1995, the same group reported that Selectfluor reacted with quinuclidine to form *N*-fluoroquinuclidinium tetrafluoroborate in quantitative yield [62] (Scheme 35). They described this as a “transfer fluorination” since there was an intermolecular transfer of the fluorine atom of Selectfluor to the nitrogen of quinuclidine. In 1996, full details were published on the reactivities of all **16-3** reagents and the syntheses of **16-3** and intermediates **16-2** including additionally C_2_H_5_ and C_8_H_17_ as R group and PF_6_^−^ and FSO_3_^−^ as anion X^−^ [63–64].

**Scheme 36:**
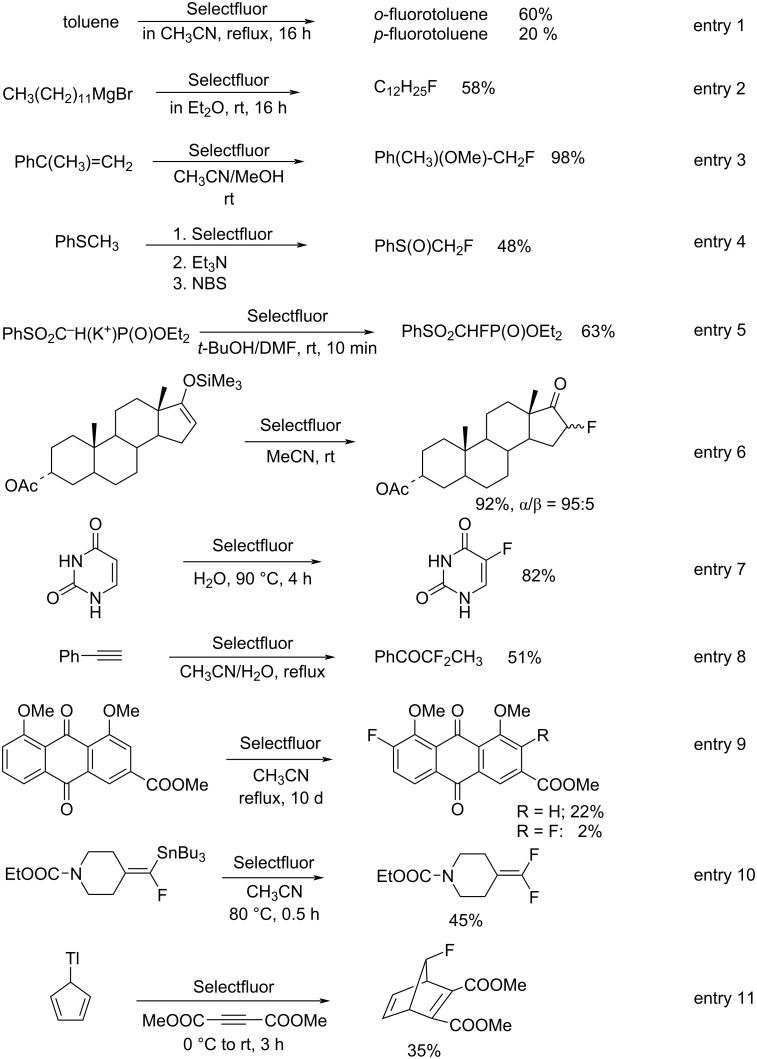
Fluorination of substrates with Selectfluor (**16-3a**).

The generous provision of free samples of Selectfluor after its commercialization resulted in many publications concerning its applications from a diversity of research groups and in a short time period (1992–1995): These included fluorinations of alkylbenzenes (entry 1, Scheme 36) [65], Grignard reagents (entry 2) [65], electron-rich alkenes (entry 3) [65–66], alkyl sulfides (entry 4) [65,67], 1,3-dicarbonyl compounds [65,68], phosphonate esters (entry 5) [65], steroidal silyl enol ethers and enol acetates (entry 6) [65], pyrimidine bases and nucleosides (entry 7) [67,69], phenylalkynes (entry 8) [70], anthraquinones (entry 9) [71], vinylstannanes (entry 10) [72], trialkylstannylindoles [73], and cyclopentadienylthallium (entry 11, Scheme 36) [74].

#### 1-17. Optically active *N*-fluoro-2,10-(3,3-dichlorocamphorsultam)

**Scheme 37:**
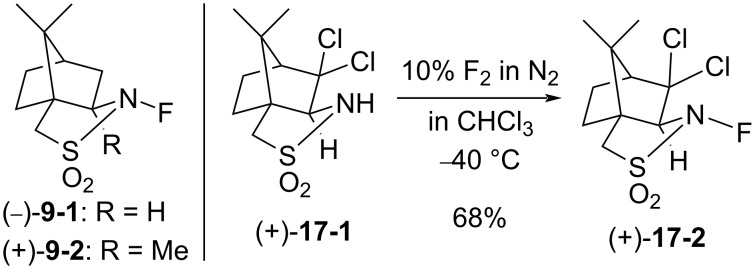
Synthesis of chiral *N*-fluoro-sultam **17-2**.

In 1993, Davis et al. reported the synthesis of the second optically active *N*-fluoro-2,10-(3,3-dichlorocamphorsultam) **17-2** [75] (Scheme 37).

**Scheme 38:**
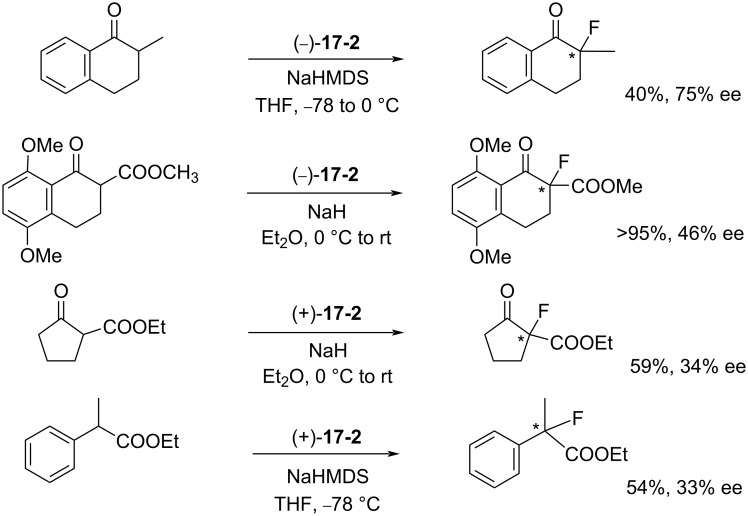
Asymmetric fluorination with chiral **17-2**.

The maximum enantioselectivity of enolates of β-ketoesters with (−)-**9-1** or **(+)-9-2**, first prepared by Lang in 1988 (see section 1-9)**,** was 70% ee. The asymmetric fluorination with (+)- or (−)-**17-2** afforded up to 75% ee as indicated in Scheme 38. The dichloro reagent **17-2** gave higher yields than the non-chloro reagent **9-1** because of the greater reactivity of **17-2**.

#### 1-18. Zwitterionic *N*-fluoropyridinium salts

**Figure 5:**
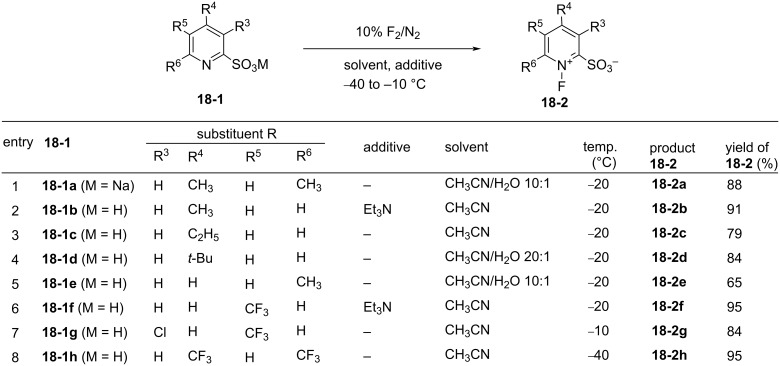
Synthesis of Zwitterionic *N*-fluoropyridinium salts **18-2a–h**.

In 1995, Umemoto and co-worker disclosed a zwitterionic *N*-fluoropyridinium salt system **18-2** which had a broad fluorinating power and high selectivity [76]. A series of *N*-fluoropyridinium-2-sulfonates with electron-withdrawing or -donating substituents were synthesized in high yields by fluorination of the corresponding pyridinium-2-sulfonates **18-1** with 10% F_2_/N_2_ in acetonitrile or a mixture of acetonitrile/water at −40 to −10 °C (Figure 5). In a few cases, a catalytic amount of triethylamine was used to improve the yields. The starting pyridinium-2-sulfonates possessing an electron-withdrawing group(s) were prepared in high yields from the reaction of the corresponding 2-chloropyridines with sodium sulfite. All of these *N*-F reagents are easy-to-handle and stable crystalline solids.

The nature of the lipophilic alkyl or trifluoromethyl substituents had a significant effect on the reactivity. Previously, *N*-fluoropyridinium-2-sulfonate and its 6-chloro derivative had been synthesized and shown to have excellent selectivity in fluorination reactions, but these reagents exhibited a low reactivity due to their low solubility in organic solvents [32].

**Scheme 39:**
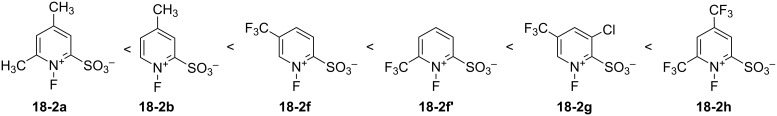
Fluorinating power order of zwitterionic *N*-fluoropyridinium salts.

Their fluorinating power increased in the order of **18-2a** < **2b** ≈ **2c** ≈ **2d** ≈ **2e** < **2f** < **2g** < **2h**, consistent with the order of the p*K*_a_ values of the pyridines (Scheme 39). The least powerful **18-2a** was suitable for the fluorination of reactive carbanions and easily oxidizable sulfides, whereas the most powerful **18-2h** was suitable for less-reactive substrates such as olefins, aromatics, and neutral active methylene compounds. *N*-Fluoro-6-(trifluoromethyl)pyridinium-2-sulfonate (**18-2f’**) was prepared later [77].

**Scheme 40:**
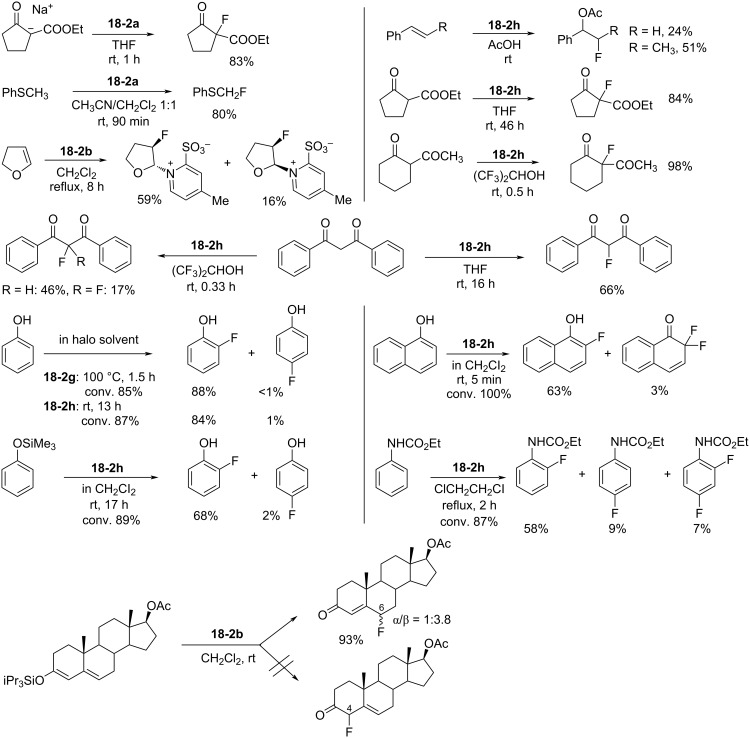
Fluorination with zwitterionic **18-2**.

As shown in Scheme 40, the **18-2** pyridinium series proved to be effective fluorinating agents for a wide range of substrates. Moreover, an extremely high *ortho*-selectivity was observed in the fluorination of phenol. This could be attributed to a hydrogen-bonding interaction between the sulfonate anion and the phenol hydroxy group in the transition state. This assumption could also explain the high *o*-selectivity obtained in the fluorinations of naphthol, phenylurethane, and trimethylsilyl ether of phenol, and the exclusive 6-selectivity observed in the fluorination of conjugated enol triisopropylsilyl ethers of steroids. An additional advantage of the **18-2** reagents is that, after the fluorinations, the resulting pyridine-sulfonic acids are easily removed in an aqueous work-up.

**Scheme 41:**
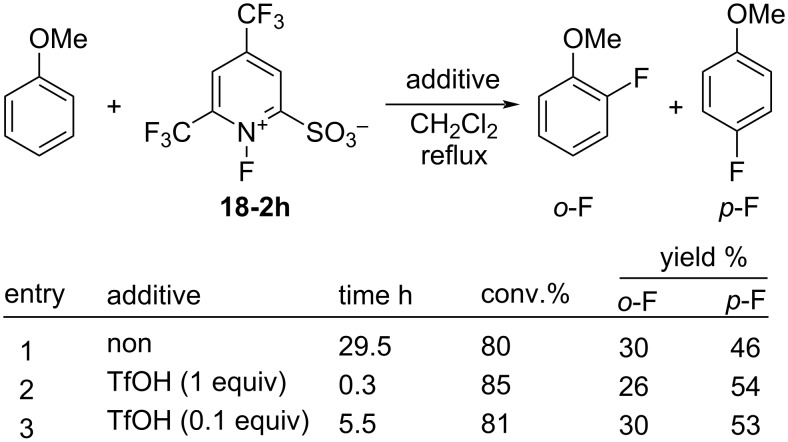
Activation of salt **18-2h** with TfOH.

As illustrated in Scheme 41, triflic acid was shown to promote the fluorination of anisole with **18-2h**. While the reaction time was ≈30 h without the additive (entry 1, Scheme 41), 1 equiv of TfOH shortened this to less than 1 h (entry 2). Therefore, it would appear that this additive acted as a catalyst. With 0.1 equiv of TfOH, the reaction time was 5.5 h (entry 3) which can be attributed to the strong electron-withdrawing effect of the 2-SO_3_H substituent formed by the protonation of the sulfonate anion in **18-2h**.

#### 1-19. 1-Fluoro-4-hydroxy-1,4-diazoniabicyclo[2.2.2]octane salts (NFTh)

**Scheme 42:**
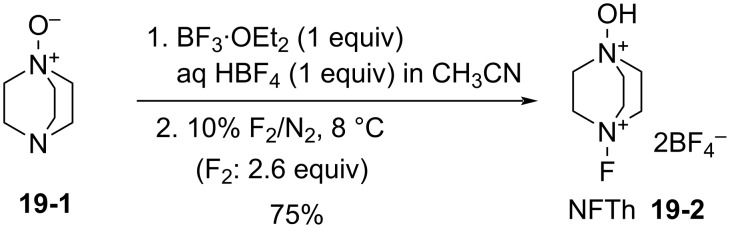
Synthesis of NFTh, **19-2**.

In 1995, Poss and Shia disclosed the synthesis and fluorination reactivity of 1-fluoro-4-hydroxy-1,4-diazoniabicyclo[2.2.2]octane bis(tetrafluoroborate) (**19**-**2**, NFTh/Accufluor^TM^) in a patent [78]. According to the patent, NFTh was prepared by fluorination of 1,4-diazabicyclo[2.2.2]octane *N*-oxide (**19-1**) in acetonitrile or water with 10% F_2_/N_2_ in the presence of HBF_4_, NaBF_4_, BF_3_ etherate, and/or BF_3_ at −35 °C or 8–9 °C. Scheme 42 shows the preparation of NFTh, a white solid that decomposes at 125 °C.

**Scheme 43:**
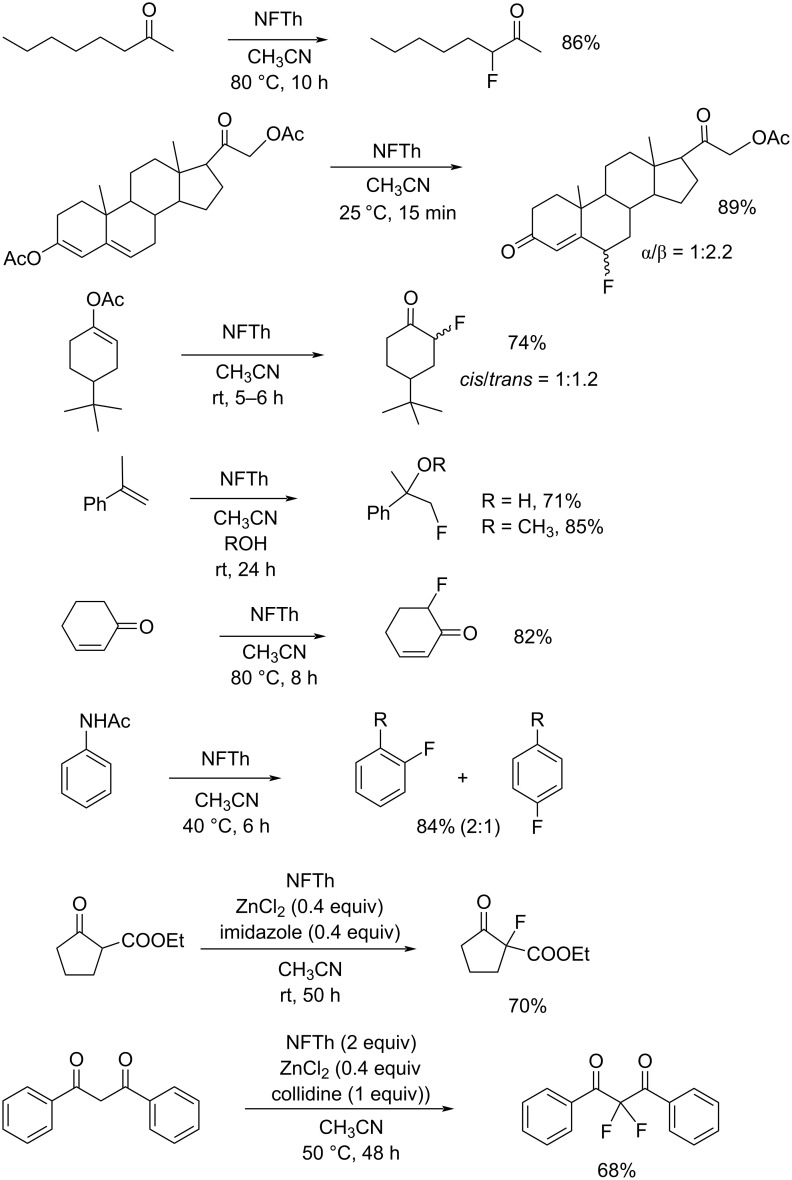
Fluorination with NFTh, **19-2**.

The fluorination scope of NFTh is shown in Scheme 43 [79–80]. NFTh reacted with aromatics, enols, ketones, activated olefins, and substrates with active methylene groups to produce the corresponding fluorinated products in good to high yields. NFTh is a powerful fluorinating agent comparable to Selectfluor. Since NFTh has an acidic proton, its effectiveness is curtailed in the case of anionic substrates.

#### 1-20. *N*-Fluorobenzo-oxathiazone dioxide

**Scheme 44:**
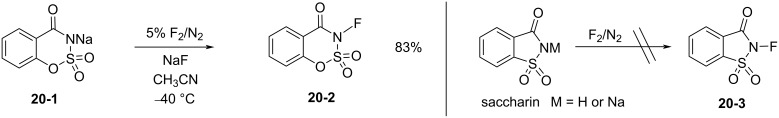
Synthesis of 3-fluorobenzo-1,2,3-oxathiazin-4-one 2,2-dioxide (**20-2**).

In 1995, Cabrera and co-worker claimed 3-fluorobenzo-1,2,3-oxathiazin-4-one 2,2-dioxide (**20-2**) to be a useful fluorinating agent; **20-2** is a stable crystalline compound that was prepared in 83% yield by fluorination of its precursor **20-1** with 5% F_2_/N_2_ in acetonitrile and in the presence of NaF at −40 °C [81] (Scheme 44). They also tried to prepare *N*-fluorosaccharin (**20-3**) from saccharin with the F_2_/N_2_ system, but failed. Reagent **20-3** was synthesized using cesium fluoroxysulfate in 1991 (see section 1-15).

**Scheme 45:**
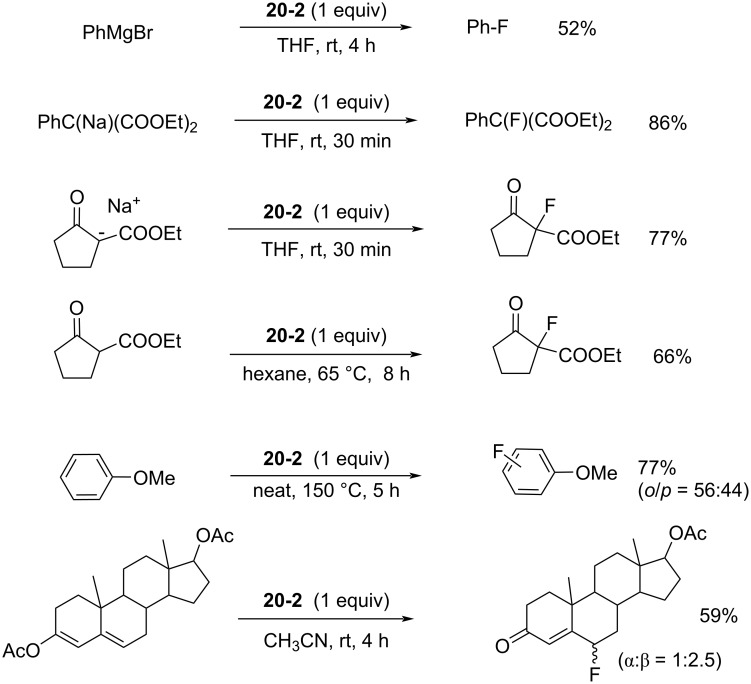
Fluorination with **20-2**.

Reagent **20-2** proved useful for the fluorination of both neutral and anionic nucleophiles under mild conditions. Scheme 45 illustrates some pertinent examples. Phenyl Grignard reagent, active methylene compounds, and a conjugated enol acetate of a steroid were all fluorinated in moderate to high yields. The fluorination of anisole required high temperature and neat conditions suggesting that the fluorination power of **20-2** is not so high.

#### 1-21. Perfluoro[*N*-fluoro-*N*-(4-pyridyl)acetamide]

**Scheme 46:**
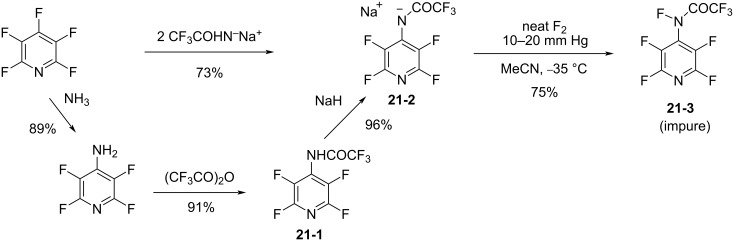
Synthesis of *N*-F amide **21-3**.

In 1996, the Banks group reported perfluoro[*N*-fluoro-*N*-(4-pyridyl)acetamide] (**21-3**) as a carboxamide analogue of perfluoro[*N*-fluoro-*N*-(4-pyridyl)methanesulfonamide] (**11-2**, see section 1-11) [82]. Its precursor, **21-2**, was prepared from pentafluoropyridine by either one of two methods (Scheme 46). Precursor **21-2** was treated with neat F_2_ at 10–20 mmHg pressure in acetonitrile at −35 °C to produce the *N*-F carboxamide **21-3** in 75% yield but the resulting product was a 79:18 mixture of the desired *N*-F product **21-3** and the protonated compound **21-1**.

**Scheme 47:**
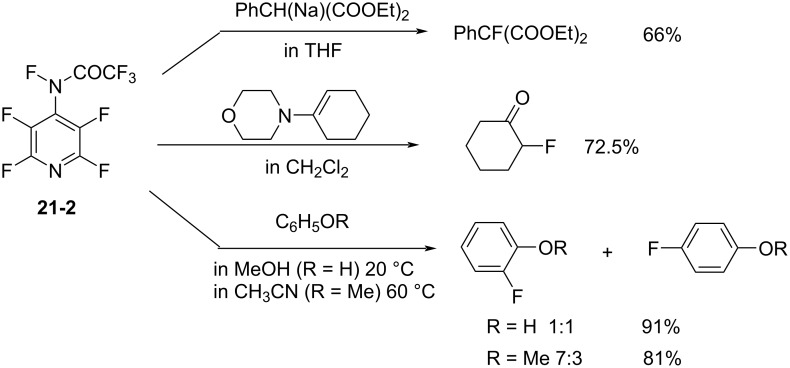
Fluorination with *N*-F amide **21-2**.

As a reagent *N*-F carboxamide **21-2** fluorinated electron-rich substrates such as sodium diethyl (phenyl)malonate, 1-morpholinocyclohexene, phenol, and anisole (Scheme 47). The fluorination power of the carboxamide **21-2** was less than that of its *N*-F sulfonamide analog **11-2**.

#### 1-22. *N,N’*-Difluoro-1,4-diazoniabicyclo[2.2.2]octane salts

**Scheme 48:**
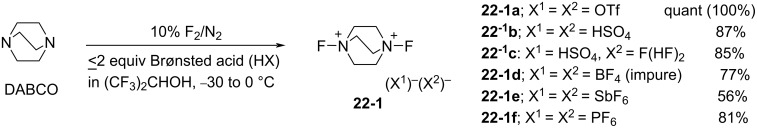
Synthesis of *N,N’*-difluorodiazoniabicyclo[2.2.2]octane salts **22-1**.

In 1996, Umemoto and co-worker reported the successful synthesis of various *N,N’*-difluoro-1,4-diazoniabicyclo[2.2.2]octane salts **22-1a**–**f** in a pure form and in good to excellent yields (Scheme 48) [83]. All the salts were fully identified by elemental analysis and spectral analysis. Previously, in 1992, Banks and co-workers described how attempts to synthesize **22-1** proved to be unsatisfactory [42] and in 1995, they reported their synthesis characterized only by ^19^F NMR and described that the salts were moisture-sensitive [84].

The synthesis is sensitive to solvents and the amount of Brønsted acids (HX). *N,N’*-Difluoro-1,4-diazoniabicyclo[2.2.2]octane bistriflate (**22**-**1a**), bis(hydrogensulfate) **22-1b**, hydrogensulfate bishydrogenfluoride fluoride **22-1c**, bishexafluoroantimonate **22-1e**, and bishexafluorophosphate **22-1f** were prepared in a pure form by fluorination of 1,4-diazabicyclo[2.2.2]octane (DABCO) with 10% F_2_/N_2_ in hexafluoroisopropanol in the presence of two molar equivalents or fewer of HX (one-step method, Scheme 48). The bisSbF_6_ salt **22-1e** and bisPF_6_ **22-1f** could also be prepared in a pure form by using acetonitrile as the solvent. However, bisBF_4_ salt **22-1d** could not be satisfactorily synthesized by this one-step protocol because an inseparable mixture of **22-1d** and the DABCO·2HBF_4_ salt formed.

**Scheme 49:**
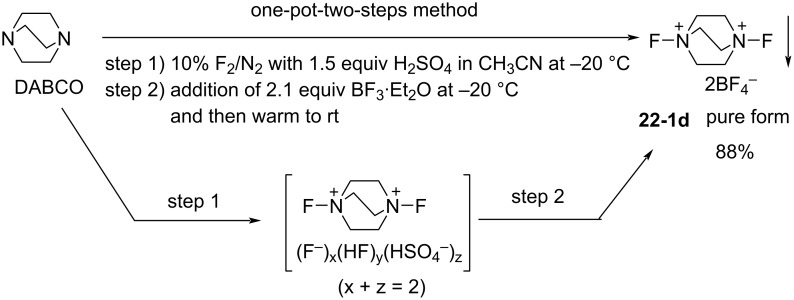
One-pot synthesis of *N,N’*-difluoro-1,4-diazoniabicyclo[2.2.2]octane bistetrafluoroborate salt (**22-1d**).

BisBF_4_ **22-1d** was considered to be commercially valuable because of its low preparative cost. Umemoto and co-worker found a clean counteranion replacement reaction of the intermediate (F^−^)_x_(HF)_y_(HSO_4_^−^)_z_ salts (x + z = 2) by using BF_3_ etherate and thus solved the problem with a simple two-step one-pot method; (step 1) fluorination of a mixture of DABCO and 1.5 molar equivalents of H_2_SO_4_ with 10% F_2_/N_2_ in acetonitrile at −20 °C, and (step 2) treatment of the resulting reaction mixture with 2.1 molar equivalents of BF_3_ etherate at −20 °C to room temperature, gave pure **22-1d** as a white precipitate (Scheme 49). By applying this telescoped method, pure **22-1d** was easily obtained in 88% yield only after simple filtration of the reaction mixture and no further purification was needed. Pure **22-1d** is non-hygroscopic and forms a stable crystalline solid with a high decomposition point of ca. 170 °C.

**Figure 6:**
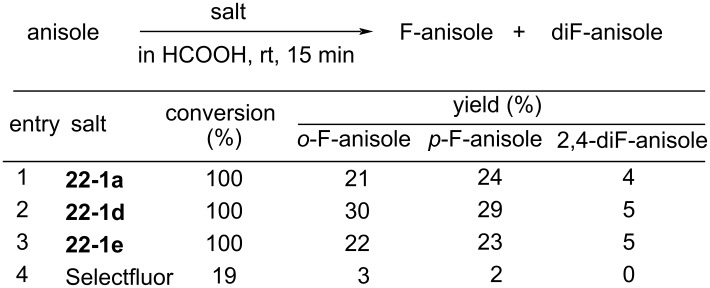
Fluorination of anisole with **22-1a**, **d**, **e**.

The bisOTf salt **22-1a**, bisBF_4_ **22**-**1d**, bisSbF_6_ **22**-**1e**, and bisPF_6_ **22**-**1f** are easy-to-handle because they are non-hygroscopic and stable crystals. As shown in Figure 6, **22-1a**,**d**,**e** mediated a quantitative conversion of anisole to isomers of fluoroanisole at room temperature after 15 min, whereas Selectfluor (**16-3a**) only produced fluoroanisole with a 19% conversion. This demonstrated that **22-1** is a much more powerful reagent than Selectfluor, a power that is attributed to the high electronegativity of the fluorine atom compared to the ClCH_2_ group.

**Scheme 50:**
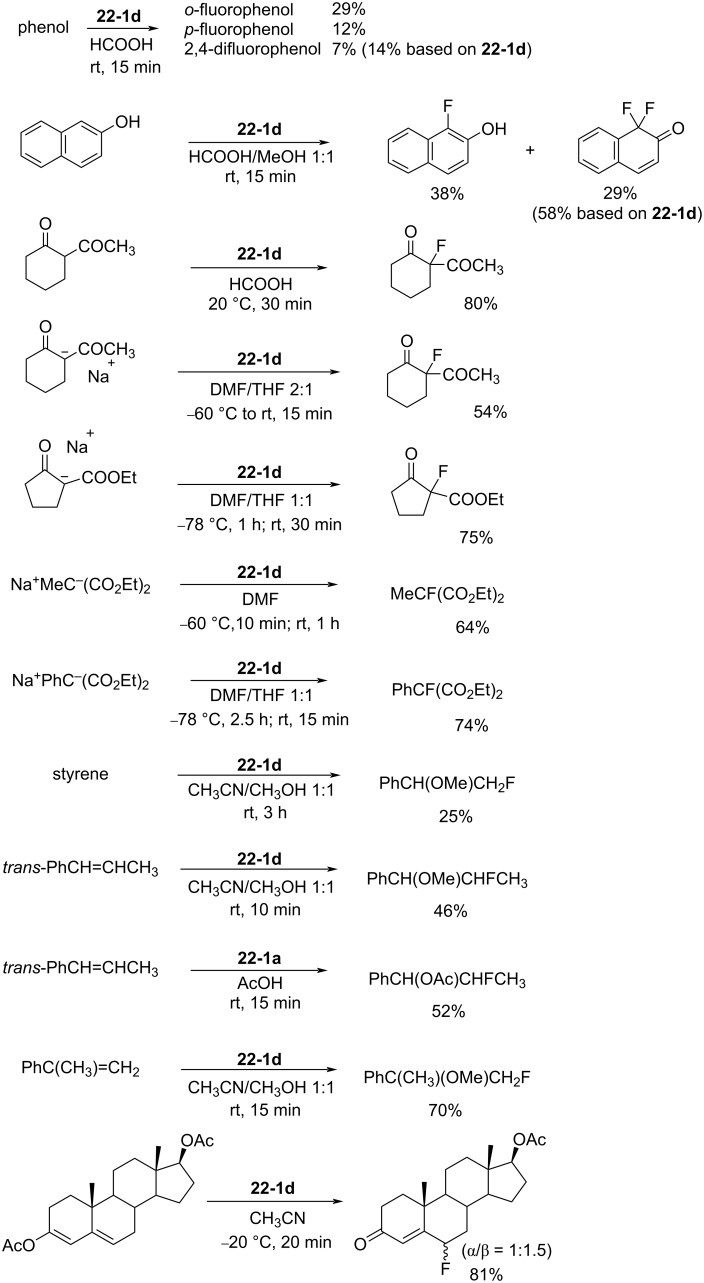
Fluorination with *N,N’*-diF bisBF_4_ **22-1d**.

By using the bistetrafluoroborate salt **22-1d**, many substrates such as aromatics, active methylene compounds and their salts, olefins, and enol acetates were efficiently fluorinated (Scheme 50). It was shown also that only one of the two N–F groups of **22-1** is used for *C*-fluorination, while the other N–F plays an activating role.

#### 1-23. Bis-*N*-fluoro reagents derived from precursors containing two heterocycles

**Scheme 51:**
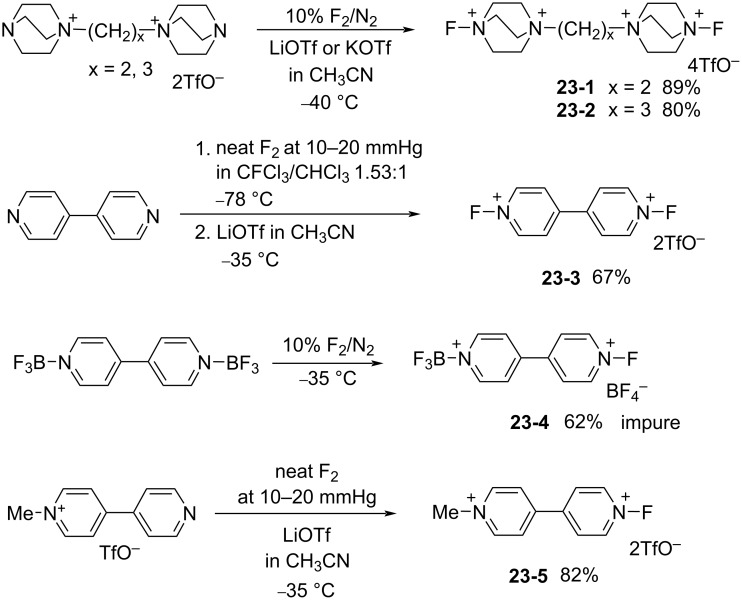
Synthesis of bis-N-F reagents **23-1**–**5**.

**Scheme 52:**
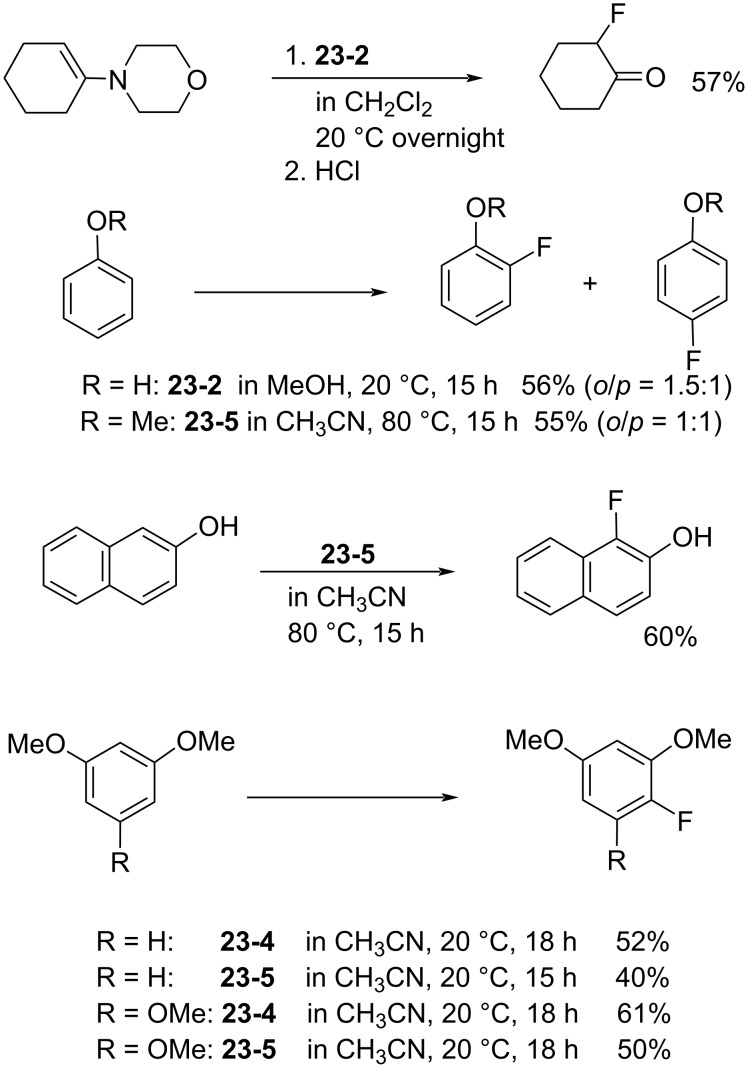
Fluorination with **23-2**, **4**, **5**.

In 1997, the Banks group reported bis-*N*-fluoro reagents **23-1**–**3** and related salts **23-4**,**5** by the fluorination of precursors containing two heterocycles (Scheme 51) [85]. Reagents **23-1–3** and **-5** were obtained in good yields and **23-4** was also obtained, but as an impure product. The reagents **23**-**1** and **23**-**2** are bis-Selectfluor-type reagents and **23**-**3** is a bis-*N*-fluoropyridinium reagent. The fluorinating ability of reagents **23-2**, -**4**, and -**5** were examined as shown in Scheme 52.

#### 1-24. *N,N’*-Difluorobipyridinium salts

**Figure 7:**
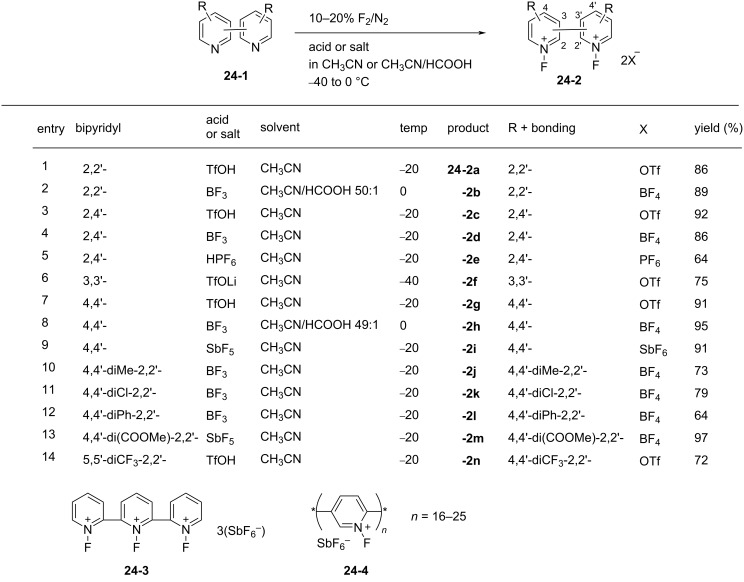
Synthesis of *N,N’*-difluorobipyridinium salts **24-2**.

In 1998, the Umemoto group reported a new series of *N,N’*-difluorobipyridinium salts **24-2**, *N,N’*-difluoro-2,2’-, -2,4’-, -3,3’-, and -4,4’-bipyridinium salts. These were synthesized by the direct fluorination of bipyridines **24-1** with 10–20% F_2_/N_2_ in the presence of a Lewis acid, a Brønsted acid, or its metal salt in acetonitrile or as a 50:1 mixture of acetonitrile/formic acid at −40 to 0 °C. The yields were good to excellent [86] (Figure 7). The trimer **24-3** and polymer homologues **24-4** were also prepared. The *N,N’*-difluorobipyridinium salts are stable and generally furnished non-hygroscopic free flowing materials, however, this was less the case for those derivatives with electron-withdrawing substituents.

**Figure 8:**
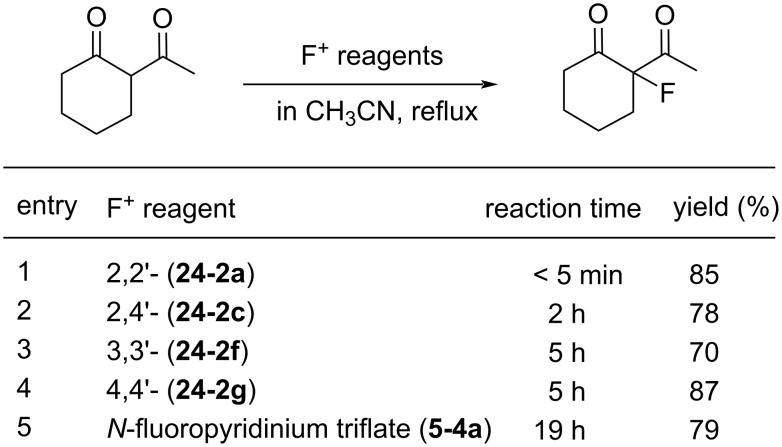
Controlled fluorination of *N,N’*-diF **24-2**.

**Scheme 53:**
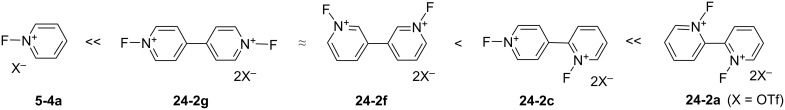
Fluorinating power of *N,N’*-diF salts **24-2** and *N*-F salt **5-4a**.

Controlled fluorination reactions with these reagents (Figure 8) clearly showed a power order of 2,2’- (**24-2a**) >> 2,4’- (**24-2c**) > 3,3’- (**24-2f**) ≈ 4,4’- (**24-2g**) >> *N*-fluoropyridinium triflate (Scheme 53), which is again in good agreement with the p*K*_a_ values of the bipyridines. The 2,2’-isomers had the highest power (reactivity) among the dipyridinium isomers, and they were much more reactive than the monomeric *N*-fluoropyridinium triflate (**5-4a**).

**Scheme 54:**
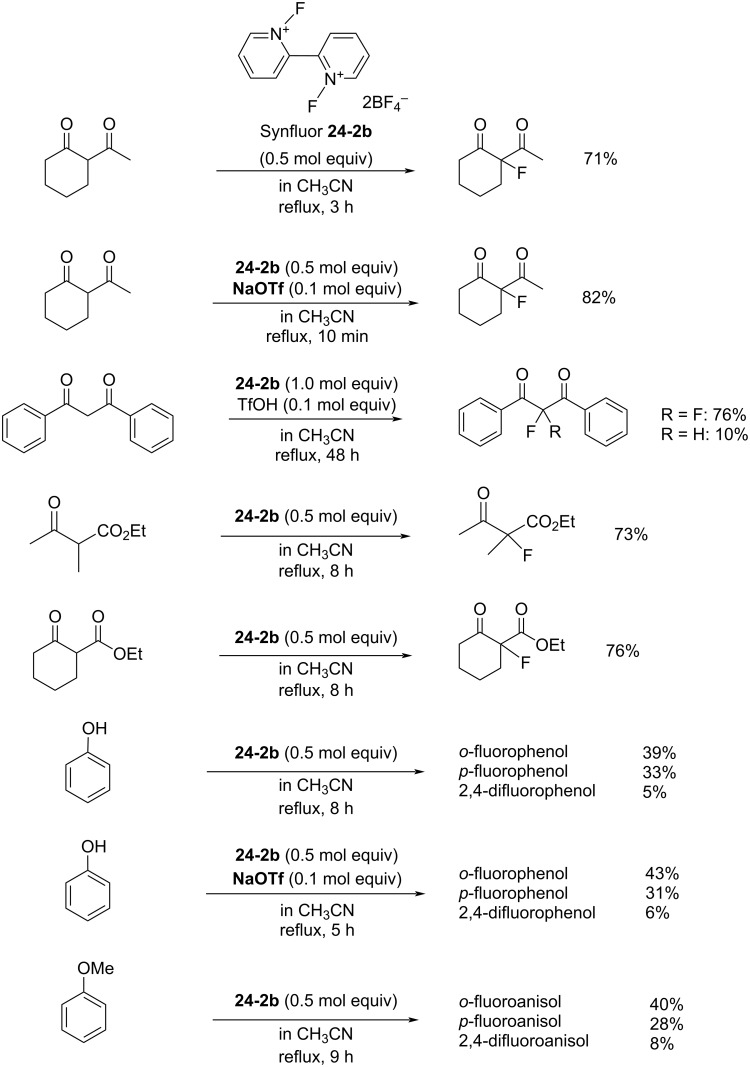
Fluorination reactions with Synfluor^TM^ (**24-2b**).

**Scheme 55:**
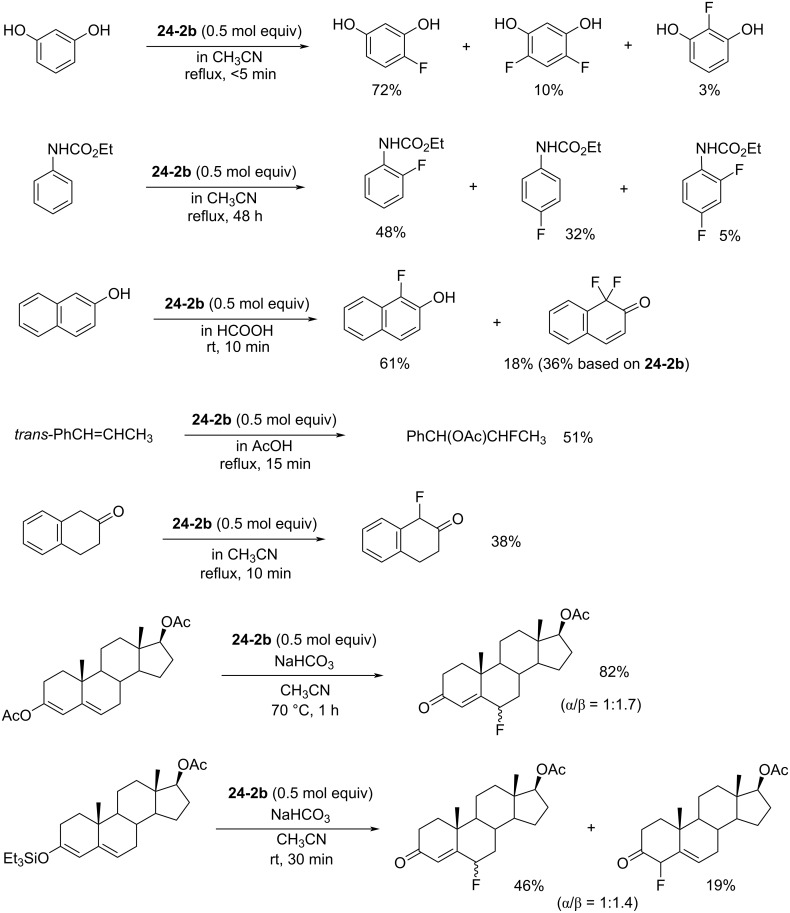
Additional fluorination reactions with Synfluor^TM^ (**24-2b**).

In contrast to the *N,N’*-difluoro-1,4-diazoniabicyclo[2.2.2]octane salts **22-1**, both of the *N*-F moieties in **24-2** were effective in fluorinations and they reacted sequentially. *N,N’*-Difluoro-2,2’-bipyridinium bis(tetrafluoroborate) (**24-2b**, X = BF_4_, SynFluor^TM^, MEC-31) was chosen as a representative of this class to assess fluorination capability because of its low production cost and high fluorine content per molecule. As a result, SynFluor^TM^ (**24-2b**) proved to be a practical reagent for the fluorination of many substrates affording the products in good to high yields (Scheme 54 and Scheme 55). SynFluor can be considered a powerful fluorinating agent with a high fluorine content, F: 103 g/1 kg (comparison; 54 g/1 kg for Selectfluor).

#### 1-25. *N*-Fluoro-2,4-dinitroimidazole

**Scheme 56:**
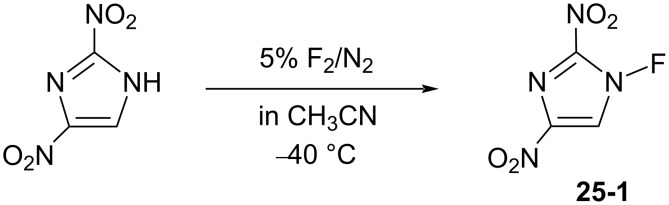
Synthesis of *N*-F **25-1**.

In 1998, Laali et al. reported the fluorination of polycyclic aromatic hydrocarbons (PAHs) with *N*-fluoro-2,4-dinitroimidazole (**25-1**) [87] (Scheme 56). This reagent is a white solid that was prepared by Forohar et al. through fluorination of 2,4-dinitroimidazole (5% F_2_ in N_2_ at −40 °C) [88]. Although additional information on **25-1** was not available, it was noticed [87] that the polynitro compound **25-1** is potentially dangerous towards detonation.

**Scheme 57:**
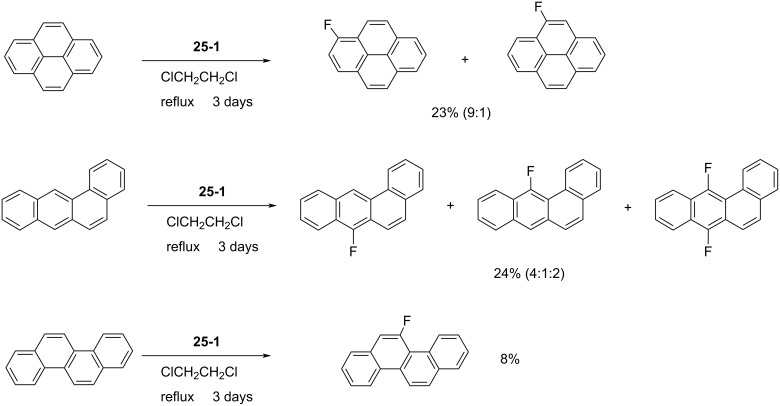
Fluorination of polycyclic aromatics with **25-1**.

Laali et al. attempted to fluorinate some polycyclic aromatics with Selectfluor, but intractable mixtures were obtained. Thus, they examined the utility of **25-1** in the fluorination of polycyclic aromatics. Although a detailed optimization was conducted, the fluorination of over 20 polycyclic aromatics was poor giving products in very low yields, varying from 27% to 3% depending on the substrate. The fluorinations were accompanied by tar formation and dimerization. Scheme 57 illustrates some representative examples.

#### 1-26. Perfluoro-(*N*-fluoro-2,2,6,6-tetramethylpiperidine) and its 2,6-dimethyl analogue

**Scheme 58:**
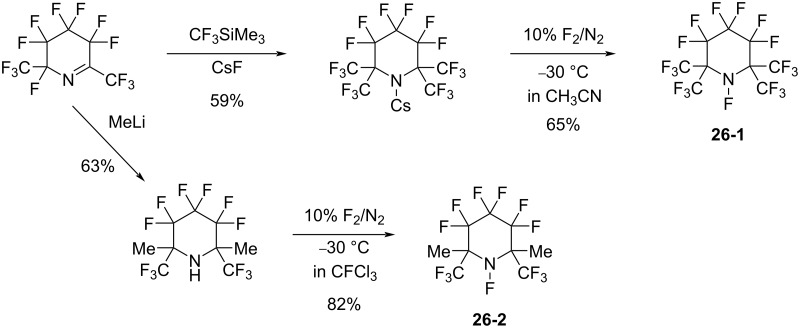
Synthesis of **26-1** and dimethyl analog **26-2**.

In 1999, the Banks group reported the syntheses of perfluoro-(*N*-fluoro-2,2,6,6-tetramethylpiperidine) (**26-1**) and its 2,6-dimethyl analogue **26-2**, reagents which do not have α-fluorine atoms adjacent to the *N*-F site [89] (Scheme 58). These reagents overcame the drawback of perfluoro-*N*-fluoropiperidine (**1-1**) which does contain α-fluorine atoms (see section 1-1).

**Scheme 59:**
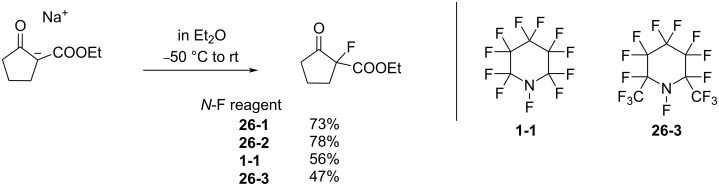
Fluorination with reagents **26-1**, **26-2**, **1-1**, and **26-3**.

The fluorinating ability of reagents **26-1** and -**2** was tested in fluorinations of the sodium salt of a keto ester (Scheme 59). Reagents **26-1** and -**2** were superior to **1-1** and **26-3** where the α-fluorine atoms can be eliminated after fluorine-transfer from the *N*-F site.

#### 1-27. *N*-Fluoro-3-ethyl-3-methyldioxobenzothiazinone and (*R*)- and (*S*)-*N*-fluoro-3-cyclohexyl-3-methylbenzoisothiazole dioxide

**Scheme 60:**
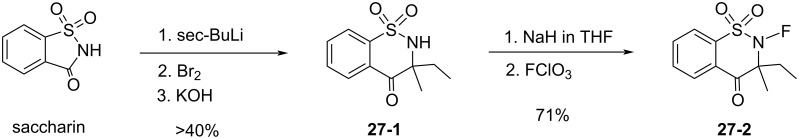
Synthesis of *N*-F reagent **27-2**.

In 1999, Takeuchi et al. reported *N*-fluoro-3-ethyl-3-methyl-1,1-dioxo-2,3-dihydro-1*H*-1λ^6^-benzo[*e*]1,2-thiazin-4-one (**27-2**) as an efficient reagent for the fluorination of carbanions [90]. The precursor **27-1**, which was prepared in a three step protocol from saccharin, was fluorinated with FClO_3_ to give **27-2** in good yield (Scheme 60). Direct fluorination of **27-1** with 10% F_2_/N_2_ had failed because of decomposition. Various ketone enolates were successfully fluorinated with reagent **27-2** in good to high yields [90].

**Scheme 61:**
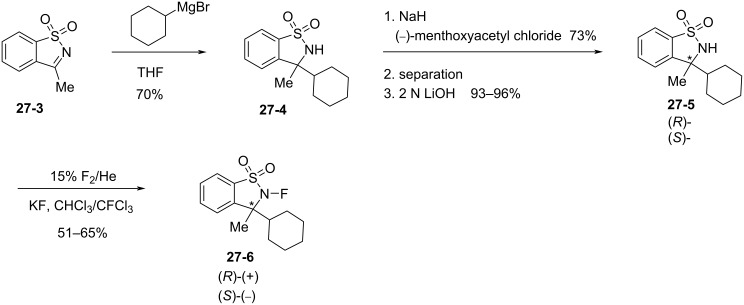
Synthesis of chiral *N*-F reagents **27-6**.

In addition, Takeuchi et al. reported that optically active *N*-fluorosultams, (*R*)- and (*S*)-*N*-fluoro-3-cyclohexyl-3-methyl-2,3-dihydrobenzo[1,2-*d*]isothiazole 1,1-dioxides **27-6** (Scheme 61) were efficient reagents for the asymmetric fluorination of enolates [91].

**Scheme 62:**
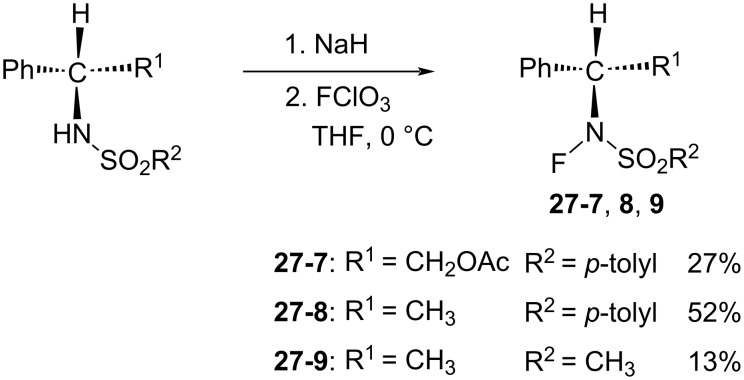
Synthesis of chiral *N*-F **27-7**–**9**.

To showcase his methodology, they first synthesized acyclic optically active *N*-fluoro sulfonamides **27-7**–**9** (Scheme 62) and attempted the enantioselective fluorination of some enolates in 1997 [92]. However, the best result was an enantiomeric excess of 48% with a chemical yield of 53% after the enolate anion of 2-benzyl-α-tetralone was treated with **27-8**.

As summarized in Scheme 61, Takeuchi et al. prepared the enantiomers of cyclic *N*-F sulfonamide **27-6** by the approach used by Lang for *N*-fluorosultams [53]. A cyclohexyl ring was introduced nucleophilically into imine **27-3** and the resulting **27-4** was treated with (−)-menthoxyacetyl chloride, followed by separation of the diastereomers. The chiral auxiliary was then removed with LiOH and the resulting sultams **27-5** as single enantiomers were fluorinated with 15% F_2_/He in the presence of KF to produce optically pure *N*-fluorosultam reagents (*R*)- and (*S*)-**27-6**.

**Scheme 63:**
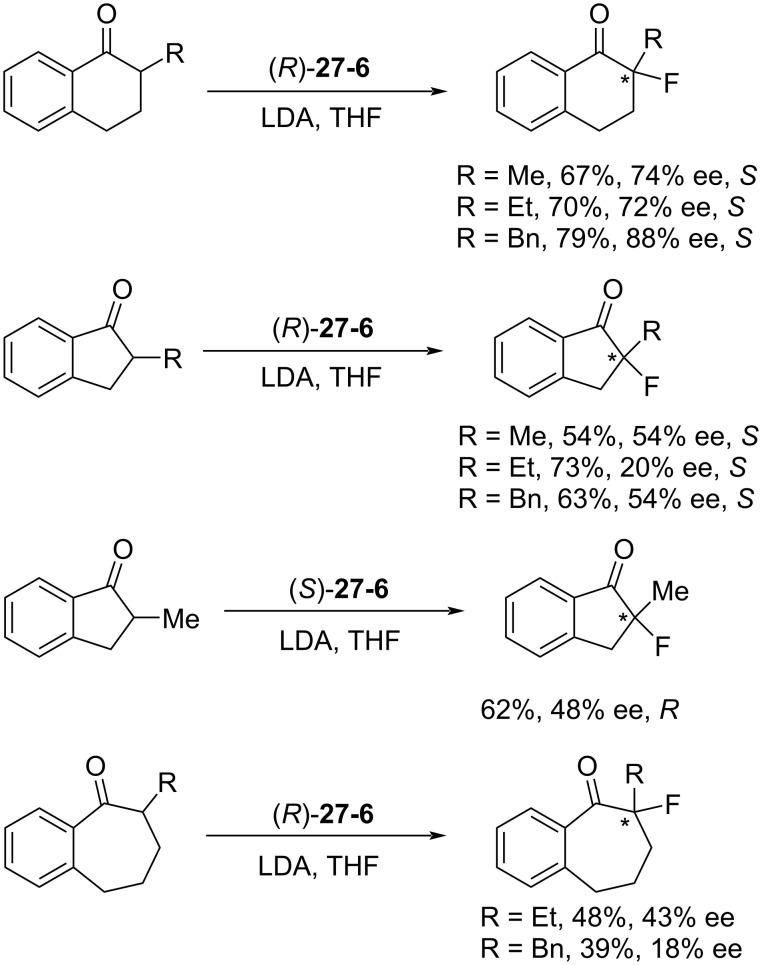
Asymmetric fluorination with **27-6**.

Enantioselective fluorinations of typical enolates were then performed (Scheme 63). The (*R*)-**27-6** reagent gave up to 79% yield and 88% enantiomeric excess in the case of 2-benzyl-α-tetralone.

#### 1-28. (*R*)- and (*S*)-*N*-fluoro-3-*tert*-butyl-7-nitro-benzothiazine 1,1-dioxides and spiro-type analogues

**Scheme 64:**
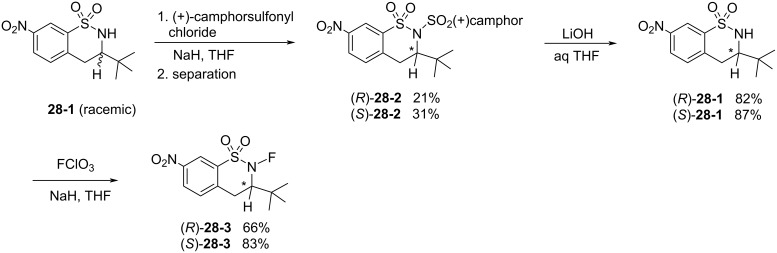
Synthesis of chiral *N*-F reagents **28-3**.

In 2000, the Takeuchi group reported their second series of chiral *N*-fluorosultam reagents, (*R*)- and (*S*)-*N*-fluoro-3-*tert*-butyl-7-nitro-3,4-dihydro-2*H*-benzo[*e*][1,2]thiazine 1,1-dioxides **28-3** [93] (Scheme 64). Racemic **28-1** reacted with (+)-10-camphorsulfonyl chloride to give a mixture of the diastereomers of **28-2**, which was accompanied by 45% of unreacted **28-1**. Interestingly, pure (*R*)-isomer **28-1** was obtained in 20% yield from the unreacted **28-1**. The diastereomer separation of **28-2** was achieved by column chromatography to separate the (*R*)- and (*S*)-isomers of **28-2** in 21 and 31% yields, respectively. Deprotection followed by fluorination with FClO_3_ gave (*R*)- and (*S*)-**28-3** in good yields. X-ray crystallography was used to determine the structure and confirm the absolute stereochemistry of (*S*)-**28-3**.

**Scheme 65:**
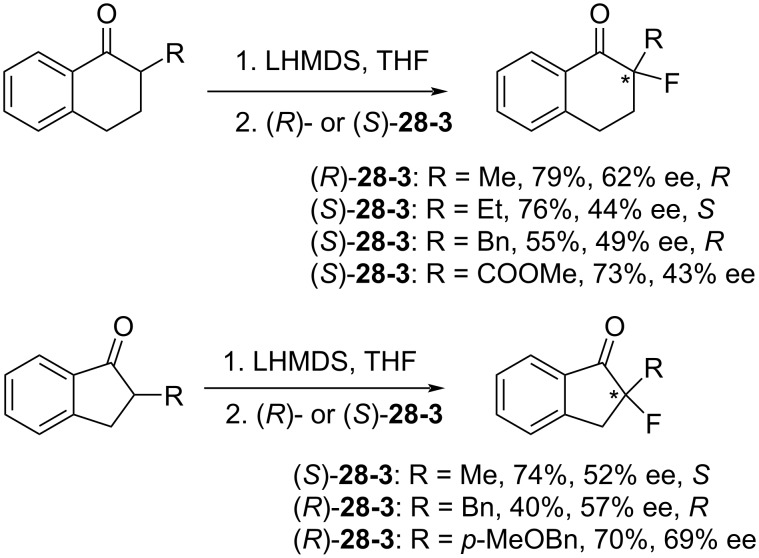
Asymmetric fluorination with **28-3**.

The optically active reagents (*R*)- and (*S*)-**28-3** were effective in the enantioselective fluorination of cyclic ketones, as is illustrated with representative in Scheme 65.

**Scheme 66:**
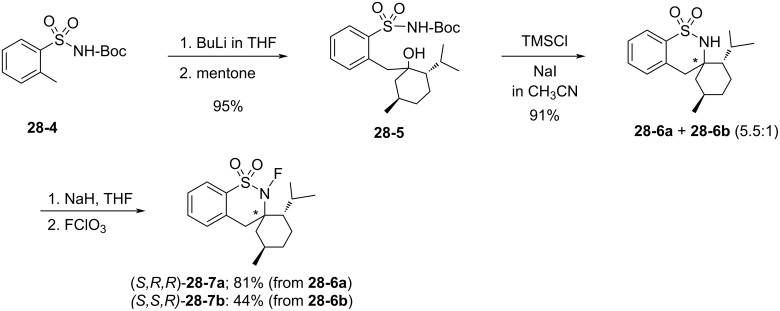
Synthesis of chiral *N*-F reagents **28-7**.

In addition, the Takeuchi group reported a spiro-type analogue, (2'*S*,3*R*,5'*R*)-2-fluoro-2'-methylethyl-5'-methyl-2*H*,4*H*-spiro[benzo[*e*][1,2]thiazine-3,1'-cyclohexane]-1,1-dione (**28-7a**) [94], as their third chiral reagent in this sultam series. As can be seen in Scheme 66, **28-4** was treated with BuLi followed by reaction with optically active menthone to give **28-5**, which was converted to enantiomers of spiro sultams **28-6a** and **28-6b** in high yield. The spiro sultams **28-6a** and **-6b** were separated and each was fluorinated with FClO_3_ to give (2'*S*,3*R*,5'*R*)-isomer **28-7a** and (2'*S*,3*S*,5'*R*)-isomer **28-7b** in 81% and 44% yield, respectively.

**Figure 9:**
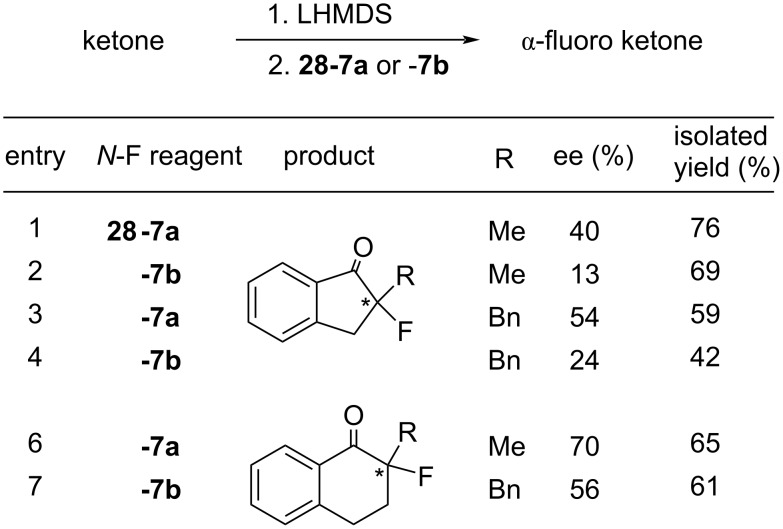
Asymmetric fluorination with **28-7**.

Figure 9 summarizes the outcomes of asymmetric fluorination reactions of enolates of aryl ketones using **28-7a** and **-7b**. In the event isomer **28-7a** yielded much better ees than **28-7b**. Although **28-7a** gave a maximum 70% ee (entry 6, Figure 9), it was less than that obtained (74% ee) by *N*-fluorosultam (*R*)-**27-6** with the same substrate (see the previous section 1-27).

#### 1-29. *N*-Fluorinated cinchona alkaloid derivatives by combination with Selectfluor

**Scheme 67:**
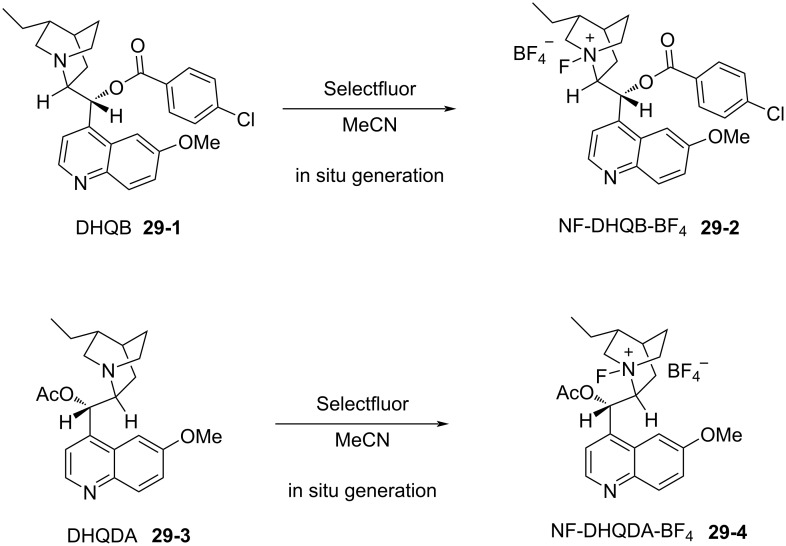
In situ formation of *N*-fluorinated cinchona alkaloids with Selectfluor^TM^.

In 2000, Shibata and Takeuchi reported a far more practical enantioselective fluorination method. They discovered that the fluorination of carbanions with Selectfluor occurred in a highly enantioselective manner when carried out in the presence of cinchona alkaloid derivatives [95]. This method consisted of two simple steps. Firstly, the cinchona alkaloid is reacted with Selectfluor in acetonitrile at room temperature for 1 h, and then this is followed by addition of the substrate. The resulting mixture is stirred at a suitable temperature. They proposed that Selectfluor transfers fluorine to the alkaloid to give a chiral *N*-F alkaloid species, in a manner that followed the fluorine transfer reported by Banks when quinuclidine was *N*-fluorinated with Selectfluor [62] (Scheme 67). Subsequently, in 2001, Shibata et al. presented full details of their studies including the definitive identification of *N*-fluorinated cinchona alkaloids by X-ray crystallography analysis and further applications [96]. This method proved to be far more practical than the enantiomeric sulfonamide-type *N*-F reagents developed until then.

**Scheme 68:**
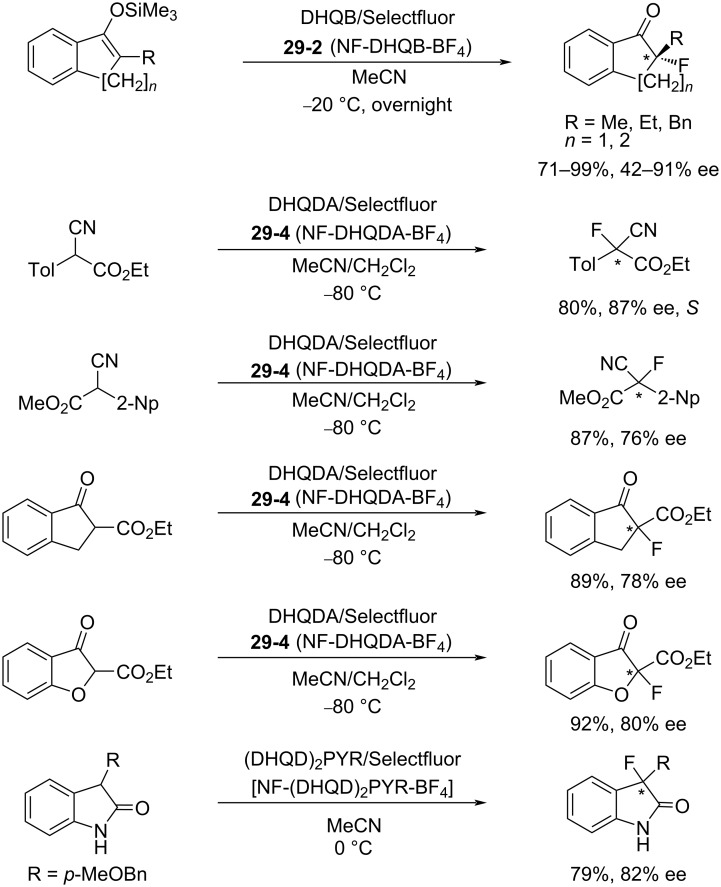
Asymmetric fluorination with *N*-F alkaloids formed in situ.

Many cinchona alkaloid derivatives were tested for their ability to fluorinate substrates such as cyclic and acyclic ketones and esters. Scheme 67 shows two typical examples of *N*-F cinchona alkaloid species, **29-2** and **29-4**, formed from dihydroquinine 4-chlorobenzoate (DHQB, **29-1**) and dihydroquinidine acetate (DHQDA, **29-3**) with Selectfluor. As a result, high chemical yields and high enantiomeric excesses were obtained for many substrates as summarized in Scheme 68. The DHQB/Selectfluor combination was effective for the fluorination of silyl enol ethers of indanones and tetralones, forming the fluorinated products in up to 91% ee. The DHQDA/Selectfluor combination was effective also for acyclic esters, with outcomes up to 87% ee, and for cyclic keto esters, up to 80% ee. For oxindoles, the (DHQD)_2_PYR/Selectfluor combination was effective too, generating the products with up to 82% ee.

#### 1-30. Enantiopure *N*-fluorocinchona alkaloid salts

**Scheme 69:**
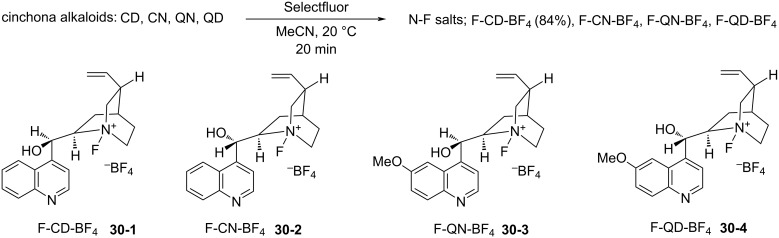
Synthesis of *N*-fluorocinchona alkaloids with Selectfluor.

Only six days after the report by Shibata and Takeuchi appeared (Oct. 2000), Cahard et al. reported the synthesis and application of similar enantiopure *N*-fluoro salts of cinchona alkaloids through the reaction of the alkaloids with Selectfluor [97]. Following the precedent from Banks’s fluorine-transfer reaction from Selectfluor to the *N*-site of quinuclidine [62], Cahard et al. isolated four *N*-fluorocinchona alkaloid salts, F-CD-BF_4_ **30-1**, F-CN-BF_4_ **30-2**, F-QN-BF_4_ **30-3**, and F-QD-BF_4_ **30-4** in good yields (Scheme 69). Soon after in 2001, they reported the X-ray structural analysis of F-CD-BF_4_ **30-1** [98].

**Scheme 70:**
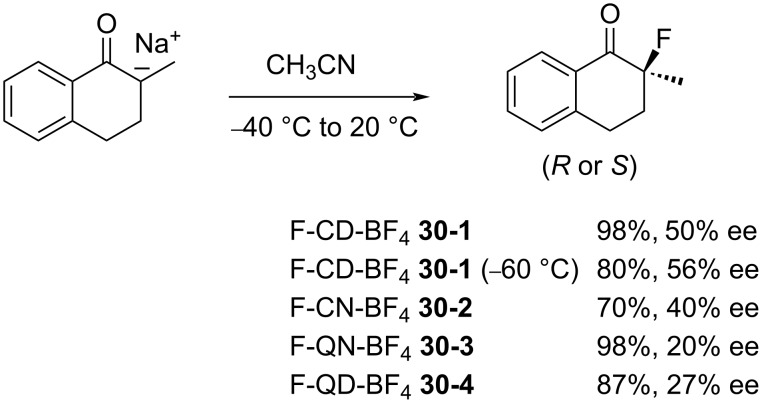
Asymmetric fluorination with **30-1**–**4**.

The enantioselective fluorination of the sodium enolate of 2-methyl-1-tetralone was examined using these *N*-F alkaloid salts (Scheme 70). F-CD-BF_4 _**30-1** gave the highest result with a 56% ee. F-CD-BF_4 _**30-1** is a nonhygroscopic, free-flowing solid with a high decomposition point (189 °C, dec).

Enantioselective fluorinations of the sodium enolates of other ketones and β-keto esters with F-CD-BF_4_ **30-1** were also investigated. The trimethylsilyl enol ether of 2-methyl-1-tetralone was examined, too (up to 61%ee, 93% yield). These experiments gave the fluorinated products in excellent chemical yields but with lower enantiomeric excesses than the Shibata and Takeuchi’s protocol.

#### 1-31. *N*-Fluoro-*p*-chlorobenzoylquinine salt: another *N*-F reagent of cinchona alkaloid

**Scheme 71:**
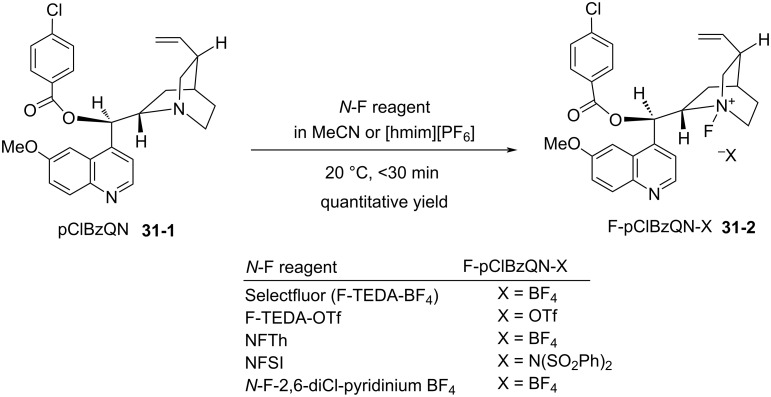
Transfer fluorination from various *N*-F reagents.

In 2003, Cahard et al. reported the transfer fluorination of a cinchona alkaloid, *p*-chlorobenzoylquinine (pClBzQN, **31-1**) using not only Selectfluor^TM^ (F-TEDA-BF_4_) but also other powerful *N*-F fluorinating agents such as 1-fluoro-4-hydroxy-1,4-diazoniabicyclo[2.2.2]octane bistetrafluoroborate (NFTh, **19-2**), *N*-fluorobenzenesulfonimide (NFSI), and *N*-fluoro-2,6-dichloropyridinium tetrafluoroborate (**5-4s**) (Scheme 71) [99]. These reactions produced F-pClBzQN-X **31-2** quantitatively at 20 °C within 30 min in acetonitrile or in an ionic liquid such as 1-hexyl-3-methylimidazolinium hexafluorophosphate [hmim][PF_6_]. However, the less powerful reagents, *N*-fluoroquinuclidinium tetrafluoroborate, *N*-fluoro-*N*-methyl-*p*-toluenesulfonamide (**4-1a**), *N*-fluoropyridinium triflate (**5-4a**), *N*-fluoro-2,4,6-trimethylpyridinium tetrafluoroborate (**5-4k**), and *N,N’*-difluoro-2,2’-bipyridinium bistetrafluoroborate (**24-2b**) failed. Among the five effective fluorine-transfer reagents, the 2,6-dichloropyridinium salt **5-4s** was more cost effective because it has a higher fluorine content (**5-4s**, 3.94 mmol/g) than the others (Selectfluor, 2.82 mmol/g; F-TEDA-OTf, 2.09 mmol/g; NFTh, 3.11 mmol/g; NFSI, 3.17 mmol/g).

**Figure 10:**
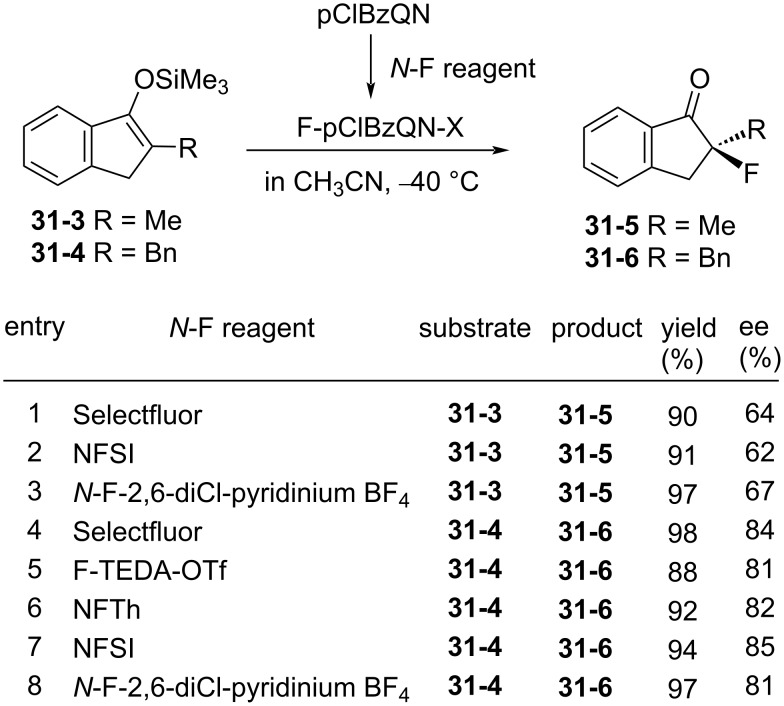
Asymmetric fluorination of silyl enol ethers.

The enantioselective fluorination of F-pClBzQN-X **31-2** was examined on trimethylsilyl enol ethers of methyl- and benzylindanones **31-3** and -**4** (Figure 10). High chemical yields and ees (88–97% and 81–85% ee) were obtained in the reactions of benzyl derivative **31-4** with F-pClBzQN-X [X = BF_4_ or N(SO_2_Ph)_2_] prepared in situ from the *N*-F reagents and pClBzQN (entries 4–8 in Figure 10).

#### 1-32. *N*-Fluoro-2,4,6-trichloro-1,3,5-triazinium tetrafluoroborate

In 2003, Banks et al. reported the synthesis of *N*-fluoro-2,4,6-trichloro-1,3,5-triazinium tetrafluoroborate (**32-2**) and its fluorination of aromatic compounds [100]. Although the syntheses of *N*-fluoro-2,4,6-trifluoro-1,3,5-triazinium hexafluoroarsenate and *N*-fluoro-2,4,6-trichloro-1,3,5-triazinium hexafluoroarsenate had been reported a decade earlier (1993) [101], their fluorination ability had not been disclosed.

**Scheme 72:**
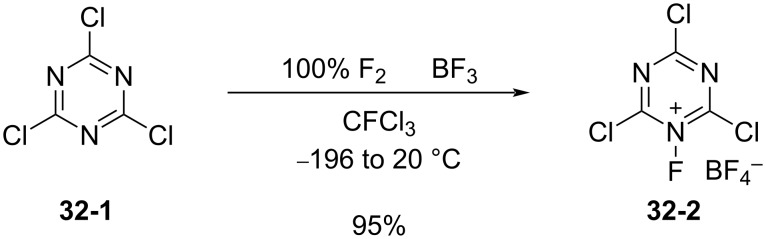
Synthesis of *N*-fluoro salt **32-2**.

Salt **32-2** was prepared in high yield in a small-scale batch reactor (Scheme 72). Accordingly, 100% F_2_ (1 equiv) and BF_3_ (1 equiv) gas were condensed into a stainless steel autoclave cooled at −196 °C, which contained cyanuric chloride (**32-1**, 1 equiv) in CFCl_3_. This was followed by gradual warming to room temperature and the reaction mixture was stored for 5 days. The resultant salt **32-2** is a moisture-sensitive white solid that decomposes at 153–155 °C.

**Scheme 73:**
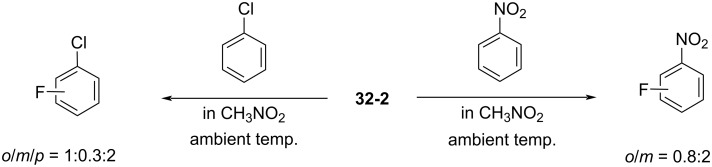
Reactivity of *N*-fluorotriazinium salt **32-2**.

Reagent **32-2** reacted with deactivated benzenes such as chlorobenzene and nitrobenzene at ambient temperature (Scheme 73). This outcome indicated that **32-2** was a more powerful fluorinating reagent than the *N*-fluoropentachloropyridinium salts **5-4v**,**w**. However, reagent **32-2** is not easy-to-handle because of its moisture-sensitivity.

#### 1-33. Bulky *N*-fluorobenzenesulfonimides (NFBSI)

**Scheme 74:**
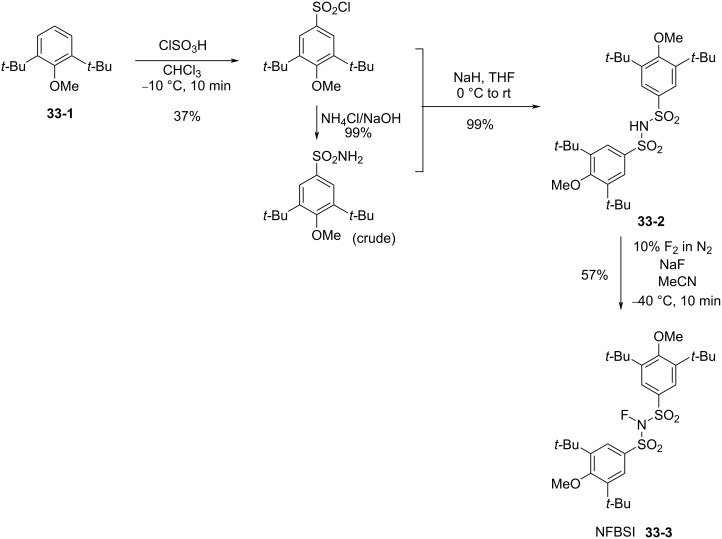
Synthesis of bulky *N*-fluorobenzenesulfonimide NFBSI **33-3**.

In 2011, Shibata et al. reported a bulky NFSI analog, *N*-fluoro-(3,5-di-*tert*-butyl-4-methoxy)benzenesulfonimide (NFBSI, **33-3**) [102]. This stable and crystalline reagent was synthesized in 57% yield by fluorination of bis(3,5-di-*tert*-butyl-4-methoxybenzenesulfonyl)amide (**33-2**) with 10% F_2_/N_2_ in the presence of NaF in acetonitrile at −40 °C (Scheme 74). X-ray crystal structure analysis revealed that the fluorine atom of NFBSI was surrounded by four bulky *tert*-butyl substituents.

**Scheme 75:**
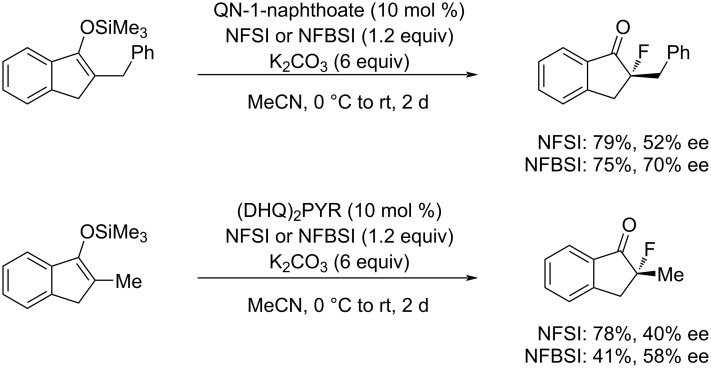
Comparison of NFSI and NFBSI.

This feature improved the enantioselectivity in cinchona alkaloid-catalyzed enantioselective fluorinations. The enantiomeric excesses of the products obtained with NFBSI **33-3** increased by 18% compared to that of (PhSO_2_)_2_NF (NFSI, **14-2**), while the chemical yields decreased (Scheme 75). Since the active fluorination agent in these reactions is considered to be the in situ generated *N*-fluorocinchona alkaloid salt, after fluorine transfer from NFBSI or NFSI to generate a counter anion, the bulky in situ generated counter anion may help enhance the % ee of the products.

#### 1-34. *p*-Substituted *N*-fluorobenzenesulfonimides

**Scheme 76:**
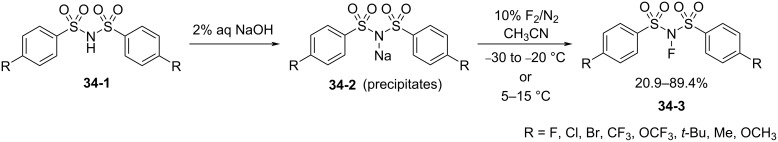
Synthesis of *p*-substituted *N*-fluorobenzenesulfonimides **34-3**.

In 2012, Yang et al. reported an efficient process for the preparation of *p*-substituted *N*-fluorobenzenesulfonimides **34-3** [103]. The traditional process involved the direct fluorination of benzensulfonimides in acetonitrile in the presence of a large excess of NaF. They prepared the sodium salts **34-2** (precipitates) with 2% aq NaOH solution and treated the air-dried precipitates with 10% F_2_/N_2_ in acetonitrile (Scheme 76). This process considerably improved the yields of the *N*-fluorobenzenesulfonimides **34-3**.

**Figure 11:**
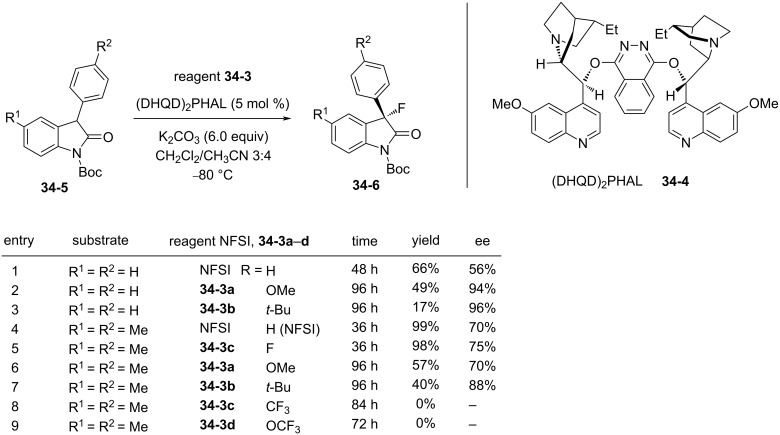
Asymmetric fluorination with **34-3** and a chiral catalyst **34-4**.

In 2013, the same group reported the enantioselective fluorination of oxindoles **34-5** with **34-3** in the presence of a catalytic amount of bis-cinchona alkaloid (DHQD)_2_PHAL **34-4** (Figure 11) [104]. In these reactions, the actual enantioselective fluorinating agents should be the *N*-F cinchona alkaloid salts formed in situ after the transfer from **34-3**. Electron-donating groups (R) afforded higher enantioselectivities compared to R = H (NFSI, **14-2**), while chemical yields were decreased. For example, while NFSI afforded 56% ee and 66% chemical yield (entry 1, Figure 11), R = OMe (**34-3a**) and *tert*-butyl (**34-3b**) gave 94% ee and 96% ee (entries 2 and 3 in Figure 11), but with only 49% and 17% chemical yields, respectively. Electron-withdrawing groups, R = CF_3_ **34-3c** and OCF_3_ **34-3d**, failed to furnish the fluorinated product under the same conditions (entries 8 and 9, Figure 11).

A year later (2014), the Yang group published the synthesis of other substituted NFSI derivatives and examined the enantioselective fluorination of 2-oxindoles catalyzed by chiral palladium complexes [105].

#### 1-35. Two Selectfluor analogues, 1-fluoro-4-[3’,4’-bis(trifluoromethyl)phenylmethyl]- and -(pentafluorophenyl)methyl-1,4-diazoniabicyclo[2.2.2]octane salts

**Scheme 77:**
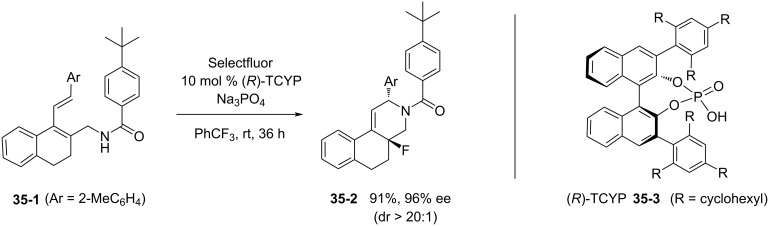
1,4-Fluoroamination with Selecfluor and a chiral catalyst.

In March 2013, Toste et al. reported the enantioselective 1,4-fluoroamination of conjugated dienes using Selectfluor in an anionic, phase-transfer catalysis in a nonpolar solvent [106]. Diene **35-1** was treated with Selectfluor and (*R*)-TCYP **35-3** as a catalyst to give **35-2** in 91% chemical yield and 96% ee (Scheme 77). In this case, the actual fluorinating species was considered to be the salt containing 1-chloromethy-4-fluoro-1,4-diazoniabicyclo[2.2.2]octane as the cation and (*R*)-TCYP as the anion, which could be formed in the non-polar organic layer.

**Figure 12:**
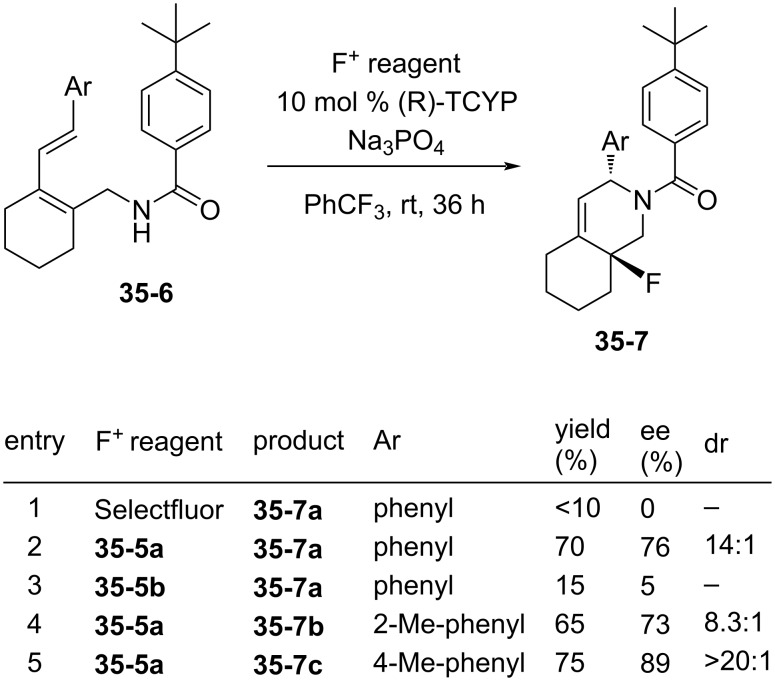
Asymmetric fluoroamination with **35-5a**, **b**.

**Scheme 78:**
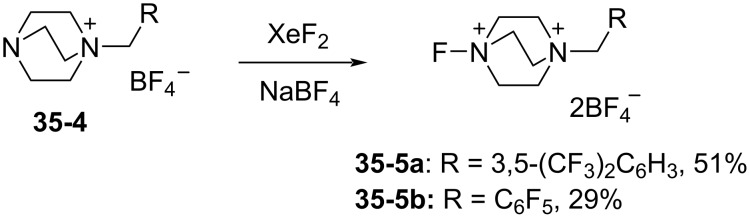
Synthesis of Selectfluor analogs **35-5a**, **b**.

However, since the reaction with the less reactive diene **35-6a** occurred in very low yield (<10%) (Figure 12, entry 1), they synthesized two new Selectfluor analogues, 1-fluoro-4-[3’,4’-bis(trifluoromethyl)phenylmethyl]- and -(pentafluorophenyl)methyl-1,4-diazoniabicyclo[2.2.2]octane bistetrafluoroborates **35-5a** and **35-5b**, by treating the precursor **35-4** with XeF_2_ in the presence of sodium tetrafluoroborate [106] (Scheme 78).

Reagent **35-5a** showed much improved chemical yields (65–75%) and enantioselectivities (73–89% ee) in fluorocyclization reactions with diene **35-6** (Figure 12, entries 2, 4, and 5) than reagent **35-5b**. Although there may be an electron-deficiency effect, this could also be attributed to the strong lipophilic effect of the bis(CF_3_)phenyl group in **35-5a** to the organic layer (PhCF_3_).

#### 1-36. Chiral dicationic DABCO-based *N*-F reagents

In June 2013, Gouverneur et al. reported the synthesis of chiral dicationic DABCO-based *N*-F reagents **36-5** that made possible the asymmetric electrophilic fluorocyclization of monoolefins with carbon nucleophiles [107]. This can be contrasted with Toste’s method, described in section 1-35 above, for asymmetric fluorocyclization of dienes with nitrogen nucleophiles using Selectfluor and an optically active phase-transfer catalyst, a reaction which did not work for monoolefins with carbon nucleophiles.

**Scheme 79:**
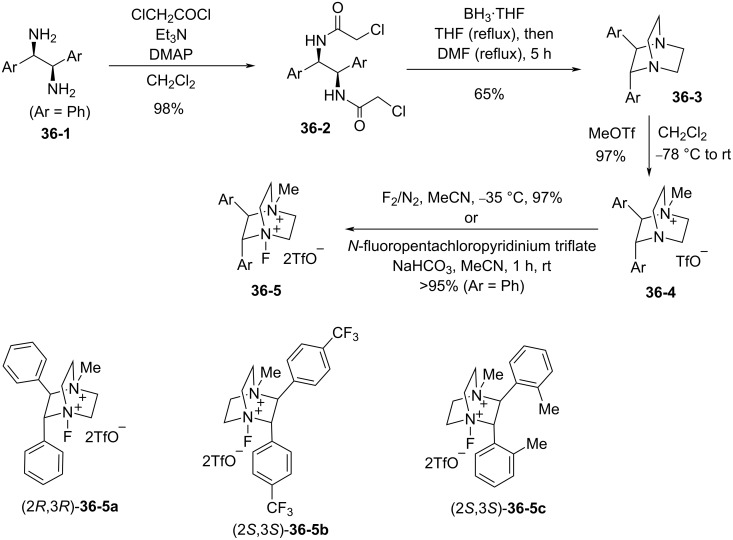
Synthesis of chiral dicationic DABCO-based *N*-F reagents **36-5**.

As shown in Scheme 79, the chiral DABCO core **36-3** was prepared from an enantiopure vicinal diamine **36-1** using the reported method. The precursor **36-4** was fluorinated with either 10% F_2_/N_2_ or *N*-fluoropentachloropyridinium triflate (**5-1v**) to produce optically active products **36-5a**, **-5b**, and **-5c** in high yields. This fluorination which uses the shelf-stable, easy-to-handle *N*-fluoropentachloropyridinium triflate (**5-1v**) was advantageous as it allowed the in situ formation of the chiral reagents **36-5**.

**Scheme 80:**
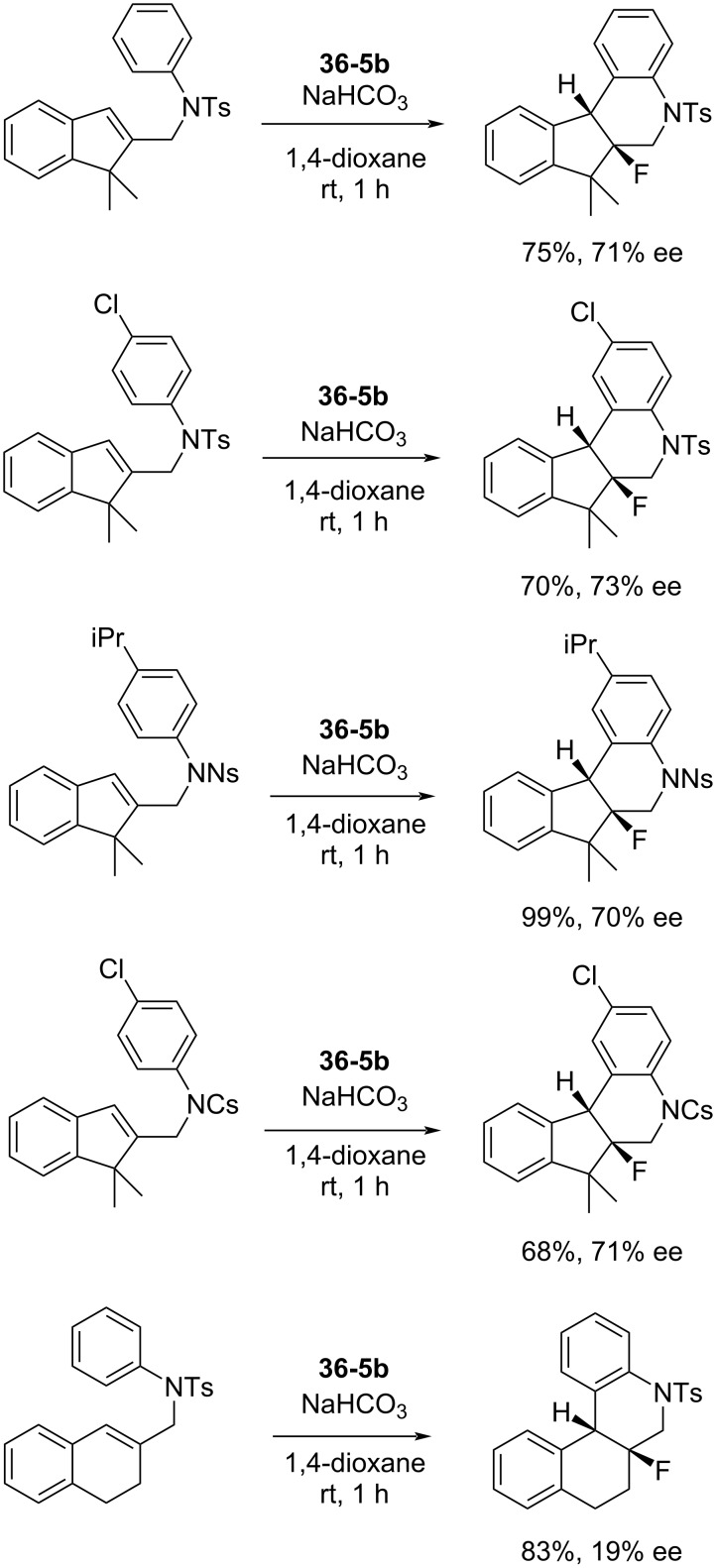
Asymmetric fluorocyclization with chiral **36-5b**.

The solubility, reactivity, and enantioselectivity of these types of reagents could be tuned by varying the substituents on the aryl rings and the *p*-trifluoromethyl-derivative **36-5b** proved to be the most efficient for this type of reaction (Scheme 80). Up to 99% chemical yield and an average of 71% ee were obtained for a series of indene derivatives. However, the enantioselectivity was low (19% ee) for the case of a dihydronaphthalene derivative (the bottom in Scheme 80). Racemates of these fluoro products were prepared in high yields with *N*-fluoro-2,6-dichloropyridinium triflate (**5-1r**).

#### 1-37. Chiral *N*-fluorobinaphthyldisulfonimides

**Scheme 81:**
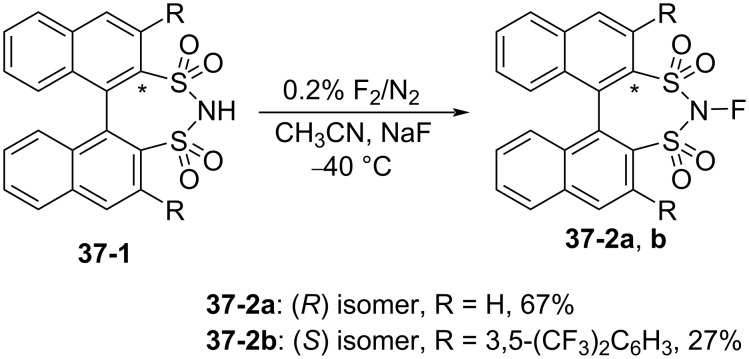
Synthesis of chiral **37-2a**,**b**.

In June 2013, the same month as the report described above from Gouverneur’s lab, Shibata, Ma, and Cahard disclosed a chiral *N*-fluorobinaphthyldisulfonimide **37-2a** and its bis[3,5-bis(trifluoromethyl)phenyl] derivative **37-2b** as enantioselective fluorination agents, compounds which were prepared in 67% and 27% yields, respectively, by fluorination of the NH precursors **37-1** with 0.2% F_2_/N_2_ (Scheme 81) [108]. In both cases, side-products were formed but not characterized. The precursors **37-1** had been synthesized by List [109] and Giernoth [110] and previously used as organocatalysts.

**Scheme 82:**
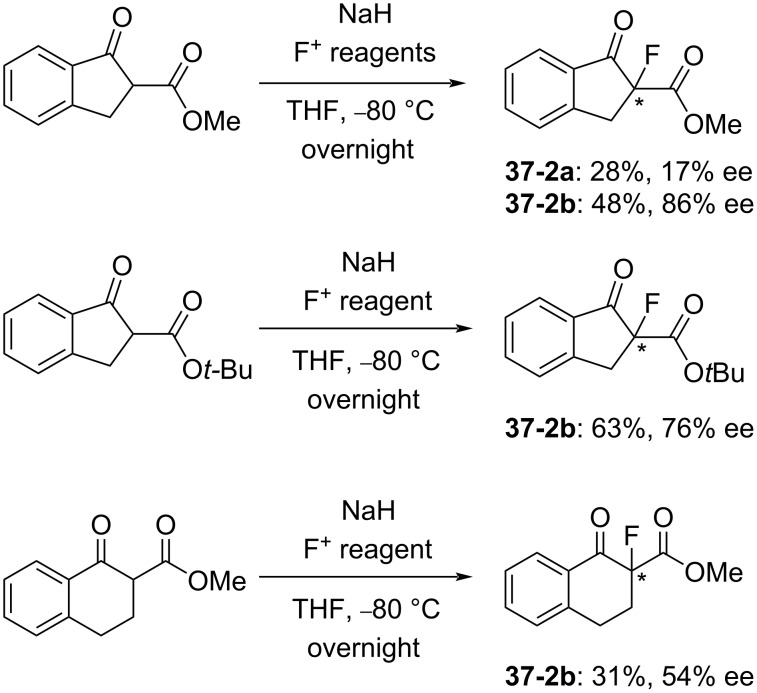
Asymmetric fluorination with chiral **37-2a**,**b**.

The fluorination ability of **37-2a** and **-2b** was evaluated with β-keto ester derivatives (Scheme 82). Moderate yields and good enantioselectivities of the fluorinated products were obtained. Reagent **37-2b** clearly gave a better selectivity than the unsubstituted **37-2a** (86% ee vs 17% ee).

**Scheme 83:**
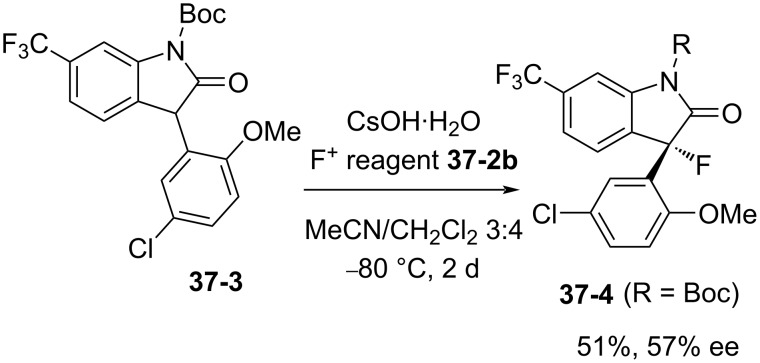
Asymmetric fluorination with chiral **37-2b**.

For an enantioselective synthesis of the potent Maxi-K potassium channel opener MaxPost **37-4** (R = H), the *N*-Boc-protected substrate **37-3** was fluorinated with **37-2b**, giving the precursor **37-4** (R = Boc) in 51% yield and with 57% ee (Scheme 83).

**Scheme 84:**
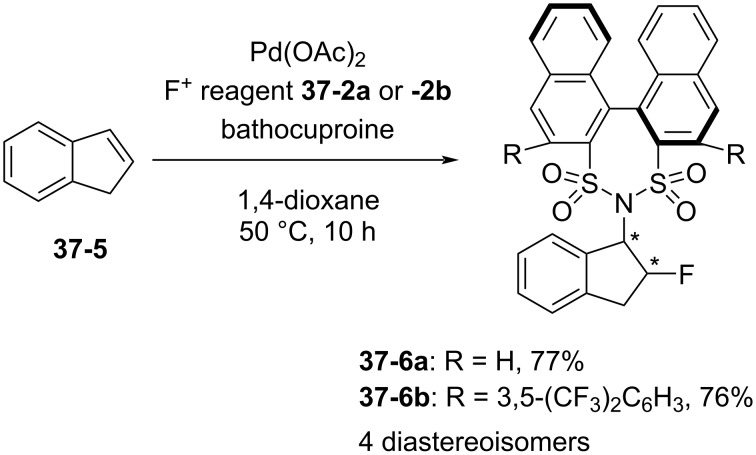
Reaction of indene with chiral **37-2a**,**b**.

The oxidative aminofluorination of indene (**37-5**) with **37-2a** and **-2b** gave four diastereomers **37-6a** and **-6b** in 77 and 76% yield, respectively; however, no asymmetric induction was observed (Scheme 84).

#### 1-38. *N*-Fluoromethanesulfonimide (Me-NFSI)

**Scheme 85:**
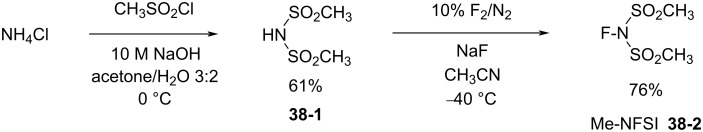
Synthesis of Me-NFSI, **38-2**.

In 2016, Shibata et al. reported the synthesis and reactivity of *N*-fluoromethanesulfonimide (Me-NFSI, **38-2**) [111]. Me-NFSI was first reported in a patent in 1994 [112], however, the reported fluorinations were vague. Prior to Shibata's report, Me-NFSI had not appeared in the literature in over 20 years. Although Me-NFSI is vulnerable with acidic protons on the methyl groups, Shibata realized the high atom economy of Me-NFSI and claimed that Me-NFSI was more effective for the fluorination of active methines than (PhSO_2_)_2_NF (NFSI, **14-2**) under Lewis acid-catalysis and non-catalysis. Me-NFSI was prepared in good yield by the fluorination of methanesulfonimide **38-1** with 10% F_2_/N_2_ in acetonitrile at −40 °C in the presence of NaF (Scheme 85).

**Scheme 86:**
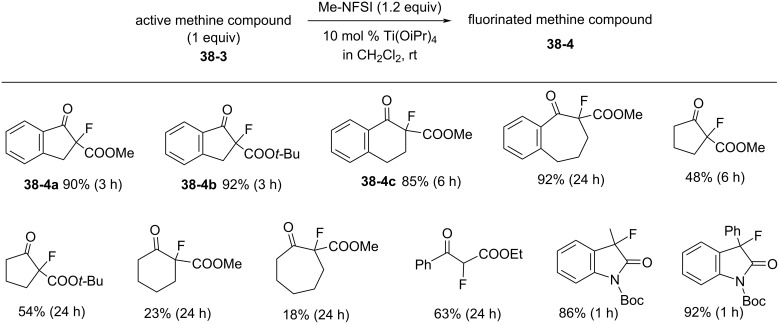
Fluorination of active methine compounds with Me-NFSI.

Many active methine compounds were fluorinated in high yields using Me-NFSI in the presence of 10 mol % Ti(OiPr)_4_ in methylene chloride as a solvent at room temperature (Scheme 86). The reactions with Me-NFSI were faster than those with NFSI. In particular, the fluorination of sterically demanding *tert*-butyl keto ester **38-3b** with Me-NFSI gave the product **38-4b** in 90% yield within only 5 min (92% in 3 h), while NFSI gave the product in 20% yield after 10 min and the yield gradually increased to 80% over 24 h. The faster reaction of Me-NFSI was explained by the activation of its *N*-F moiety via the stronger complexation with the Lewis acid Ti(OiPr)_4_ than NFSI.

**Scheme 87:**
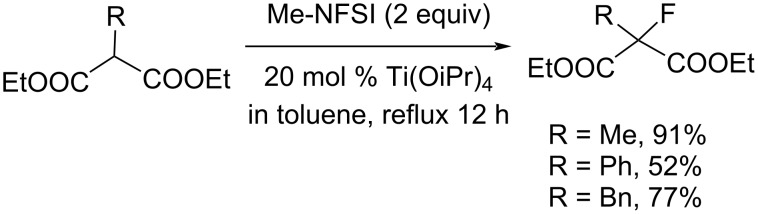
Fluorination of malonates with Me-NFSI.

For the fluorination of malonates, different conditions were needed. Treatment with 2 equiv of Me-NFSI in the presence of 20 mol % Ti(OiPr)_4_ in toluene at reflux temperature gave the fluorinated malonates in satisfactory yields (Scheme 87).

**Scheme 88:**
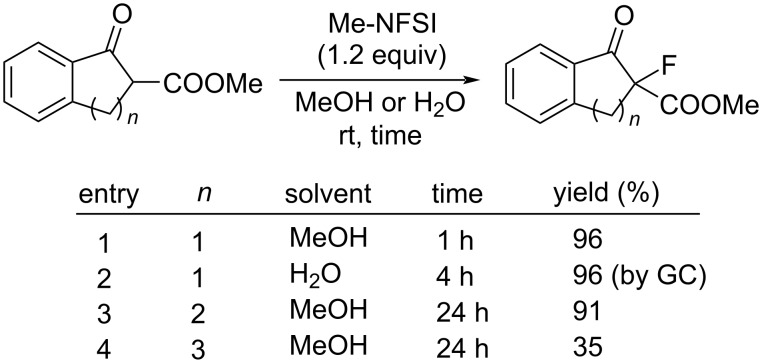
Fluorination of keto esters with Me-NFSI.

Keto esters were fluorinated in high yields with Me-NFSI employing methanol or water as solvent under catalysis-free conditions (entries 1–3, Scheme 88).

#### 1-39. An *N*–F reagent derived from the ethano-Tröger’s base

**Scheme 89:**
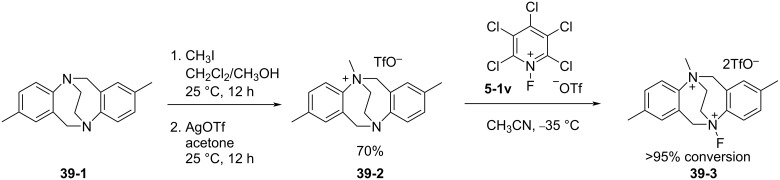
Synthesis of *N*-F **39-3** derived from the ethylene-bridged Tröger’s base.

In 2016, Gouverneur and Cvengroš reported the synthesis of a novel *N*-F reagent **39-3** derived from the ethylene-bridged Tröger’s base **39-1** [113]. The fluorination of the precursor **39-2,** obtained from **39-1,** was achieved only after reaction with *N*-fluoropentachloropyridinium triflate (**5-1v**) in acetonitrile at −35 °C, giving the *N*-F reagent **39-3** in higher than 95% conversion (Scheme 89). All other attempts to fluorinate, either with F_2_, XeF_2_, Selectfluor, or *N*-fluoro-2,6-dichloropyridinium triflate (**5-1r**) failed. This suggested that reagent **39-3** was more reactive than the other known reagents such as Selectfluor, but less active than the *N*-fluoropentachloropyridinium salts. The salt **39-3** is not stable and decomposition occurred when a solution of **39-3** in acetonitrile was left standing at room temperature for 8 hours or more. Therefore, **39-3** was best prepared with **5-1v** immediately before use. The characterization of **39-3** was made by high-resolution mass spectrometry and NMR spectroscopic analyses containing 2D ^19^F-^15^N heteronuclear correlation experiments.

**Scheme 90:**
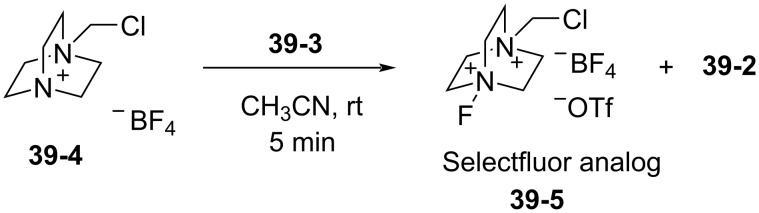
Fluorine transfer from *N*-F **39-3**.

Reagent **39-3** had the ability to formally transfer F^+^ onto the Selectfluor precursor **39-4** to form a Selectfluor analog **39-5** (Scheme 90). The fluorine transfer of **39-3** to the precursor **39-4** was completed after 5 min at room temperature in acetonitrile.

**Scheme 91:**
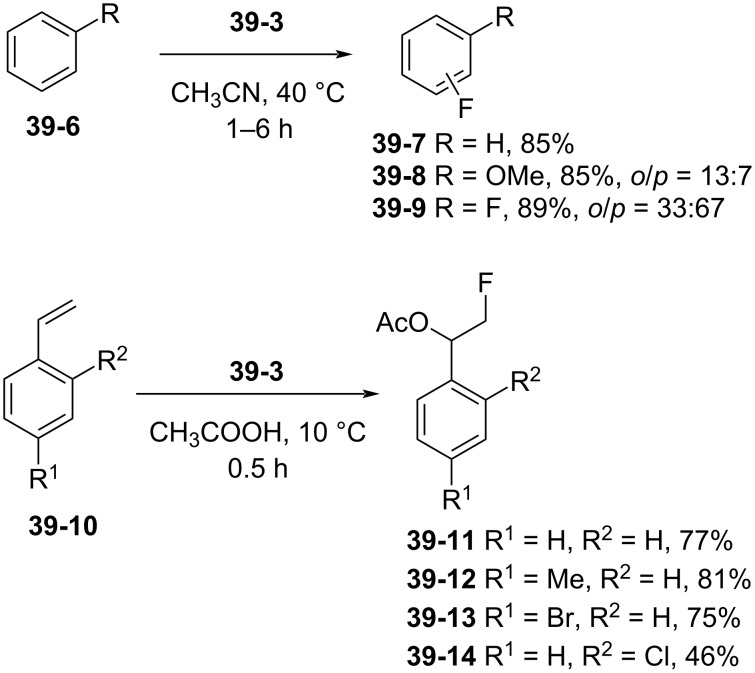
Fluorination with *N*-F **39-3**.

The ability of **39-3** to fluorinate *C*-substrates was investigated. The fluorination of benzene, anisole, and fluorobenzene with **39-3** occurred faster than with Selectfluor (Scheme 91). The fluorination of styrenes **39-10** provided the expected addition products **39-11**–**14** in good yields. However, **39-3** did not react with less-activated alkenes, such as cyclohexene.

#### 1-40. 1-Cyanomethyl-4-fluoro-1,4-diazoniabicyclo[2.2.2]octane bistriflate

**Scheme 92:**
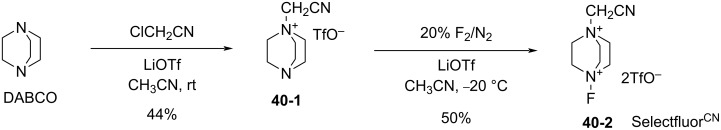
Synthesis of Selectfluor^CN^.

In early 2018, Lan and Liu reported the synthesis of 1-cyanomethyl-4-fluoro-1,4-diazoniabicyclo[2.2.2]octane bistriflate (Selectfluor^CN^) employing the same method as for Selectfluor (Scheme 92) [114].

**Scheme 93:**
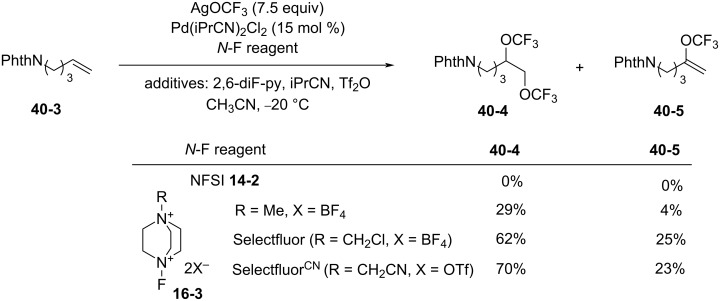
Bistrifluoromethoxylation of alkenes using Selectfluor^CN^.

As shown in Scheme 93, Selectfluor^CN^ was used as an oxidant to improve the Pd-catalyzed bistrifluoromethoxylation of alkenes with AgOCF_3_. It was shown that the yield of the bis(CF_3_O) product **40-4** increased as the fluorinating power of the *N*-F reagent increased in the order of NFSI < **16-3** (R = Me) < Selectfluor (R = CH_2_Cl) < Selectfluor^CN^ (R = CH_2_CN). A considerable number of olefin derivatives were bistrifluoromethoxylated using this method with Selectfluor^CN^ [114].

#### 1-41. *N*-Fluoro-*N*-aryl-arenesulfonamides (NFAS)

**Figure 13:**
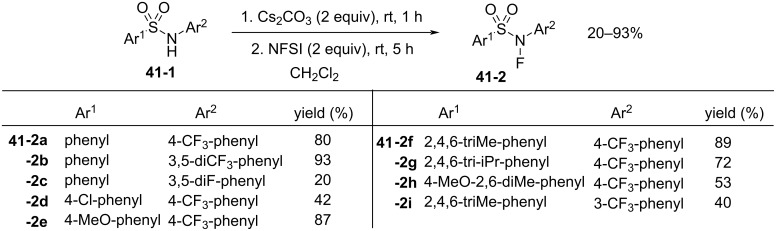
Synthesis of NFAS **41-2**.

In 2018, Zipse and Renaud reported the synthesis of a series of *N*-fluoro-*N*-aryl-arenesulfonamides (NFAS) **41-2** suitable for radical fluorination, by reacting **41-1** with NFSI in the presence of cesium carbonate in methylene chloride and in moderate to good yields (Figure 13) [115]. They suggested that NFAS belongs to a third generation of radical fluorinating agents.

**Scheme 94:**
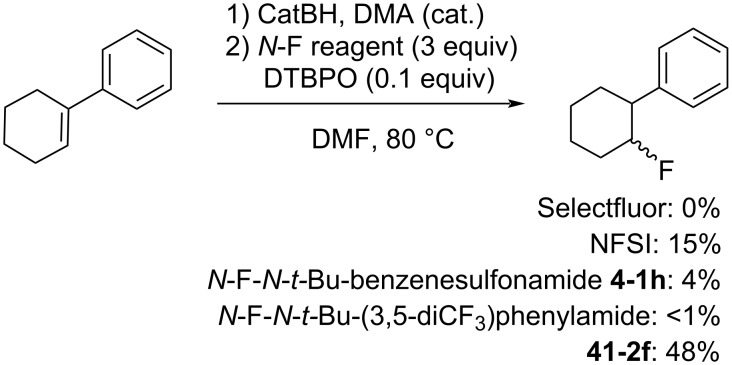
Radical fluorination with different *N*-F reagents.

These authors designed NFAS **41-2a**–**i** as having a low *N*-F homolytic bond-dissociation energy (BDE). They demonstrated experimentally that NFAS **41-2** were more useful for radical fluorinations than *N*-fluoro-*N*-alkyl-arenesulfonamides **4-1**, *N*-fluoro-*N*-alkyl-arylamides, NFSI **14-2**, and Selectfluor, all of which have higher N–F homolytic BDEs than NFAS **41-2** (Scheme 94).

**Scheme 95:**
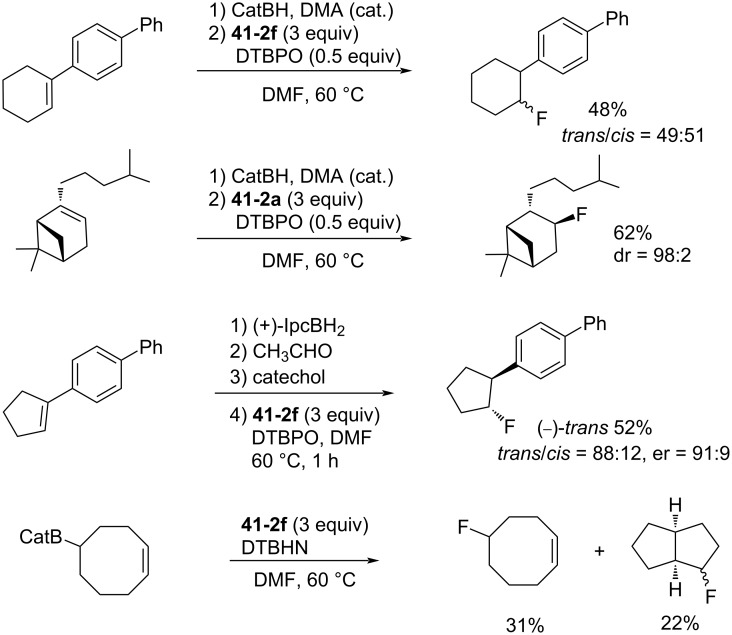
Radical fluorination of alkenes with NFAS **41-2**.

The hydrofluorinated products were obtained in moderate yields along with good stereoselectivities in some cases (Scheme 95).

**Scheme 96:**
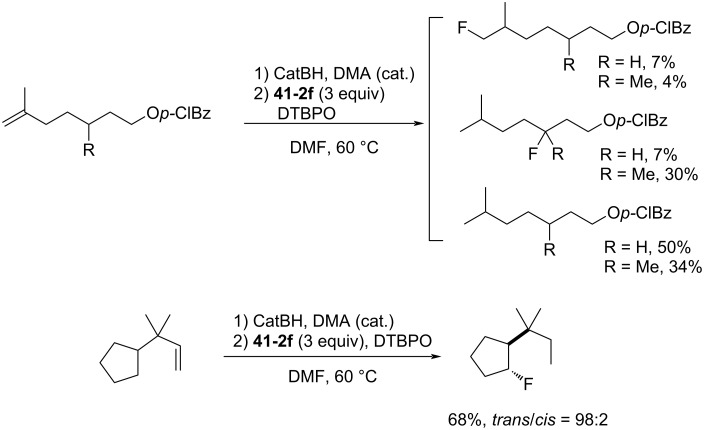
Radical fluorination of alkenes with NFAS **41-2f**.

As for the less nucleophilic terminal alkenes, remote fluorinated products were favored via the 1,5 H-atom abstraction process (Scheme 96).

**Scheme 97:**
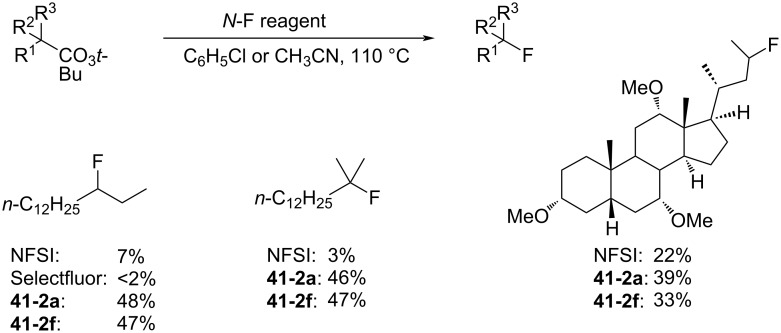
Decarboxylative fluorination with NFAS **41-2a**,**f**.

Decarboxylative fluorination of *tert*-butyl peresters also proved that these NFAS (**41-2a** and **-2f**) were better choices than NFSI and Selectfluor (Scheme 97).

### 2. Evaluation of fluorinating power of *N*-F fluorinating agents

It emerges from the overview above that many *N*-F fluorination reagent variants have been developed, each with a different fluorination power (reactivity). Therefore, the power ordering of *N*-F reagents has become a matter of interest. Early on, Umemoto et al. revealed that the fluorinating power of *N*-fluoropyridinium salt systems could be tuned by the electron density at the nitrogen site, a property which is controlled by the electronic nature of the substituents. In order to quantitatively explain the variability of the *N*-F reagents, many attempts have been made: Theoretical calculations were performed by Hachisuka and Umemoto in 1991 [116–117], Woolf in 1994 [118], and Fainzil’berg in 1994 and 2001 [119–120]; N-F 19-fluorine NMR chemical shift analysis was accessed by Umemoto in 1991 [34]; measurements of peak reduction potentials were carried out by Gilicinski in 1992 [121], Differding in 1992 [122], Evans in 1999 [123], Zhang in 2013 [104], and reported by Umemoto in 2016 [124]; relative fluorination rates (kinetic data) were conducted by Togni in 2004 [125], and electromotive forces with Li, Mg, and Zn metals were reported by Umemoto in 2016 [124].

**Scheme 98:**
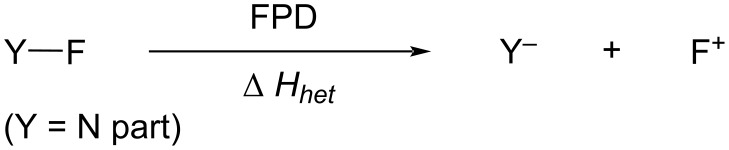
Fluorine plus detachment (FPD).

In a significant advancement in this regard, in 2016 Xue and Cheng published a comprehensive energetic scale for the quantitative estimation of the fluorinating power of *N*-F reagents [126–127]. They applied the Fluorine Plus Detachment (FPD) energy parameter, introduced by Christe and Dixon in 1992 [128], to electrophilic *N*-F reagents and calculated the FPD energies of 130 *N*-F reagents (Scheme 98).

**Figure 14:**
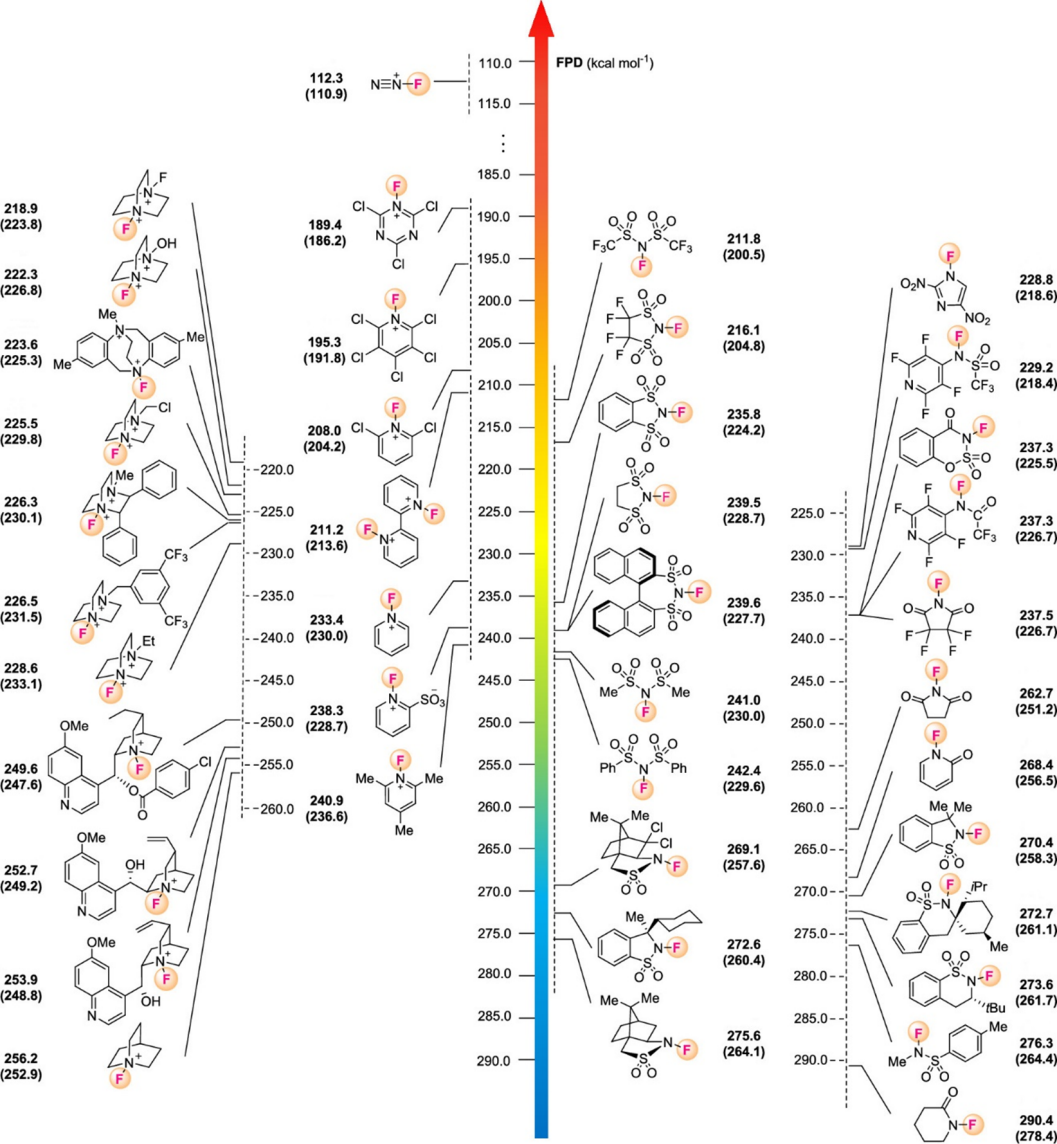
FPD values of representative *N*-F reagents in CH_2_Cl_2_ and CH_3_CN (in parentheses). Adapted with permission from ref. [127]. Copyright 2020 American Chemical Society.

As seen in Figure 14, a large number of *N*-F reagents were clearly arranged in their power order; the smaller the FPD value the stronger the fluorinating power. This energetic scale is useful for an evaluation of the electrophilic fluorinating power of the *N*-F reagents.

**Scheme 99:**
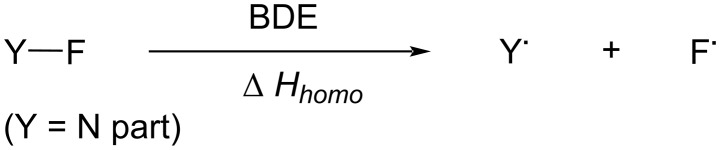
*N*-F homolytic bond dissociation energy (BDE).

**Figure 15:**
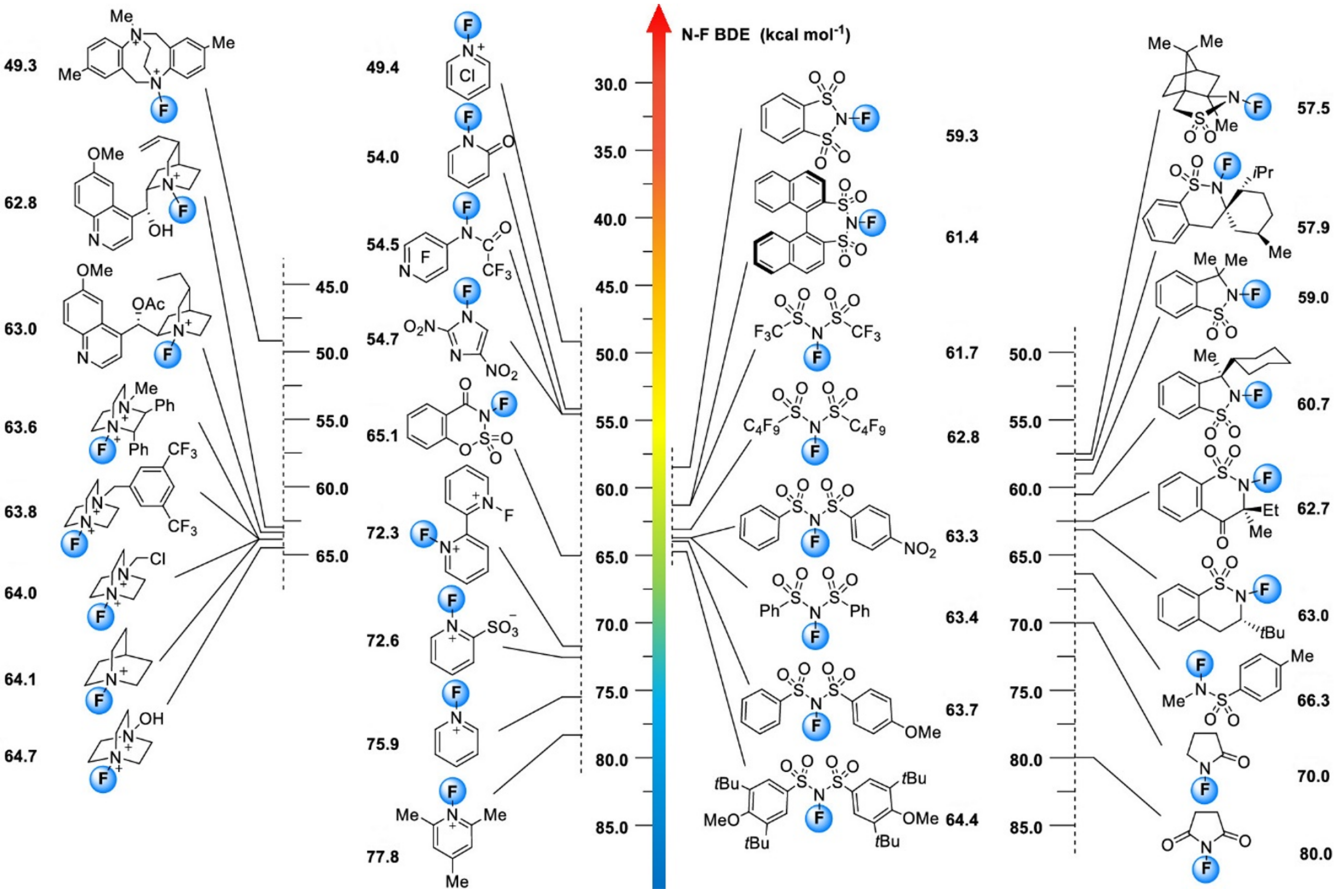
BDE values of representative *N*-F reagents in CH_3_CN. Adapted with permission from ref. [127]. Copyright 2020 American Chemical Society.

In addition, since some *N*-F reagents have been proven to be useful radical fluorinating agents [129–135], Xue and Cheng introduced the *N*-F homolytic bond-dissociation energy (BDE) parameter for radical fluorination abilities of *N*-F reagents in 2017 (Scheme 99) [127,136]. They calculated the BDE strength of 88 *N*-F reagents and clearly arranged them in terms of their radical fluorination power; the smaller the BDE value the stronger the radical fluorinating power (Figure 15). The BDE is useful for identifying and designing new atomic fluorine sources.

**Figure 16:**
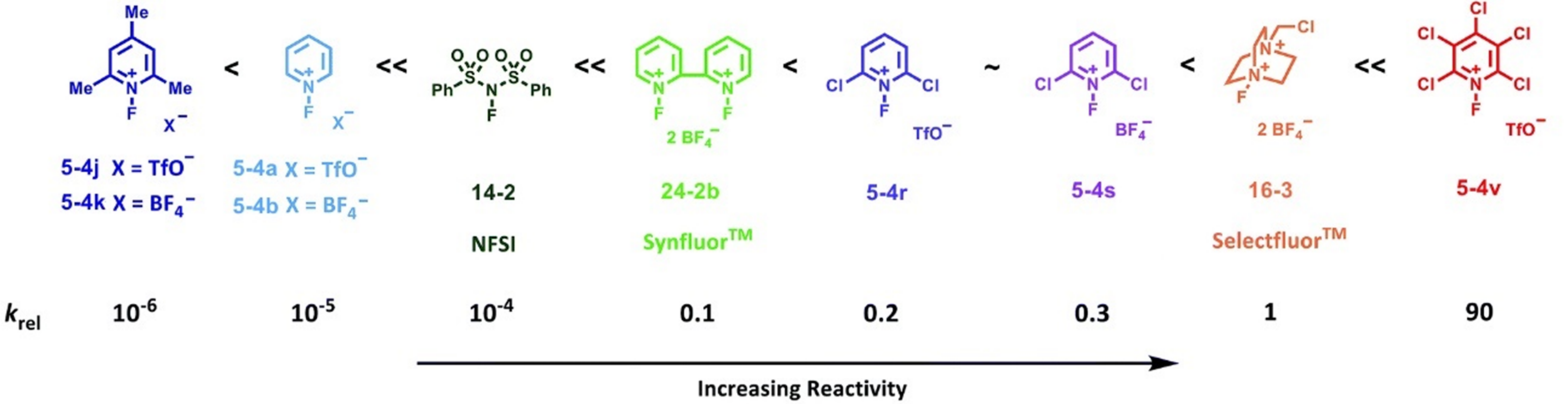
Quantitative reactivity scale for popular *N*-F reagents. Adapted with permission from ref. [138], published by the Royal Society of Chemistry.

In 2018, another significant advancement was reported. Since FPD and BDE are thermodynamic parameters, they cannot be directly used for predicting reaction rates. Mayr et al. [137] and Hodgson et al. [138] independently reported the quantitative reactivity scales for electrophilic fluorinations of popular *N*-F reagents. They provided detailed kinetic fluorination studies of a series of popular *N*-F fluorinating agents such as *N*-fluoropyridinium salt derivatives, NFSI, and Selectfluor^TM^ with enamines, carbanions, or 1,3-diketones. Figure 16 shows the relative reactivity scale of the popular *N*-F reagents, which covers eight orders of magnitude. These reactivity scales can be used for predicting the actual fluorination rates of *N*-F fluorinating agents. Hodgson et al. further advanced their kinetic studies with *N*-F fluorinating agents in 2019 [139] and 2020 [140].

In 2019, Zhang et al. reported detailed theoretical calculations of the fluorination of benzene with 16 different disubstituted *N*-fluoropyridinum salts in acetonitrile solution and as a result, suggested a 2,6-dinitro-substituted *N*-fluoropyridinium salt as the most promising fluorinating reagent [141].

### 3. Reaction mechanisms of *N*-F fluorinating agents

**Scheme 100:**
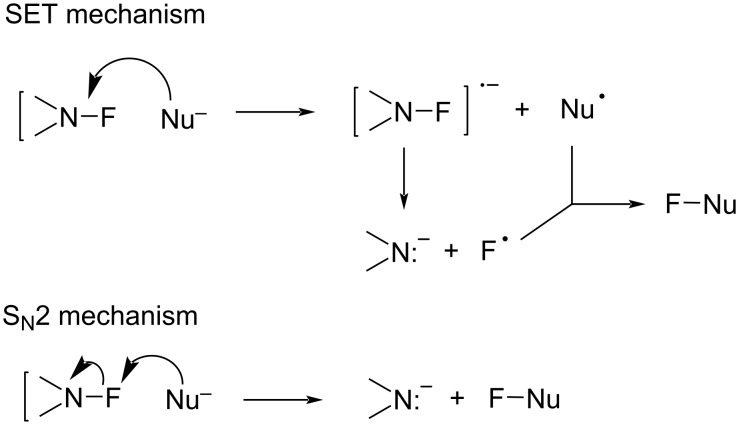
SET and S_N_2 mechanisms.

Another noteworthy issue is the reaction mechanism for the fluorination with *N*-F reagents. There have been long standing arguments regarding which mechanism is operating in these fluorinations: a single-electron transfer (SET) or a nucleophilic substitution (S_N_2) mechanism (Scheme 100) [22,142]. In the SET process, a single-electron transfer from a substrate (Nu^−^) to a *N*-F reagent [N-F] occurs to form a radical anion [N-F]**^·^**^−^ and a Nu^·^ radical, and the former undertakes the homolytic N–F bond cleavage to give a F**^·^** radical and a N:^−^ anion, and then the F^·^ radical and the Nu^·^ radical combine to generate the final product Nu–F. In the S_N_2 mechanism, a nucleophile directly attacks the F atom of the *N*-F reagent to give the fluorinated product via the heterolytic cleavage of the N–F bond. The key point is whether the N–F bond cleaves homolytically or heterolytically. The prime cause of this argument is based on the fact that the formation of F^+^ requires extremely high energy (ionization potential of F, 1681.0 kJ/mol) and contrasts significantly with that of other halogenations (Cl, 1251.2 kJ/mol; Br, 1139.9 kJ/mol).

**Scheme 101:**
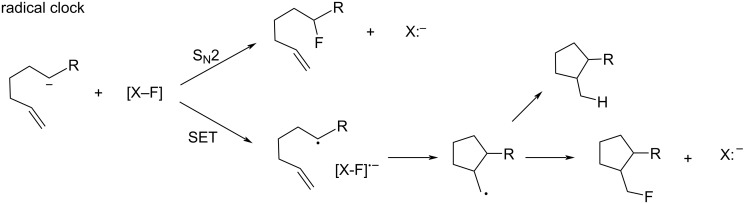
Radical clock reactions.

In 1991, Umemoto et al. explained the observed fluorination reactions of *N*-fluoropyridinium salts with anionic and neutral substrates by SET processes involving a F**^·^** radical species [32]. Soon after, in 1992, Kochi et al. reported fluorination reactions of aromatic compounds with *N*-fluoropyridinium salts via electron donor–acceptor complexes, with outcomes which also supported the SET mechanism [143–144]. In 1992, DesMarteau et al. proposed a similar SET process for the fluorination with *N*-fluoroperfluoroalkanesulfonimide [48]. On the other hand, in 1991, Differding and co-worker investigated radical clock experiments to monitor the reaction for the presence of intermediate radicals [145] (Scheme 101).

**Scheme 102:**
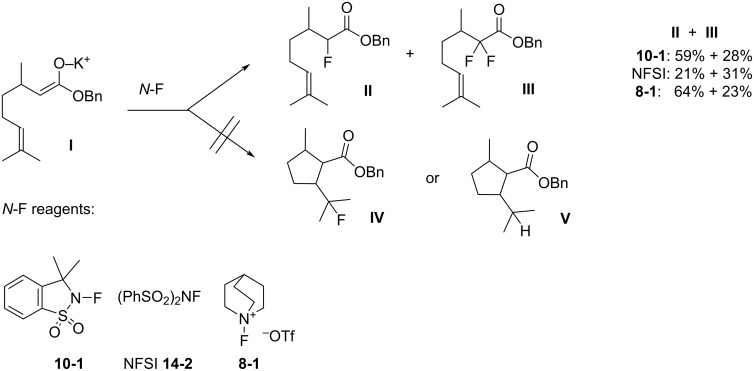
Reaction of potassium enolate of citronellic ester with *N*-F reagents, **10-1**, NFSI, and **8-1**.

As illustrated in Scheme 102, the reactions of potassium enolate **I** of citronellic ester with the *N*-F reagents **10-1**, **14-2** (NFSI), or **8-1** produced only the non-cyclized products **II** and **III**. They also showed that the observed reaction rates were much faster than the calculated electron-transfer rates and therefore they concluded that a S_N_2 mechanism occurred to give the fluorinated products [146].

**Scheme 103:**
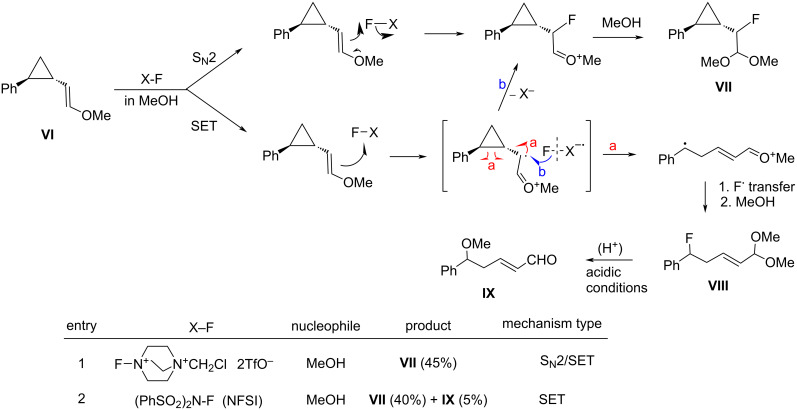
Reaction of compound **IV** with Selectfluor (OTf) and NFSI.

In 1995, based on the study on the fluorination reactions of the *N*-F reagent NFOBS **13-2**, Davis et al. described that all of their available data supported the S_N_2 reaction process, but the SET mechanism could not be rigorously excluded [58]. In 1998, Zupan et al. studied the reactions of norbornene with Selectfluor, NFTh **19-2**, and *N*-fluoro-2,6-dichloropyridinium triflate [147]. Although their results ended toward S_N_2, they too could not completely exclude the SET. Soon after, in 1999, Wong et al. reported their studies using compound **VI** having a phenyl-cyclopropyl moiety which was the hypersensitive radical probe (rate: 10^11^ s^−1^) (Scheme 103) [148].

They found that Selectfluor (OTf salt) produced product **VII** in 45% yield with no rearrangement products despite the complete consumption of **VI** (entry 1, Scheme 103). This result is aligned with the S_N_2 mechanism, however, the reaction of **VI** with Selecfluor was completely blocked by the radical scavenger, 2,2,6,6-tetramethylpiperidinoxyl (TEMPO), which suggested a SET mechanism. The reaction of **VI** with NFSI gave **VII** (40%) and a product **IX** (5%) derived from the rearrangement fluoro product **VIII** (entry 2 in Scheme 103). This result is consistent with the SET mechanism. In their review published in 2005 [149], Wong discussed these results in more detail. Since the reactivity of the radical (F^·^) is extremely fast and diffusion-controlled, the combination of F^·^ with any alkyl radical R^·^ should proceed at an equal or greater rate than the cyclization of the alkyl radical as observed in the case of Differding [145] and the ring-opening of the cyclopropyl radical in the case of Wong [148]. Despite extensive research, the mechanism remains unclear as a result of the difficulty of assessing reaction rates. The key may be the lifetime of the intermediate N-F radical or radical anion, [N-F]^·^ or [N-F]^·−^, which releases the radical F^·^. Finally, Wong mentioned that, if these radicals react faster than the rates of diffusion, anything beyond that would probably remain unproven [149].

In the meantime, in 2002, Togni and Rothlisberger provided evidence which strongly supported the SET mechanism from their studies on the fluorination mechanism of β-ketoesters with Selectfluor and a chiral TiCl_2_(TADDOLato) catalyst [150]. Their computational study suggested a one-electron transfer followed by simultaneous N–F bond cleavage and the accompanying experimental outcomes demonstrated that a chlorination side reaction was quenched by a radical scavenger, while the fluorination reaction was unaffected. Since Selectfluor is inert towards chloride, they proposed that any [N-F]**^·^** radical formed by the SET process would react with chloride ions (Cl^−^) present in the reaction solution to form the relatively stable Cl**^·^** radical, which caused the side chlorination.

On the other hand, in 2002, Laali and co-worker reported that competitive fluorination studies of mesitylene and durene with Selecfluor in CH_3_CN and an ionic liquid supported the conventional polar mechanism (S_N_2) [151]. However, in 2006, Stavber et al. made similar competition studies and proposed a SET process as the dominant mechanism that explained the higher reactivity of durene viz-à-viz mesitylene towards Selectfluor in aq CH_3_CN [152]. In 2009, Shubin et al. performed competitive studies of mesitylene and durene with NFSI under solvent-free reaction conditions and reported that the reaction rate ratio (*k*_Mes_/*k*_Dur_) followed a polar mechanism [153].

Since 2015, many papers supporting either SET or S_N_2 mechanisms have appeared, including those reported by C. Liu et al. (SET) in 2015 [154], Chen et al. (S_N_2) in 2016 [155], Tang et al. (SET) in 2016 [156], F. Liu et al. (SET) in 2016 [157] and 2017 [158], Mayr et al. (S_N_2) in 2018 [137], Hodgson et al. (S_N_2) in 2018 [138], Li et al. (SET) in 2018 [159], Shi et al. (SET) in 2018 [160], Li et al. (SET) in 2019 [161], Mernyak et al. (SET) in 2019 [162], and Nelson et al. (S_N_2) in 2019 [163].

**Scheme 104:**
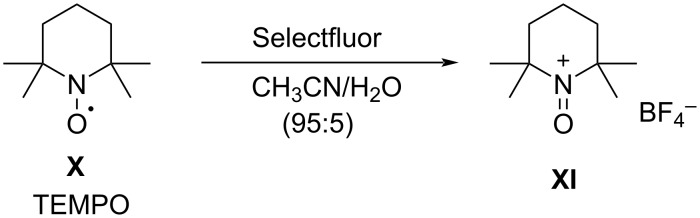
Reaction of TEMPO with Selecfluor.

TEMPO has been often used as a radical scavenger to explore the reaction mechanisms of *N*-F fluorinating agents but, in Nelson's paper [163] in 2019, it was revealed that Selectfluor reacted with TEMPO (**X**) to form the oxoammonium salt **XI** (Scheme 104). This author concluded that the use of TEMPO as a radical trap in the reactions with Selectfluor was unreliable. This conclusion was subsequently confirmed by Borodkin et al. [164]. The latest review [165] by Hodgson and co-worker in 2021 discussed the reactivities and reaction mechanisms of electrophilic *N*-F fluorinating agents in detail.

## Conclusion

A historical timeline on the progress of *N*-F fluorinating reagents and their fluorinations has been described in this review. Table 1 shows a historical list of all the *N*-F reagents. The fluorination power and reaction mechanism of the *N*-F fluorinating agents have also been summarized briefly and from a historical perspective. The fluorination reactions described here for each *N*-F fluorinating agent are those reported at the time. The synthetic applications of each *N*-F reagent since then are too numerous to be covered in this review. As many reviews on the reactions and applications of the *N*-F fluorinating agents have been published [39,133,142,165–181], readers are recommended to consult these reviews.

**Table 1:** Chronological list of the development of *N*-F fluorinating agents.

year	structure	“F” source	yield	references

1964	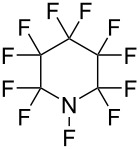	HF	7.5–13%	[16–22]
1981	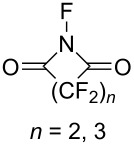	XeF_2_	50–65%	[23]
1983	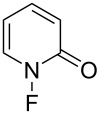	5% F_2_/N_2_	63%	[24–25]
1984	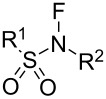 R^1^ = *p*-tolyl, *n*-butyl; R^2^ = methyl, *tert*-butyl, exo/endo-2-norbornyl, cyclohexyl, neopentyl	1–5% F_2_/N_2_	11–71%	[26–27]
1986– 1991	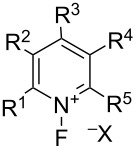 X = OTf, BF_4_, PF_6_, AsF_6_, SbF_6_, ONf, OMs, etc. R^1^, R^2,^ R^3^, R^4^, R^5^ = H, Me, Cl, F, COOMe, OMe, CH_2_OMe, CH_2_OAc, CF_3_, CN, NO_2_, OMenth, etc.; (total 62 examples)	10% F_2_/N_2_	15–96%	[28–34,36–41]
1986	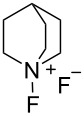	100% F_2_	86%	[43–44]
1987	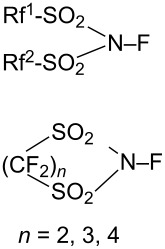 Rf^1,2^ = CF_3_, C_4_F_9_, C_6_F_13_	100% F_2_	61–96%	[45–49]
1988	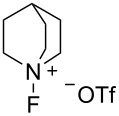	100% F_2_	80–88%	[50–51]
1988	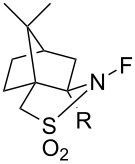 R = H, CH_3_	10% F_2_/N_2_	75–80%	[52]
1989	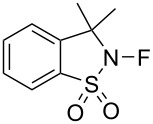	10% F_2_/N_2_	74%	[53]
1990	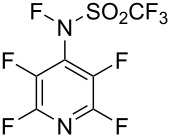	100% F_2_	89%	[54]
1990	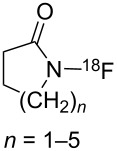	0.05% ^18^F_2_/Ne	33–79%	[55–56]
1991	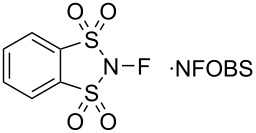	10% F_2_/N_2_	>90%	[57–58]
1991	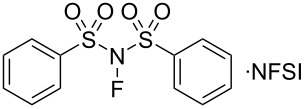	10% F_2_/N_2_	70%	[59]
1991	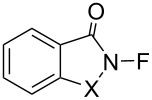 X = SO_2_, CO	Cs^+-^OSO_2_OF	48–69%	[60]
1992	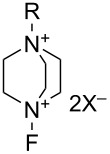 X = OTf, BF_4_ R = CH_3_, CH_2_Cl, CH_2_CF_3_ Selectfluor^TM^ (R = CH_2_Cl, X = BF_4_)	10% F_2_/N_2_	87–95%	[42,61–74]
1993	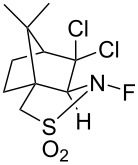	10% F_2_/N_2_	68%	[75]
1995	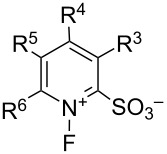 R^3^, R^4^, R^5^, R^6^ = H, CH_3_, CF_3_, Cl, C_2_H_5_, *tert*-butyl	10% F_2_/N_2_	65–95%	[76]
1995	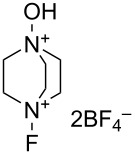 NFTh/Accufluor^TM^	10% F_2_/N_2_	75%	[78–80]
1995	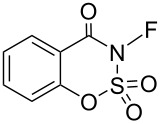	5% F_2_/N_2_	83%	[81]
1996	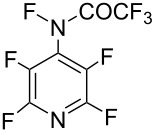	100% F_2_	75%	[82]
1996	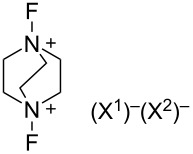 X^1^, X^2^ = OTf, BF_4_, HSO_4_, SbF_6_, PF_6_, etc.	10% F_2_/N_2_	56–87%	[83]
1997	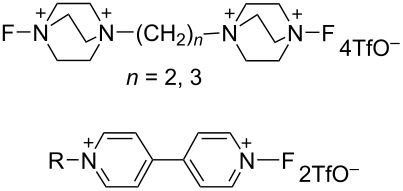 R = BF_3_, F, CH_3_	10% F_2_/N_2_	62–82%	[85]
1997	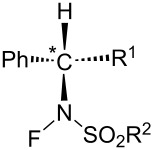 R^1^ = CH_3_, CH_2_OCOCH_3_ R^2^ = CH_3_, *p*-tolyl	FClO_3_	13–52%	[92]
1998	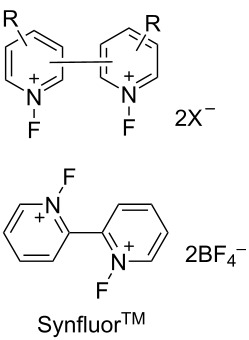 X = OTf, BF_4_, PF_6_, SbF_6_ bipyridyl = 2,2', 2,4', 3,3', 4,4'; R = H, CH_3_, Cl, CF_3_, Ph, COOCH_3_	10-20% F_2_/N_2_	64–97%	[86]
1998	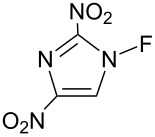	5% F_2_/N_2_	–	[87–88]
1999	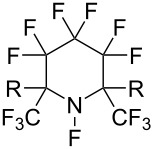 R = CH_3_, CF_3_	10% F_2_/N_2_	65–82%	[89]
1999	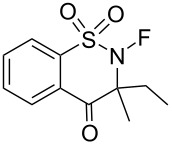	FClO_3_	71%	[90]
1999	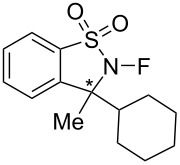 (*R*)-(+) (*S*)-(–)	15% F_2_/He	51–65%	[91]
2000	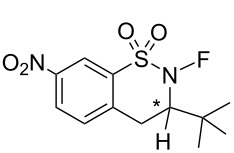 (*R*), (*S*)	FClO_3_	66–83%	[93]
2000	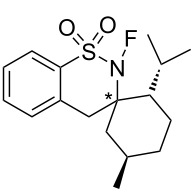 (*S*,*R*,*R*), (*S*,*S*,*R*)	FClO_3_	44–81%	[94]
2000	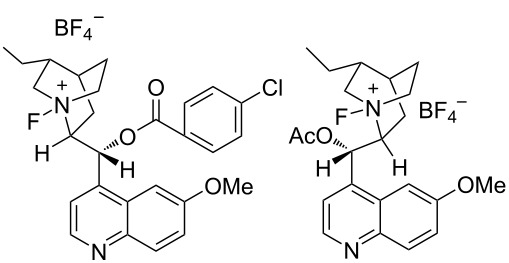	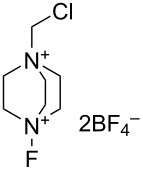 Selectfluor^TM^	prepared in situ	[95–96]
2000	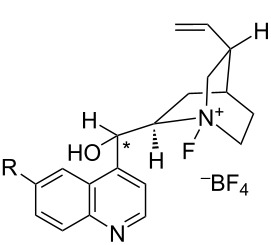 R = H, OCH_3_	Selectfluor^TM^	84%	[97–98]
2003	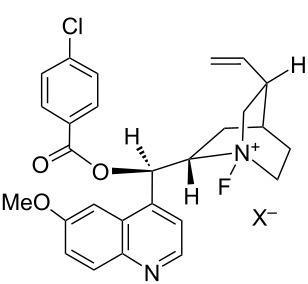 X = BF_4_, OTf, N(SO_2_Ph)_2_	Selectfluor (F-TEDA-BF_4_) F-TEDA-OTf NFTh NFSI *N*-F-diCl-pyridinium BF_4_	100%	[99]
2003	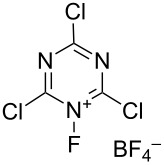	100% F_2_	95%	[100]
2011	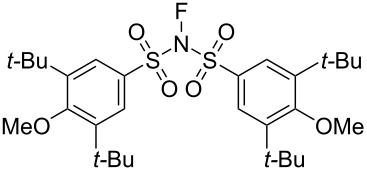 NFBSI	10% F_2_/N_2_	57%	[102]
2012	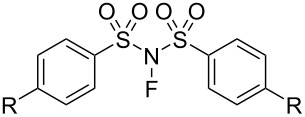 R = F, Cl, Br, CF_3_, OCF_3_, *t*-Bu, Me, OCH_3_	10% F_2_/N_2_	20.9–89.4%	[103–105]
2013	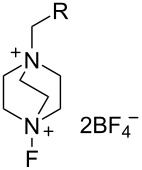 R = 3,5-(CF_3_)_2_C_6_H_3_, C_6_F_5_	XeF_2_	29–51%	[106]
2013	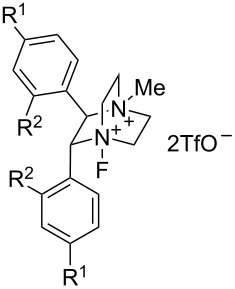 R^1^ = H, CF_3_; R^2^ = H, CH_3_	10% F_2_/N_2_ or 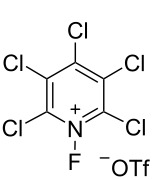	>95%	[107]
2013	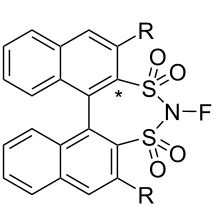 (*R*), R = H; (*S*), R = 3,5-(CF_3_)_2_C_6_H_3_	0.2% F_2_/N_2_	27–67%	[108]
2016	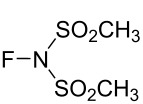 Me-NFSI	10% F_2_/N_2_	76%	[111]
2016	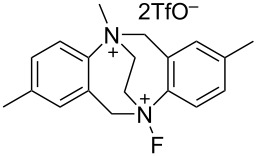	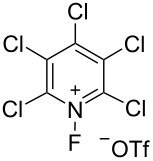	>95% conversion	[113]
2018	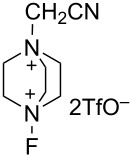	20% F_2_/N_2_	50%	[114]
2018	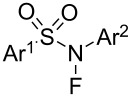 Ar^1^ = C_6_H_5_, 4-Cl-C_6_H_4_, 2,4,6-triMe-C_6_H_2_, etc.; Ar^2^ = 4-CF_3_-C_6_H_4_, 3-CF_3_-C_6_H_4_, 3,5-diCF_3_C_6_H_3_	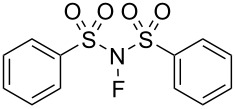 NFSI	20–93%	[115]

The seminal work of perfluoro-*N*-fluoropiperidine by Banks was reported in 1964 [16]. However, this compound could not be contemplated for two decades because of the very low yields in its production and fluorination reactions. At that time, FClO_3_ [10] and *O*-F reagents such as CF_3_OF and CF_2_(OF)_2_ [11] were used as electrophilic fluorinating agents despite their inherent safety issues. The real attention on *N*-F compounds started in the 1980’s when the first report on a *N*-F fluorinating agent, *N*-fluoro-2-pyridone, was published by Purrington in 1983 [24]. The next year, Barnette reported *N*-fluoro-*N*-alkylarenesulfonamides that were easy-to-handle, stable crystalline compounds useful for the fluorination of carbanions [26]. Soon after (1986), Umemoto reported a series of *N*-fluoropyridinium triflates as easy-to-handle, reactive, and widely applicable reagents [28–29].

Since then, many *N*-F fluorinating agents have been reported, one after another, as follows: powerful *N*-fluorotrifluoromethanesulfonimide by DesMarteau in 1987 [45], mild *N*-fluorosultams including first chiral *N*-fluorosultams by Lang in 1988 [52] and 1989 [53], less reactive *N*-fluorolactams by Sathyamurthy in 1990 [55], moderate *N*-fluoro-*o*-benzenedisulfonimide (NFOBS) [57] and *N*-fluorobenzenesulfonimide (NFSI) [59] by Davis and Differding, respectively, in 1991, and powerful 1-chloromethyl-4-fluoro-1,4-diazoniabicyclo[2.2.2]octane bistetrafluoroborate (Selectfluor^TM^) by Banks in 1992 [42]. Currently popular *N*-F fluorinating agents such as *N*-fluoropyridinium salts, NFSI, and Selectfluor were developed within a decade from the first Purrington’s report (1983).

After this first rush, other notable *N*-F reagents were developed such as a series of zwitterionic *N*-fluoropyridinium salts by Umemoto in 1995 [76], another Selectfluor-type reagent, 1-fluoro-4-hydroxy-1,4-diazoniabicyclo[2.2.2]octane bistetrafluoroborate (NFTh/Accufluor^TM^) by Poss and Shia in 1995 [78], a more powerful reagent, 1,4-difluoro-1,4-diazoniabicyclo[2.2.2]octane bistetrafluoroborate by Umemoto in 1996 [83], and a powerful and high fluorine-content reagent, *N*,*N’*-difluoro-2,2’-bipyridinium bistetrafluoroborate (Synfluor^TM^) by Umemoto in 1998 [86].

In 2000, practical chiral reagents, *N*-fluorinated chiral cinchona alkaloids were reported by Shibata and Takeuchi [95] and by Cahard [97]. In 2003, Cahard demonstrated that the chiral *N*-fluorocinchona alkaloids could be prepared quantitatively with not only Selectfluor but also with other powerful *N*-F fluorinating agents such as NFTh, NFSI, and *N*-fluoro-2,6-dichloropyridinium tetrafluoroborate [99]. After a break of a decade, in 2013, chiral dicationic DABCO-based *N*-F reagents **36-5** and chiral *N*-fluorobinaphthyldisulfonimides **37-2** were reported by Gouverneur [107] and by Shibata, Ma, and Cahard [108], respectively. In 2016, Shibata reported *N*-fluoromethanesulfonimide (Me-NFSI) as a high atom-economy reagent [111] and Gouverneur and Cvingroš reported a novel *N*-F reagent **39-3** derived from the ethylene-bridged Tröger base [113]. More recently, Zipse and Renaud in 2018, disclosed *N*-fluoro-*N*-aryl-arenesulfonamides (NFAS) as radical fluorinating agents [115].

A very large number of *N*-F fluorinating agents have been developed so far and a considerable number of easy-to-handle *N*-F reagents have been commercialized. In particular, Selectfluor, NFSI, and *N*-fluoropyridinium salt derivatives are produced in factories on a large scale. This has greatly contributed to the remarkable advancement of organofluorine chemistry. Before the appearance of *N*-F reagents, electrophilic fluorinating agents were thought dangerous and difficult to handle and special techniques were required. Chemists avoided them. Now, practicing chemists use them without any problem. The *N*-F reagents have brought such a dramatic change that many chemists nowadays may not realize the magnitude of this change and the contributions of the pioneers.

For practitioners in the field, high thermal stability and non-hygroscopicity of reagents are key factors for easy handling and a long shelf life. Many *N*-F reagents satisfy this requirement, however, there are limitations as the fluorinating power increases. *N*-Fluoropentachloropyridinium triflate (**5-4v**) and tetrafluoroborate (**5-4w**) rank as the most powerful of the non-hygroscopic and thermally stable *N*-F agents. However, the more powerful *N*-fluoro-2,4,6-trichloro-1,3,5-triazinium tetrafluoroborate (**31-2**) is moisture-sensitive and not easy-to-handle. *N,N’*-Difluoro-1,4-diazoniabicyclo[2.2.2]octane bistetrafluoroborate (**22-1d)** ranks top in the easy-to-handle 1,4-diazoniabicyclo[2.2.]octane salt series. Salt **22-1d** is more powerful than Selectfluor. Unfortunately, synthetic chemists have had little chance to use it because it has not been commercialized due to patent issues despite its simple one-pot process using cheap materials. However, since all the patents have now expired, it is expected that **22-1d** may soon be commercialized.

It is now rare for researchers and laboratories to use molecular fluorine (F_2_) in universities and institutes, because of strict safety regulations, although 20% or 10% F_2_ diluted with N_2_ can be handled without significant problems. As a result, currently reported fluorination reactions have been limited to the use of a few commercially available and inexpensive *N*-F reagents such as Selectfluor and NFSI. As mentioned in this review, there are many other useful *N*-F fluorinating agents and the plentiful supply will satisfy the strong demand for fluorinations. That said, there is still room for improvement or refinement. The authors think that further research and development on *N*-F fluorinating reagents is needed for the advancement of synthetic fluorine chemistry but, for this to happen, it would be highly desirable to develop a safe and convenient F_2_ generator.
